# In-silico design of envelope based multi-epitope vaccine candidate against Kyasanur forest disease virus

**DOI:** 10.1038/s41598-021-94488-8

**Published:** 2021-08-24

**Authors:** Sathishkumar Arumugam, Prasad Varamballi

**Affiliations:** Manipal Institute of Virology, Manipal Academy of Higher Education, Manipal, Karnataka 576104 India

**Keywords:** Biotechnology, Immunology, Microbiology

## Abstract

Kyasanur forest disease virus (KFDV) causing tick-borne hemorrhagic fever which was earlier endemic to western Ghats, southern India, it is now encroaching into new geographic regions, but there is no approved medicine or effective vaccine against this deadly disease. In this study, we did in-silico design of multi-epitope subunit vaccine for KFDV. B-cell and T-cell epitopes were predicted from conserved regions of KFDV envelope protein and two vaccine candidates (VC1 and VC2) were constructed, those were found to be non-allergic and possess good antigenic properties, also gives cross-protection against Alkhurma hemorrhagic fever virus. The 3D structures of vaccine candidates were built and validated. Docking analysis of vaccine candidates with toll-like receptor-2 (TLR-2) by Cluspro and PatchDock revealed strong affinity between VC1 and TLR2. Ligplot tool was identified the intermolecular hydrogen bonds between vaccine candidates and TLR-2, iMOD server confirmed the stability of the docking complexes. JCAT sever ensured cloning efficiency of both vaccine constructs and in-silico cloning into pET30a (+) vector by SnapGene showed successful translation of epitope region. IMMSIM server was identified increased immunological responses. Finally, multi-epitope vaccine candidates were designed and validated their efficiency, it may pave the way for up-coming vaccine and diagnostic kit development.

## Introduction

Kyasanur Forest Disease (KFD) is a southern Indian endemic zoonotic disease caused by KFDV that belongs to the family *Flaviviridae*. National institute of Allergy and Infectious Disease has recognized KFDV as category C priority virus. KFDV was first identified in 1957 from isolates of the sick and dying monkeys of black-faced langur (*Presbytis entellus*) and red-faced bonnet (*Macaca radiate*) species in Kyasanur Forest, state of Karnataka, India^1–4^. Humans are the dead-end host of KFDV life cycle, initial stage KFDV circulates among monkeys, rodents then it transfers into humans by bites of infected ticks, primarily through *Haemaphysalis* sp^5^.

The KFDV is an enveloped virus with icosahedral nucleocapsid, spherical in shape and about 40–65 nm in size. It contains 10,774 bases of positive single stranded RNA, encodes 3146 amino acid. Post-translational cleavage of polyprotein into three structural proteins (Capsid, Membrane and Envelope) and seven non-structural proteins (NS1, NS2A, NS2B, NS3, NS4A, NS4B and NS5)^6,7^.

During 1957–2017, totally 9594 KFDV cases were reported in India^8^. Recently, KFDV outbreak was reported in a new geographic location Sindhudurg district of Maharashtra^9^. In future, it may spread to other parts of India. But still there is no cure for KFDV, currently formalin inactivated vaccine being used as primary strategy for controlling KFD^10^, it gives partial effect against to KFDV infections and provides short term immunity to those have received vaccination^11^. It indicates low efficiency of current vaccine, there is a need for development of new vaccine candidates against KFDV.

There was a study reported the KFDV envelope protein structural variation and different in B-cell epitope antigenicity^12^. There are no studies on KFDV vaccine development, so we tried to construct the epitope-based subunit vaccine which elite T-cell and B-cell immune response upon KFDV infection. T-cells and B-cells drive the process of adaptive immunity and develop immunological memory by recognizing portions of antigens called epitopes. These epitopes contains short amino acid sequence that can induce more direct and specific immune response, which overcome the disadvantages of live attenuated vaccines^13^. In-silico based epitope prediction methods have reduced the time consumption and money spent on false epitope candidates and this tools has been employed to develop multi-epitope vaccine against several diseases such as Dengue, SARS-CoV-2, *Mycobacterium tuberculosis*, *Staphylococcus aureus* etc^14–19^. Hence, the present investigation has adapted this approach for rational design of epitope-based vaccine against KFDV. Here, first time we have designed vaccine candidates from conserved region of KFDV-envelop protein. And we have successfully designed the multi-immunogenic, non-allergic and high cloning efficient multi-epitope vaccine candidates by utilizing immune-informatics and bioinformatics tools.

## Results

### Conserved region of KFDV envelope protein

**Figure 1:**
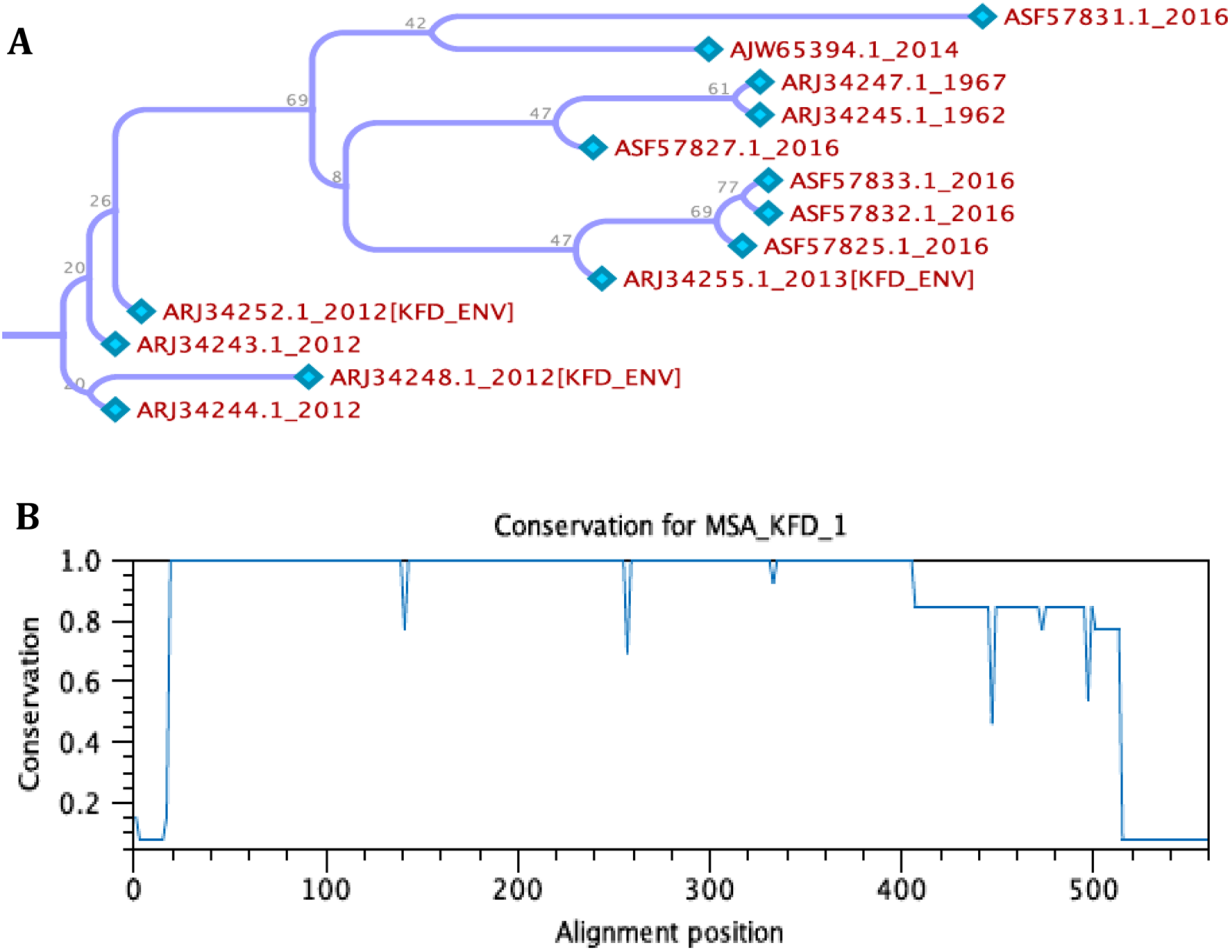
Multiple sequence alignment of KFDV envelop protein by CLC workbench. (**A**) Phylogenetic tree of KFDV envelope protein. (**B**) Conservation graph of KFDV envelop protein sequence (value 1.0 is highest conserved).

The multiple sequence alignment analysis (Supplementary File 1) reveals that except few amino acids, the entire KFDV envelope protein sequences remain conserve. Phylogenetic tree was constructed from the alignment file, shows separate cluster of 2012 KFDV envelope protein, rest of the sequence were almost the same (Fig. 1A). Based on the conserve score (Supplementary File 2) conservation graph has been drawn, value 1.0 on the Y-axis represents more conserved (Fig. 1B). Amino acids, which got > 0.90 conserve score was considered as a conserved region and taken into further study, conserved amino acid sequence and their position were given in Table 1.

**Table 1:** Conserved amino acid sequences of KFDV envelop protein predicted by CLC workbench.

Name	Conserved amino acid sequence	Position	Length
CS1	TRVSLVLELGGCVTLTAEGKPSVDVWLDDIHQENPAKTREYCLHAKLANSKVAARCPAMGPATLPEEHQASTVCRRDQSDRGWGNHCGLFGKGSIVACAKFSCETKKKATGYVYDVNKITYV	19–140	121
CS2	KVEPHTGDYLAANESHSNRKTASFTTQSEKTILTLGDYGDISLTCRVTSGVDPAQTVVLELDKTAEHLPKAWQVHRDWFEDLSLPWRHEGAQEWNHADRLVEFGEPHAVKMDIFN	142–256	114
CS3	GDQTGILLKSLAGVPVANIEGSKYHLQSGHVTCDVGLEKLKMKGMTYTVCEGSKFAWKRPPTDSGHDTVVMEVTYTGSKPCRIPVRAVAHGEPNVNVASLITPNPSMETTGGGFVELQLPPGDNIIYVGELSHQWFQKGSTIGRVLEKT	258–406	148

### Conserved regions of KFDV envelope protein containing T-cell and B-cell epitopes

Here we found that the conserved region contains epitopes for T-cell and B-cell targets. Totally 35 epitopes were obtained IEDB-MHC-I tools (Supplementary File 3), whereas the MAPPP tool produced 300 epitopes from 43 human HLA alleles (Supplementary File 4). IEDB-MHC-I top 10 percentile epitopes were also predicted in MAPPP tool, they are listed in Table 2. Combined predictor of proteasomal cleavage, TAP transport, and MHC process has produced 34 epitopes (Supplementary File 5), and top 10 ranked epitopes were listed in Table 3. There are 20 MHC class II epitopes (Supplementary File 6) were identified by IEDB-MHC-II tools, whereas MHC2Pred tool recognized 504 epitopes (Supplementary File 7) on the conserved region of the envelope protein. Based on IEDB = MHC-II percentile > 0.10 (FILT-1) and MHC2Pred epitopes which scored > 0.60 as well having overlap sequence with FILT-1, totally19 epitopes were selected as MHC II targets (Table 4). B-cell liner epitopes prediction tools ABCpred, BCpred and SVMTriP were identified 15, 13 and 4 epitopes respectively (Table 5).

**Table 2:** List of top 10 MHC1 binding epitopes.

S. no	Allele	Peptide length	Sequence	Proteasome score	TAP score	MHC score	Processing score	Total score
1	HLA-B*35:01	10	YVYDVNKITY	1.48	1.39	− 1.5	2.87	1.37
2	HLA-A*30:02	9	**KTILTLGDY**	1.24	1.26	− 1.23	2.51	1.27
3	HLA-B*40:01	9	REYCLHAKL	1.54	0.55	− 0.93	2.09	1.16
4	HLA-B*40:01	9	LELGGCVTL	1.76	0.39	− 1.04	2.15	1.11
5	HLA-A*30:02	9	KVEPHTGDY	0.96	1.26	− 1.37	2.22	0.86
6	HLA-B*35:01	9	LPPGDNIIY	1.45	1.11	− 1.77	2.57	0.8
7	HLA-A*26:01	9	ETKKKATGY	1.2	1.17	− 1.59	2.37	0.78
8	HLA-B*58:01	10	**KTAEHLPKAW**	1.5	0.37	− 1.12	1.87	0.75
9	HLA-B*15:01	10	YVYDVNKITY	1.48	1.39	− 2.18	2.87	0.69
10	HLA-A*23:01	10	IYVGELSHQW	1.31	0.55	− 1.18	1.86	0.68

Overlapping sequence found in both IEDB-MHC I and MAPPP tool were bolded.

**Table 3:** List of IEDB-tool gerenerated top 10 MHC1 binding epitopes based on combined score of proteasome, cleave, TAP and MHC processing score.

S. no	Allele	Length	Peptide	IEDB-MHC-I percentile rank
1	HLA-B*57:01	9	KAWQ**VHRDW**	0.06
2	HLA-A*30:02	9	**KTILTLGDY**	0.06
3	HLA-B*57:01	10	**KTAEHLPKAW**	0.09
4	HLA-A*11:01	9	**STIGRVLEK**	0.11
5	HLA-A*33:01	10	**DYGDISLTCR**	0.15
6	HLA-B*58:01	10	**KTAEHLPKAW**	0.17
7	HLA-B*07:02	10	**KPCRIPVRAV**	0.18
8	HLA-A*11:01	9	**ASFTTQSEK**	0.2
9	HLA-B*58:01	9	**KAWQVHR**DW	0.2
10	HLA-A*11:01	9	**VTCDVGLE**K	0.2

Overlapping epitope sequence found in IEDB-MHC I, IEDB-MHC-I-combined and MAPPP tool were bolded.

**Table 4:** List of MHC2 binding epitopes by IEDB-tool.

S. no	Allele	Method	Peptide	IEDB-percentile rank/MHC2PRED peptide score
1	HLA-DQA1*01:01/DQB1*05:01	ANN/IEDB	**KAWQVHRDWFEDLSL**	0.02
2	HLA-DRB1*0802	SVM/MHC2PRED	**KAWQVHRDW**	0.672
3	HLA-DR3	SVM/MHC2PRED	P**KAWQVHRD**	0.819
4	HLA-DR15	SVM/MHC2PRED	LP**KAWQVHR**	0.839
5	HLA-DQ8	SVM/MHC2PRED	**RDWFEDLSL**	1.797
6	HLA-DQA1*01:01/DQB1*05:01	ANN/IEDB	**AWQVHRDWFEDLSLP**	0.02
7	HLA-DRB5*0101	SVM/MHC2PRED	**AWQVHRDWF**	0.836
8	HLA-DQB1*0301	SVM/MHC2PRED	PK**AWQVHRD**	0.897
9	HLA-DQ4	SVM/MHC2PRED	**DWFEDLSLP**	0.847
10	HLA-DQA1*01:01/DQB1*05:01	ANN/IEDB	**WQVHRDWFEDLSLPW**	0.02
11	HLA-DRB5*0101	SVM/MHC2PRED	**AWQVHRDWF**	0.836
12	HLA-DQB1*0301	SVM/MHC2PRED	**WFEDLSLPW**	0.751
13	HLA-DR3	SVM/MHC2PRED	**FEDLSLPWR**	1.168
14	HLA-DQA1*01:01/DQB1*05:01	ANN/IEDB	**QVHRDWF**EDLSLPWR	0.04
15	HLA-DRB5*0101	SVM/MHC2PRED	AW**QVHRDWF**	0.836
16	HLA-DQA1*01:01/DQB1*05:01	ANN/IEDB	VHRDWF**EDLSLPWRH**	0.05
17	HLA-DQ6	SVM/MHC2PRED	**EDLSLPWRH**	0.639
18	HLA-DRB3*01:01	ANN/IEDB	KPSVDV**WLDDIHQEN**	0.1
19	HLA-DQB1*03	SVM/MHC2PRED	**WLDDIHQEN**	0.917

Overlapping epitope sequence found in IEDB-MHC-II and MHC2Pred tools were bolded.

**Table 5:** List of B-cell epitopes predicated by ABCpred, BCpred and SVMTriP tools.

S. no	ABCpred		BCpred		SVMTriP	
	Epitope	Score	Epitope	Score	Epitope	Score
1	AQ**TVVLELDKTAEH**	0.91	AARCPAMGPATLPE	0.999	KTREYCLHAKLANS	1
2	ASTVCRRDQSDRGW	0.87	L**DDIHQENPAKTRE**	0.944	TDSGHDTVVMEVTY	1
3	DKTAEHLPKAWQVH	0.87	RDQSDRGWGNHCGL	0.895	WFQKGSTIGRVLEK	0.886
4	**DDIHQENPAKTRE**Y	0.85	KKKATGYVYDVNKI	0.877	**TVVLELDKTAEH**LP	0.834
5	**NVNVASLITPNP**SM	0.83	WRHEGAQEWNHADR	0.902		
6	VGLEKLKMKGMTYT	0.83	LTLGDYGDI**SLTCR**	0.845		
7	VACAKFSCETKKKA	0.82	**AANESHSNRKTASF**	0.843		
8	**SLTCR**VTSGVDPAQ	0.82	EFGEPHAVKMDIFN	0.652		
9	GVPVANIEGSKYHL	0.82	GGGFVEL**QLPPGDN**	0.98		
10	FGKGSIVACAKFSC	0.81	KFAW**KRPPTDSGHD**	0.98		
11	**KRPPTDSGHD**TVVM	0.81	**TGSKPCRIPVRAVA**	0.974		
12	**QLPPGDN**IIYVGEL	0.81	SHQWFQKGSTIGRV	0.761		
13	**AANESHSNRKTASF**	0.8	EP**NVNVASLITPNP**	0.723		
14	**SNRKTASF**TTQSEK	0.78				
15	**TGSKPCRIPVRAVA**	0.78				

Overlapping sequence of B-cell epitopes sequences were bolded.

### Selected epitopes display cross-protection against Alkhumra hemorrhagic fever virus

Uniportkb-human BLAST analysis showed similarity with four human proteins such as shish-7, Isoform 2 of Ribonuclease T2, Ribonuclease T2 and Extra-cellular ribonuclease with E-value of 2e−1, 7.7e−1, 2.1e0 and 2.1e0 respectively (Supplementary File 8). These similarity hits can be ignored, as E-value is ≥ 0.1, because as rule of thumb an E-value should be < 10–4 to assure the homology. Whereas UniProtKB BLAST revealed that selected epitopes were sharing the highest similarity with Alkhumra Hemorrhagic Fever Virus (AHFV)^20^ polyprotein, E-value was 1.7e−43 (Supplementary File 9). So, the proposed vaccine candidates also confer immunity against AHFV.

### Multi-epitope vaccine candidate sequences their allergenicity and antigenicity

Totally, two multiple-epitope vaccine candidates were designed from selected high ranked, multi-immunogenic and over lapping epitope sequences. Vaccine construct 1 (VC1) composed with an adjuvant protein β defensin, 6 MHC- I epitope and 11 B-cell epitopes. Vaccine constructs 2 (VC2) composed of VC1 and 4 MHC-II epitopes. Complete information of vaccine construct has given in Table 6. Vaccine candidates VC1 and VC2 were found to be non-allergic in behavior, VC1 got a higher antigenic score (0.6667) and identified as a better antigen, while VC2 antigenic score was 0.5835.

**Table 6:** Multi-epitope vaccine candidate sequence.

Vaccine construct	Composition of construct	Complete sequence of designed construct	Allegicity (AlgPred)	VaxiJen score status
VC1	β defensin adjuvant, high scored MHC-1 epitopes and B-cell epitopes	MSYLRNSTSLVRVPKAFLKPFRVCCFVIAGHGGIINTLQKYYCRVRGGRCAVLSCLPKEEQIGKCSTRGRKCCRRKK**EAAAK**LELGGCVTL**GGGS**REYCLHAKL**GGGS**YVYDVNKITY**GGGS**KVEPHTGDY**GGGS**KTILTLGDY**GGGS**DYGDISLTCR**GGGS**KTAEHLPKAW**KK**LDDIHQENPAKTREYCLHAKLANS**KK**AARCPAMGPATLPE**KK**ASTVCRRDQSDRGWGNHCGL**KK**VACAKFSCETKKKATGYVYDVNKI**KK**LTLGDYGDISLTCRVTSGVDPAQAQTVVLELDKTAEHLPKAWQVH	Non-allergen	0.6667 Portable antigen
VC2	β defensin adjuvant, high scored MHC-I epitopes, B-Cell epitopes, MHC-II epitopes	MSYLRNSTSLVRVPKAFLKPFRVCCFVIAGHGGIINTLQKYYCRVRGGRCAVLSCLPKEEQIGKCSTRGRKCCRRKK**EAAAK**LELGGCVTL**GGGS**REYCLHAKL**GGGS**YVYDVNKITY**GGGS**KVEPHTGDY**GGGS**KTILTLGDY**GGGS**DYGDISLTCR**GGGS**KTAEHLPKAW**KK**LDDIHQENPAKTREYCLHAKLANS**KK**AARCPAMGPATLPE**KK**ASTVCRRDQSDRGWGNHCGL**KK**VACAKFSCETKKKATGYVYDVNKI**KK**LTLGDYGDISLTCRVTSGVDPAQAQTVVLELDKTAEHLPKAWQVH**GPGPG**LPKAWQVHRDWFEDLSLPWRHKPSVDVWLDDIHQEN	Non-allergen	0.5835 Portable antigen

### Physiochemical and solubility properties of vaccine construct

The molecular weight (MW) of constructed vaccine candidates VC1 and VC2 were found to 34.6 kDa and 39.4 kDa respectively. Theoretical pI value 9.38(VC1) and 9.25 (VC2) were observed and proteins are expected to alkaline in nature. Estimated half-life for both VC1 and VC2 candidates were 30 h in mammalian reticulocytes in vitro. Instability index were found to be 32.86 and 33.61 for VC1 and VC2 respectively and confirmed as stable proteins. Negative GRAVY values of VC1 (− 0.455) and VC2 (− 0.511) indicate they are hydrophilic in nature and they could interact with water. PROSO II predicted VC1 as soluble protein with score of 0.667, whereas VC2 scored 0.489 and it identified as insoluble protein, obtained physiochemical characteristics are strengthening potency of vaccine candidates.

### 3D-strucure of designed vaccine candidates

**Figure 2:**
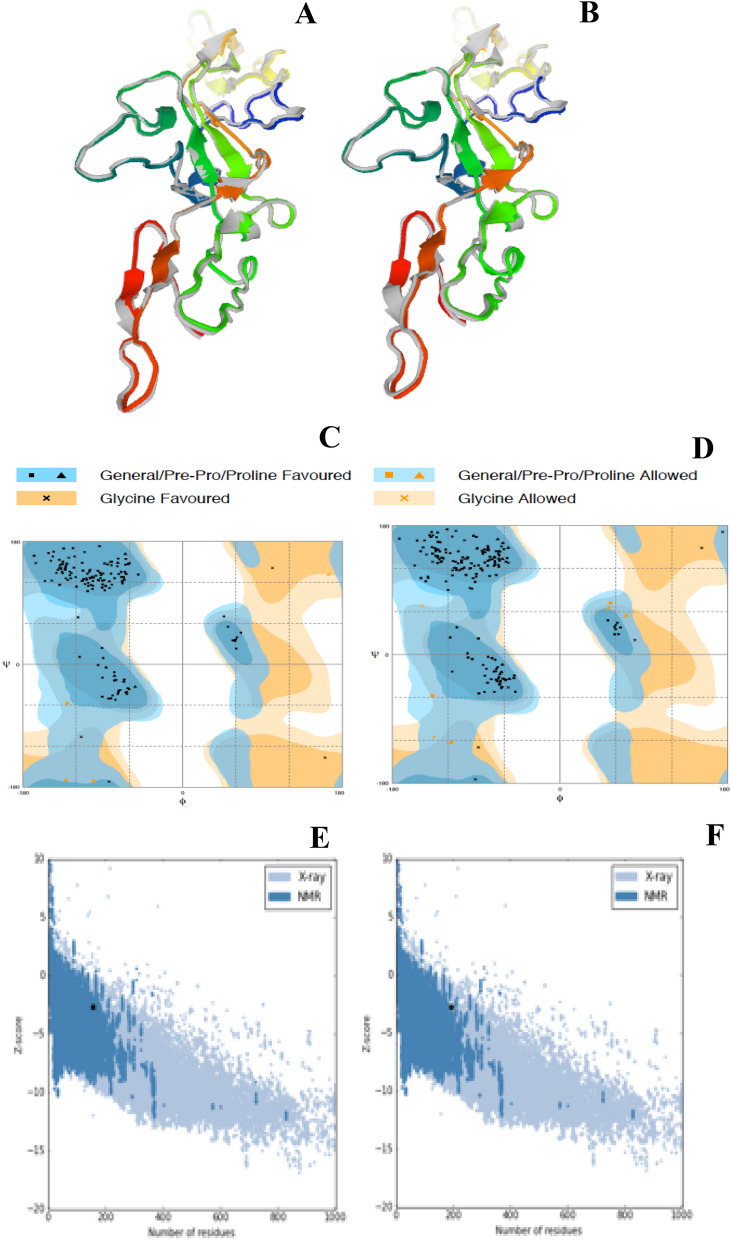
3D-modeling, refinement and validation of multi epitope vaccine constructs. GalaxyRefine generated Refined structure of (**A**) VC1 and (**B**) VC2 construct. RAMPAGE produced of Ramachandran plot analysis of (**C**) refined VC1 structure and (**D**) refined VC2 structure. ProSA-web shows Z-score of (**E**) − 2.74 for VC1 and (**F**) − 2.66 for VC2.

Phyre2 server has built three-dimensional structure of multi-epitope vaccine candidates. The cryo-em structure of tick-borne encephalitis virus complex was used as template for VC1 and VC2 tertiary structure, and both the model got 100% score in terms of model confidence. Initial refinement of vaccine candidates VC1 and VC2 on ModRefiner have produced refine structure with TM-score of 0.9507 and 0.9622 respectively. Further refinement of VC1 and VC2 were done with GalaxyRefine server, based on quality score the best refined structure were chosen and named as VC1R and VC2R respectively. Those were used as ligand molecules in docking study (Fig. 2A, B), quality scores of ligands were given in Table 7.

**Table 7:** GalaxyRefine server quality scores of vaccine candidates.

Vaccine model	GDT-HA	RMSD	MolProbity	Clash score	Poor rotamers	Rama score
VC1_model3 (VC1R)	0.9143	0.488	1.567	8.4	0.0	97.4
VC2_model2 (VC2R)	0.9477	0.437	1.961	13.5	0.0	95.4

The Ramachandran plot analysis of modeled vaccine candidates revealed that 97.4% of VC1R protein residues were in favored region and 2.6% of residues in allowed region (Fig. 2C). Similarly, 95.9% of VC2R protein residues were in favored region and 4.1% of the residues in allowed region (Fig. 2D). None of the amino acid residues were fallen in outlier region, which indicates the good quality of protein structure. ProSA-web analysis of yielded Z-score of − 2.74 and − 2.66 for vaccine candidates of VC1R and VC2R respectively. (Fig. 2E, F).

### Identification of binding energy between multi-epitope vaccine candidates and TLR2

**Figure 3:**
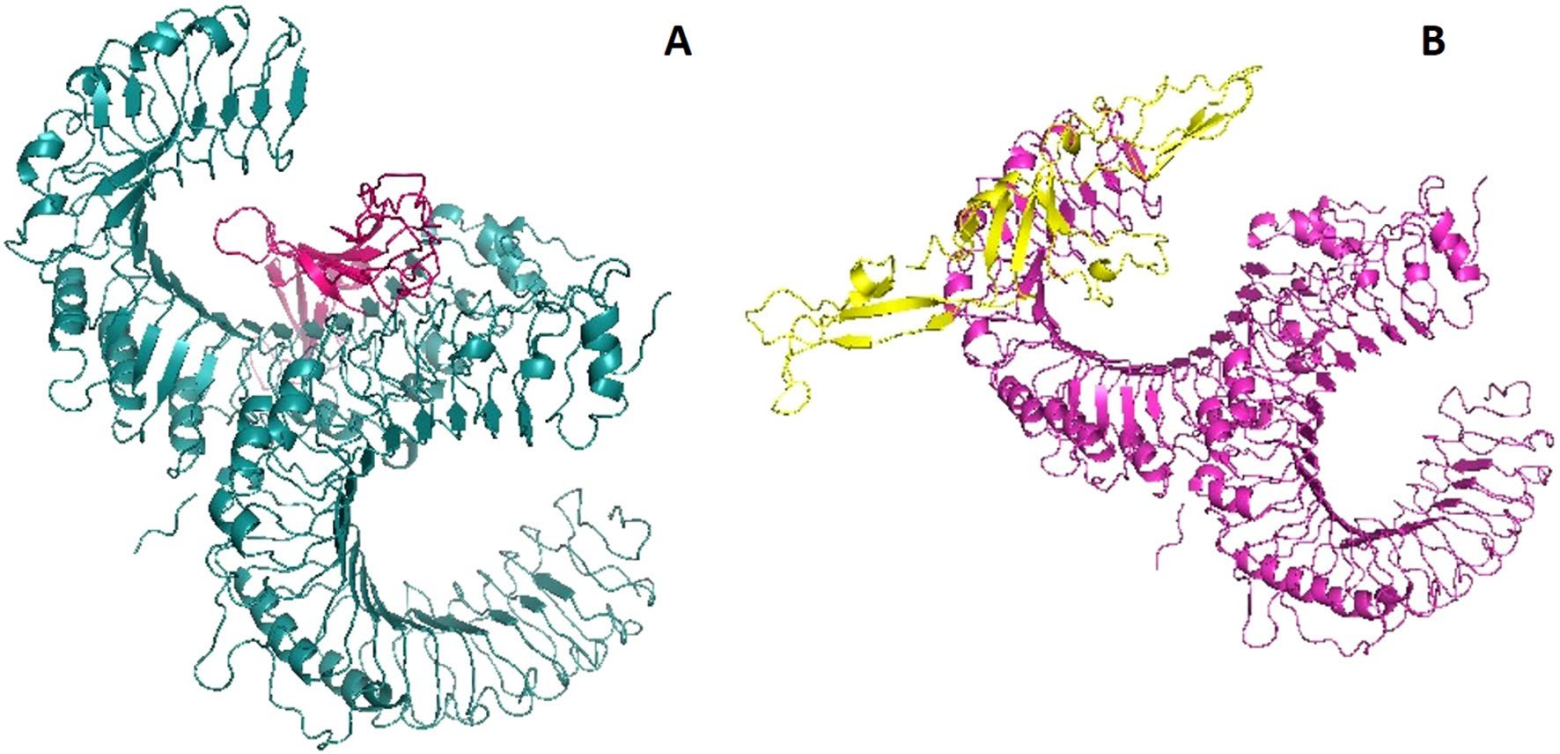
Identification of binding energy by Cluspro docking server (**A**) VC1 and TLR2 complex (**B**) VC2 and TLR2 complex.

Docking analysis by Cluspro and PatchDock has revealed that both vaccine candidates (VC1 and VC2) have strong binding capacity with TLR2. Cluspro protein–protein docking result page displayed 10 best-docked confirmations, the top ranked model from balanced coefficient module was downloaded in PDB format and used for LigPlot and iMODS analysis. TLR2 with VC1 exhibited lowest energy value of − 9473.6 kcal/mol, whereas VC2 showed − 876.4 kcal/mol (Fig. 3).

PatchDock server produced transformation file (posture of 3D-rotational angle) then it was rescored and global energy was obtained by FireDock. The best docking posture was ranked based on the global energy, top ranked docking score, energy contributed by hydrogen bond and Van der waals forces were listed Table 8. The highest global energy of TLR2 with VC1 and VC2 was found to be − 55.71 and − 49.10 respectively.

**Table 8:** FireDock binding energy scores of VC1 and VC2 with TLR2.

Receptor	Ligand	Global energy*	HB^#^	Attractive VdW^$^
TLR2	VC1	− 55.71	− 7.41	− 33.41
	VC2	− 49.10	− 6.25	− 37.01

*—the binding energy of the receptor and ligand on specific posture of 3 rotational angle, ^#^—the contribution of the hydrogen bonds to the global binding energy, ^$^—the contribution of the van der Waals forces to the global binding energy.

### Interaction and stability of TLR2 and Vaccine candidates

**Figure 4:**
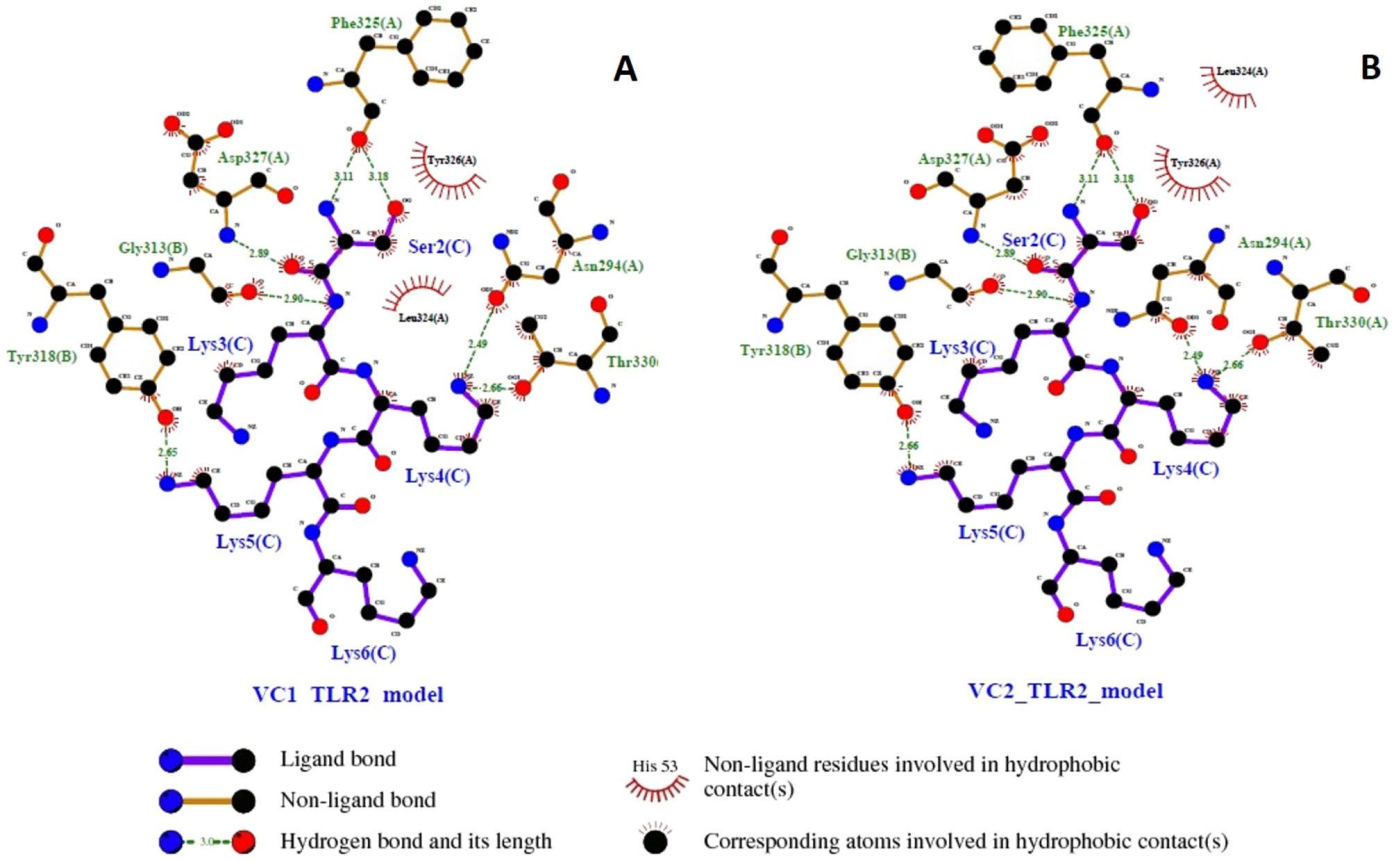
Ligplot analysis showing intermolecular hydrogen bonds in (**A**) VC1 and TLR2 complex (**B**) VC2 and TLR2 complex.

Interaction of TLR2 and Vaccine candidates were visualized by LigPlot tool. Hydrogen bonds were highlighted in dash line (Fig. 4A), totally seven hydrogen bonds were found between TLR2 with VC1. The receptor’s residue Asn294 showed highest binding affinity with 2.49 Å distance and least binding energy was found towards Phe325 with 3.18 Å distance. VC2 construct also showed identical interaction with TLR2 residues (Fig. 4B).

**Figure 5:**
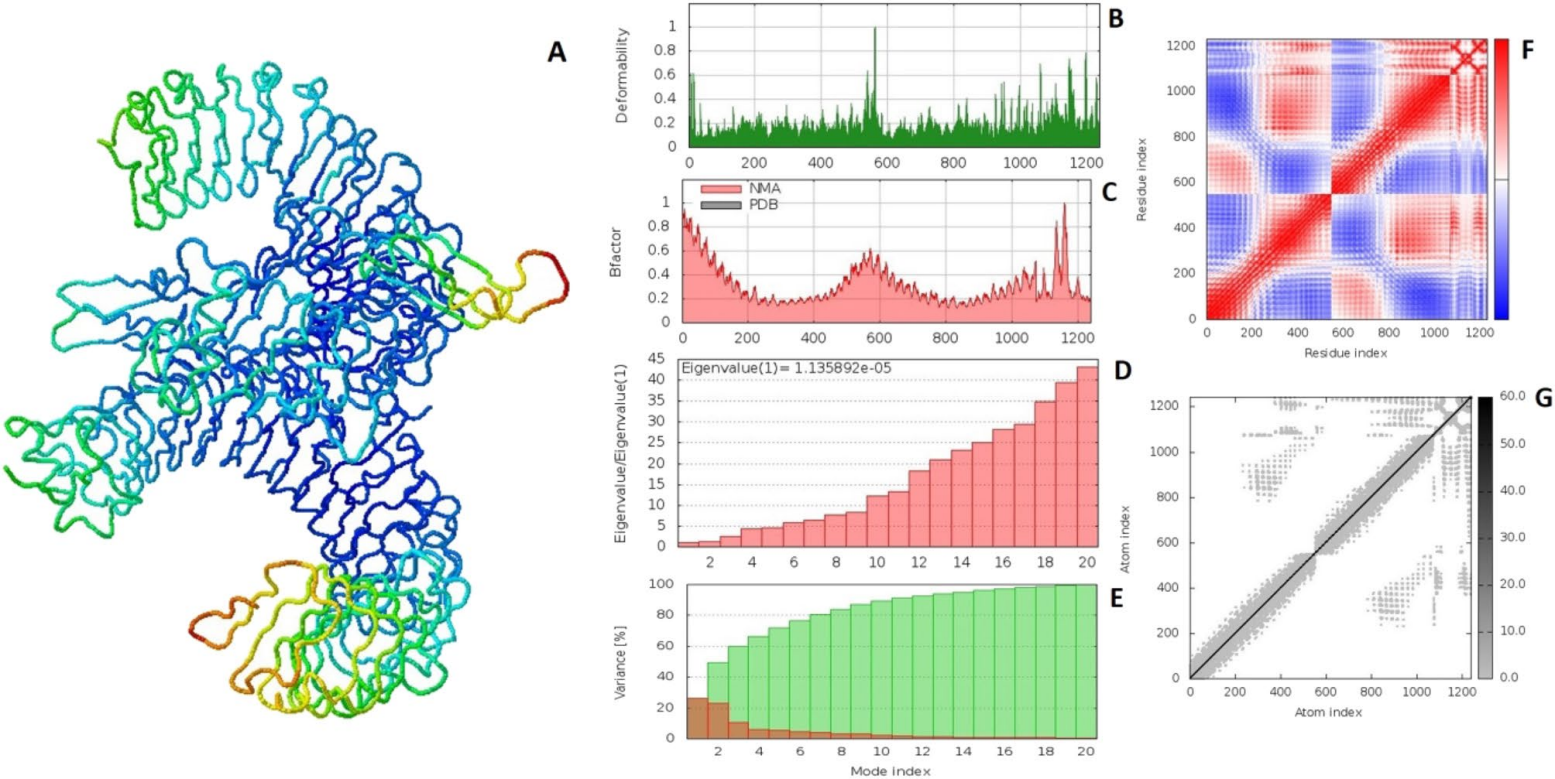
Molecular dynamics simulation of VC1 and TLR2 complex by iMODS server. (**A**) VC1 and TLR2 docking complex. (**B**) Main-chain deformability. (**C**) B-factor values. (**D**) The eigenvalue. (**E**) Variance. (**F**) Co-variance map. (**G**) Elastic network of model.

**Figure 6:**
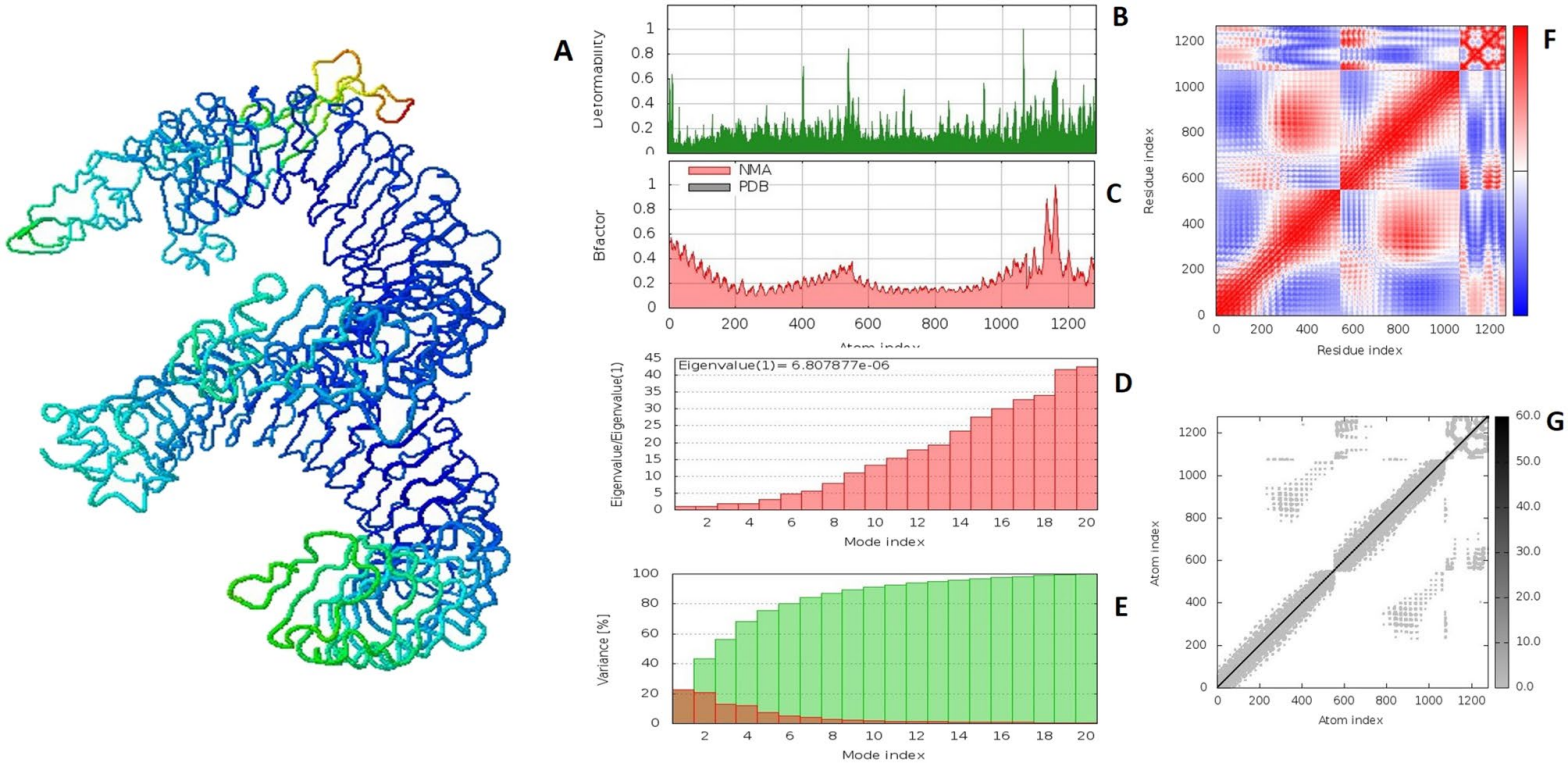
Molecular dynamics simulation of VC2 and TLR2 complex by iMODS server. (**A**) VC2 and TLR2 docking complex. (**B**) Main-chain deformability. (**C**) B-factor values. (**D**) The eigenvalue. (**E**) Variance. (**F**) Co-variance map. (**G**) Elastic network of model.

Molecular dynamics simulation study was done by iMODS server to check the stability and physical movement of atoms in docking complex. Simulation were performed in normal mode analysis and obtained results of VC1-TLR2 and VC2-TLR2 docking complexes were depicted in Figs. 5 and 6 respectively. The deformability graph of both complexes was illustrated in Figs. 5B and 6B, hinges are highlighting the deformability regions in the complex. The B-factor (Figs. 5C, 6C) calculates root mean square value and represents the uncertainty of each atom in the docking complex. Eigenvalues of VC1-TLR2 and VC2-TLR2 docking complexes were found to be 1.135892 × 10^−5^ and 6.807877 × 10^−6^ respectively (Figs. 5D, 6D). The variance matrix graph of residues displayed in Figs. 5E and 6E. Covariance matrix indicates (Figs. 5F, 6F) coupling between pairs of residue experience correlated (red), uncorrelated (white) and anti-correlated (blue) motions. The elastic of docking complexes was shown in Fig. 5G, it explains the relation between the atoms (darker gray). iMODS simulation results are suggest that vaccine constructs and TLR2 complexes are stable.

### Codon optimization and in silico cloning of KFD-VC1 and KFD-VC2

**Figure 7:**
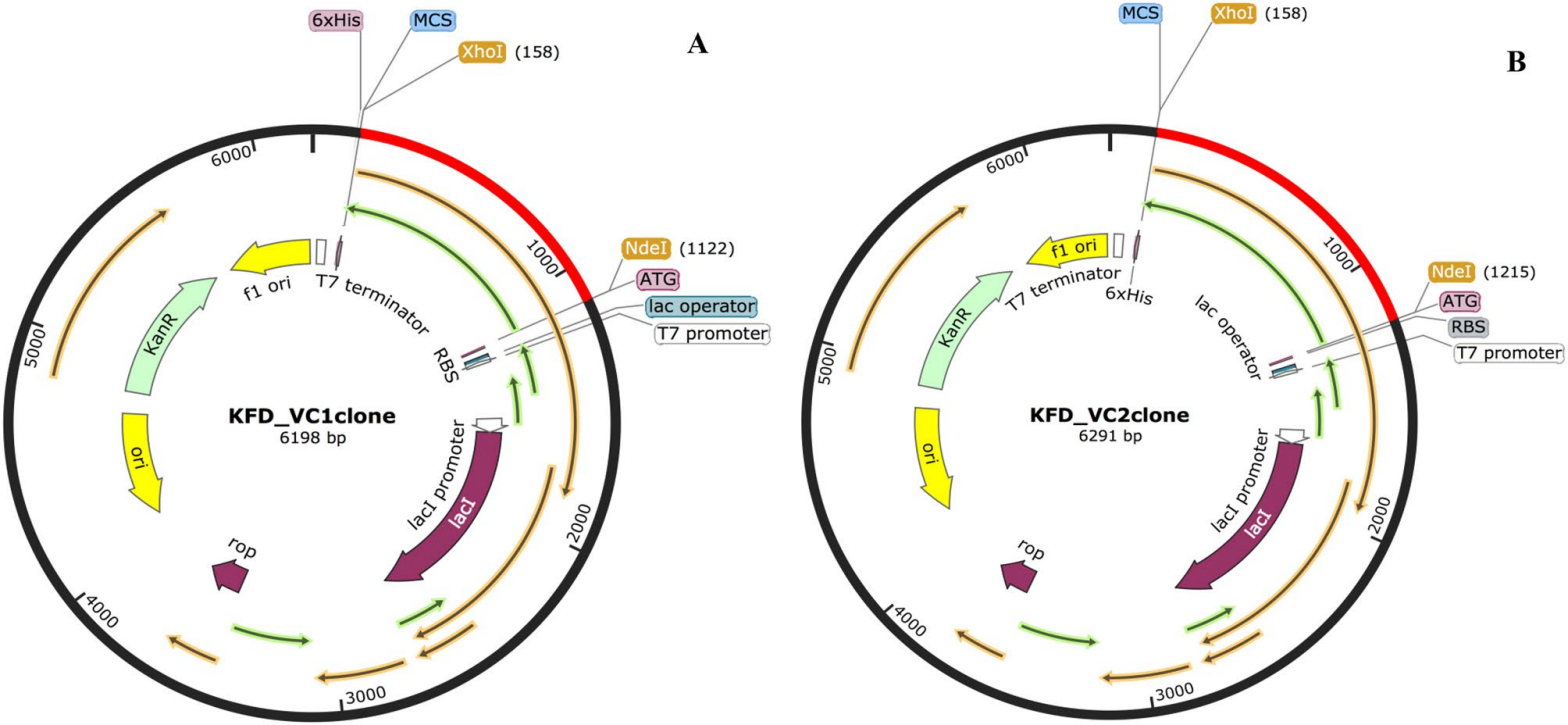
In silico cloning of multiepitope subunit vaccine sequence into pET30a (+) expression vector by SnapGene tool, red color part represents vaccine sequence and black circle represent vector sequence. Restriction sites are heighted in yellow color and translation sequence parts were mention in green color arrows.

Java Codon Adaptation Tool was used to check codon optimism of vaccine candidates in *Escherichia coli* (strain k12) expression system. It revealed that VC1 and VC2 multi-epitope vaccine construct composed of 927 and 1050 nucleotides respectively. The Codon Adaptation Index (CAI) was observed, 1.0 for VC1 and VC2 was 0.98. The GC content of VC1 found to be 51.13% and VC2 was 52.19%. The obtained values indicate that both vaccine candidates were having cloning efficiency. SnapGene tool was used for in-silico insertion of adapted codon sequence of vaccine construct into pET30a (+) expression vector between XhoI and NedI restriction sites and clones were obtained successfully (Fig. 7).

### Immune response simulation

**Figure 8:**
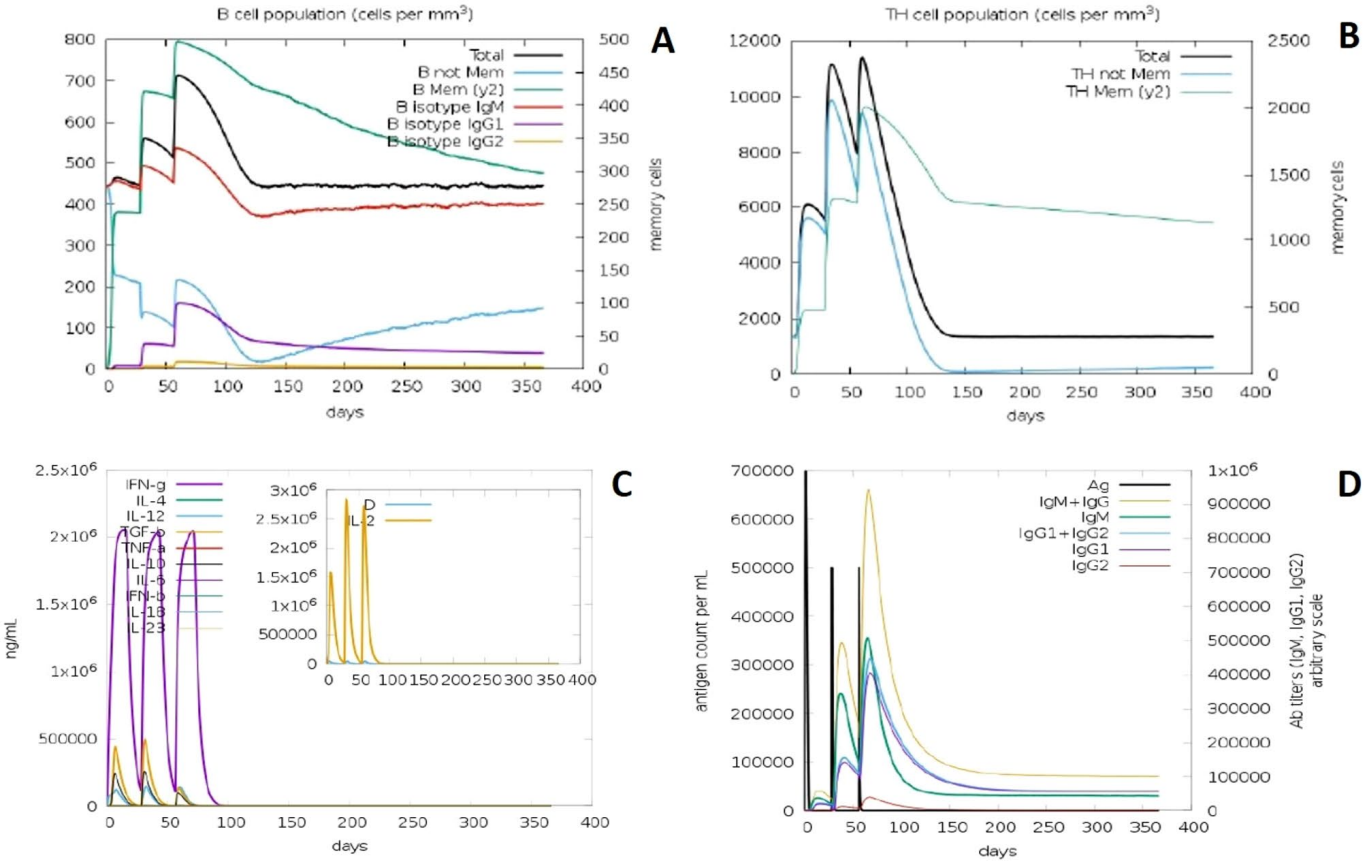
In silico immune simulation of VC1 by IMMSIM server. (**A**) Population of B lymphocytes, y2 represents scale of memory B cells. (**B**) Population of T lymphocytes, y2 represents scale of memory T cells. (**C**) Concentration of cytokines and interleukins (insect plot shows elevated level of IL-2). (**D**) Immunoglobulin production upon antigen exposure.

**Figure 9:**
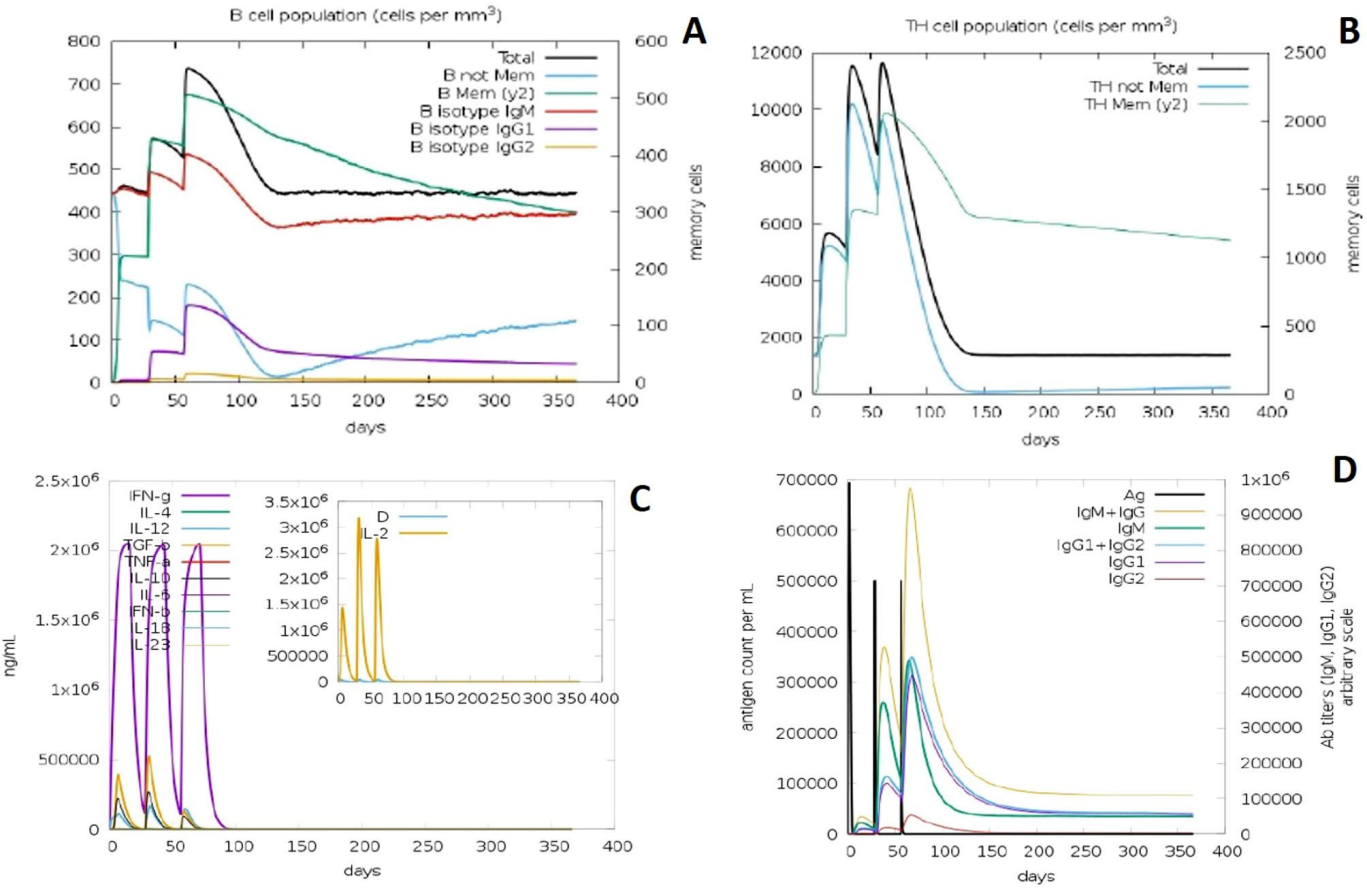
In silico immune simulation of VC2 by IMMSIM server. (**A**) Population of B lymphocytes, y2 represents scale of memory B cells. (**B**) Population of T lymphocytes, y2 represents scale of memory T cells. (**C**) Concentration of cytokines and interleukins (insect plot shows elevated level of IL-2). (**D**) Immunoglobulin production upon antigen exposure.

The immunogenic profile of constructed vaccine candidates was obtained from C-IMMSIM server. It found that our vaccine candidates could able to elicit both humoral and cellular mediated immune responses (Figs. 8, 9). Simulation results showed that secondary and tertiary immune responses is higher than primary response. Antigenic molecules were found to be cleared off after three doses of vaccination, on the other hand B and T memory cell population got increased at the maximum of 500 cells/mm^3^ and 2000 cells/mm^3^ respectively, this feature makes our construct VC1 and VC2 as suitable KFDV vaccine candidates. This study also found that elevated level of cytokines such as IFN-γ and IL-2, which important for inhibition of viral replication and T-cell mediated immunity. Increased trend of IgM and IgG antibody titer was observed after third injection, at the same time antigen level were decreased. Above observed immune elicit properties ensured that vaccine constructs will be effective in human subjects.

## Discussion

This genome era enabled with bulk inflow of genomics and proteomics information of almost all clinically important organisms. It facilitates us to choosing the targets for drug design, identifying new strains or drug resistance, diagnosis kit development and personalized medicine etc. Similarity, computational approaches simplified vaccine development process, vaccine informatics is a fast-growing field where in-silico vaccine design ventures are possible. Previous report on KFDV immune response has suggested to target both T cell and B cells mediated immune response for successful KFDV vaccine development^21^. We have applied few immune informatic tools to identify the epitope which elicit both cellular and humoral immune responses.

In our study, KFDV envelope protein sequence was chosen as target for epitope prediction. The envelope protein of flaviviruses is major protective antigen and consists of three domains EI, EII and EIII. Especially EIII domain contains specific and sub-complex specific neutralizing epitopes, moreover it is easy to express by recombinant techniques^22^. Another challenge is genetic diversity of virus that resulted in immune escape, always-recombinant vaccines design prefers to target conserved antigenic regions of virus, it might accelerates better immune response^23^. The KFDV vaccinated individuals were reported for disease occurrence, it may because of variations in circulating virus^24^. Therefore, we picked the conserved region of KFDV envelope protein sequence, to overcome the genetic diversity of viral strains.

So far, there is no experimental study on identification of T-cell and B-cell epitopes. Here we have identified both of them by using immuno-informatics tools. Epitopes or viral antigens arise the specific immune responses in the body, which induce adaptive immune responses of T-cells mediated cellular immunity and B-cells/antibody mediated humoral immunity^25,26^. All three MHC-I epitope prediction tools identified KTILTLGDY and KTAEHLPKAW. Also, MHC II epitope KAWQVHRDWFEDLSL overlap with MHC class I epitope KAWQVHRDW, it may serve as target for both CD8 + and CD4 + cells. B-cell epitopes KRPPTDSGHD, TVVLELDKTAEH, DDIHQENPAKTR, AANESHSNRKTASF, TGSKPCRIPVRAVA were identified by any of two-prediction tools out of three.

The high ranked, multi-immunogenic and overlapped epitopes were linked together and multi-epitope construct was obtained. Typical purified proteins are not inherently immunogenic, it requires adjuvant to accelerate the innate immune system and enhance vaccine potency^27^. We have added β-defensin at N terminal end of the multi-epitope vaccine construct. β-defensin is known to induce lymphokines production which promotes T-cell mediated cellular immunity and antigen-specific Ig production^28^.

Totally, we made two multi-epitopes vaccines construct against KFDV, 3D structure, and refinement of the vaccine candidates were achieved. The Ramachandran plots of built structures showed that none of the residues were in outlier region, > 95% of the amino acids fallen in favored region. It clearly indicates that modeled structures of vaccine candidates are good in quality.

TLRs plays vital role in innate immunity, especially they detect the virus and activates innate immunity followed by adaptive immunity. Several reports confirmed that TLR2 act as a host sensor to identifying viral envelop proteins and subsequently activates the innate immune system^29–31^. Our docking studies suggest that designed vaccine construct have the binding capacity with TLR2, both Cluspro and PatchDock have identified VC1 as potential binder with TLR2. Hydrogen bonds plays viral role in protein-receptor interaction, and it determines the stability of complex^32,33^. Intramolecular interactions between vaccine construct and TLR2 was visualized by LigPlot^+^. Immune simulation study explains the efficiency of vaccine construct to elicit the immune response. Induction of memory B-cells and T-cells is one of the criteria to be a successful vaccine candidate^34–36^. Each injection of VC1 and VC2 increased the level of memory B-cells and T-cells and this population level is sustaining after the third injection (Figs. 7, 8). Also, the elevated level of IFN-γ and IL2 is exhibiting the capacity of vaccine constructs to establish the antiviral state, in general IFN-γ involves in antiviral replication and act as main effector molecule in cell mediated immunity. *Escherichia coli* is most preferable choice of the host to obtain more quantity of recombinant vaccine protein^37^. Java Codon Adaptation tool displayed codon adaptability index as > 0.99% and GC content as > 50% for both vaccine candidates, it confirmed the favorable content of vaccine constructs for high-level protein expression in *E. coli* host.

## Conclusion

The present study has made an attempt to design the multi-epitope vaccine against KFDV by using immune-informatics tools. To our knowledge this is first report for identifying T-cell and B-cell targeting epitopes of KFDV. The designed chimeric vaccine peptide could elicit immune response, but still it need to be tested on in-vitro and in-vivo models. Interestingly, our constructed vaccine has cross-protection effect against AHFV. This study has given foresight for development of new prophylaxis for KFDV control in India, and gives the directions in selecting epitopes for KFDV vaccine development.

## Material and methods

### Retrieval of amino acid sequence and conservation analysis

Totally 13 KFDV envelope protein sequences (GenBank ID: ARJ34245.1, ARJ34247.1, ASF57827.1, ASF57825.1, ASF57832.1, ASF57833.1, ARJ34243.1, ARJ34244.1, ARJ34252.1, ARJ34248.1, ASF57831.1, ARJ34255.1, AJW65394.1) which reported during 1962 to 2016, were retrieved from NCBI database (http://www.ncbi.nlm.nih.gov).

Multiple sequence alignment (MSA) of retrieved sequences were performed in CLCworkbench (https://www.qiagenbioinformatics.com/products/clc-main-workbench/), alignment file (.aln) was generated from all retrieved envelop protein sequences, neighbor-joining method was used to the phylogenetic tree and conservation score of each amino acids were obtained.

### Prediction of T-cell and B-cell epitopes

Eight different immunoinformatic tools were used to predict T-cell (MHC class I and MHC class II) and B-cell epitope regions on KFDV envelope protein. Three different tools (IEDB-MHC-I, IEDB- combined and MAPPP) were used to obtain MHC class I epitope, proteasomal cleavage score, Transporter of Antigenic Peptide (TAP) and MHC scores. MHC class II epitope was prediction by IEDB- MHC-II and MHC2Pred tools. B-cell epitopes were identified by ABCpred, BCpred, and SVMTriP tools, the detailed information on tools algorithm, URL site and threshold values were in Table 9.

**Table 9:** List of T-cell and B-cell prediction tools used in the study.

Epitope target	Tool	Algorithm	Threshold value	URLsite
T-cell MHC class I	IEDB-MHC-I	Artificial network	Percentile rank > 0.5	http://tools.iedb.org/mhci/^38–42^
	IEDB-combined predictor	Artificial network	Total score > 0.5	http://tools.iedb.org/processing/^43,44^
	MAPPP	SYFPEITHI	> 1.0	http://www.mpiib-berlin.mpg.de/MAPPP/binding.html^45,46^
T-cell MHC class II	IEDB-MHC-II	Artificial network	Percentile rank > 0.5	http://tools.iedb.org/mhcii/^47,48^
	MHC2Pred	Support vector machine (SVM)	> 1.5	http://crdd.osdd.net/raghava/mhc2pred/^49^
B-cell	ABCpred	Artificial network	> 0.5	https://webs.iiitd.edu.in/raghava/abcpred/ABC_submission.html^50^
	BCpred	Artificial network	Specificity 75%	http://ailab.ist.psu.edu/bcpred/predict.html^51–53^
	SVMTriP	Support vector machine (SVM)	Epitope length 14	http://sysbio.unl.edu/SVMTriP/prediction.php^54^

### Self-antigen and cross-protection analysis

Uniport-BLAST tool (https://www.uniprot.org/blast/) was used to checked all top ranked T-cell and B-cell epitope sequences for self-antigen and cross-protection analysis. Self–antigen or similarity of human proteins with predicted epitopes were identified by Uniport- BLAST search against uniportkb_human databases. And UniProtKB database BLAST search was used to identify the cross protection of selected epitopes with other pathogenic organisms.

### Construction of multi-epitope vaccine candidate

Based on high score, multi-immunogenic and overlapping sequence of T-cell and B-cell epitopes were conjugated to construct the multi-epitope vaccine candidates. Construct of vaccine has started with adjuvant β defensin (ACK99045.1) peptide sequence, in order to augment the immunogenicity of the vaccine candidate. Adjuvant was connected to MHC-I epitopes with EAAAK linker, every individual epitopes of MHC-I were linked by GGGS and all MHC-II epitopes assembled with GPGPG linker. Whereas B-cell epitopes were combined by KK linker. Usage of the linker between two epitopes will increase the immunogenicity and function of the vaccine constructs^54^.

### Allergenicity and antigenicity of the vaccine candidate

Algpred (https://webs.iiitd.edu.in/raghava/algpred/submission.html) used to predict the allergenicity of vaccine candidate based on the similarity of known epitope. The vaccine candidate sequence was uploaded into the server, the IgE epitope and PID-BLAST search on allergen representative peptide algorithms were chosen for allergen prediction^55^. Antigenicity capacity of the vaccine candidate were evaluate by VaxiJen 2.0 server (http://www.ddg-pharmfac.net/vaxijen/VaxiJen/VaxiJen.html)^56^.

### Physiochemical properties and solubility prediction

Physiochemical parameters of vaccine candidate such as molecular weight, theoretical pI, instability index, aliphatic index, estimated half-life and grand average of hydropathicity (GRAVY) were identified by ProtParam (https://web.expasy.org/protparam/) tool^57^. PROSO II server (http://mbiljj45.bio.med.uni-muenchen.de:8888/prosoII/prosoII.seam) was used to evaluate the solubility of vaccine candidates^58^.

### 3D structure prediction, refinement and validation

Phyre2 server (http://www.sbg.bio.ic.ac.uk/~phyre2/html/page.cgi?id=index)^59^ used to build the 3D-structure of the designed vaccine candidates. The server is build the 3D model based on homologues with known protein structure, and produce accurate model of about 70% of the domains in a typical genome. The obtained 3D structures of designed multi-epitope vaccine candidates were subjected into two-step refinement. Initial refinement was performed in ModRefiner (https://zhanglab.ccmb.med.umich.edu/ModRefiner/)^60^, an atomic-level protein structure refinement tool. This program makes protein into full-atomic relaxation, where initial model or reference model does not restrain the refined model. This improves both global and local structures with more accurate side chain positions, better hydrogen-bonding networks, and fewer atomic overlaps. Then second refinement was done with GalaxyRefine server (http://galaxy.seoklab.org/cgi-bin/submit.cgi?type=REFINE)^61,62^, it performs repeated structure perturbation to side-chains and for secondary structure elements and loops are also applied followed by overall structural relaxation by molecular dynamics simulation. The obtained 3D-structures of vaccine candidates were validated in RAMPAGE server (http://mordred.bioc.cam.ac.uk/~rapper/rampage.php)^63^ and ProSA-web (https://prosa.services.came.sbg.ac.at/prosa.php)^64,65^. ProSA recognize the errors in experimental and theoretical model of protein structure and z-core will tell about the quality of the protein structure.

### Molecular docking of designed vaccine candidates with TLR2

TLR plays viral role in innate immune response upon the viral infections, especially TLR2 were reported for recognition of viral envelope protein. Hence, TLR2 (PDB ID: 2Z7X) was selected as target protein against to constructed vaccine candidates. ClusPro 2.0 server (https://cluspro.org)^66^ and PatchDock (https://bioinfo3d.cs.tau.ac.il/PatchDock/php.php)^67^ were used for this purpose.

ClusPro 2.0 server was used to predict the binding energy between TLR-2 and vaccine construct, experiment was performed by uploading PDB files of receptors and ligands into server and submitted with default parameters. PatchDock was also performed at default settings, top 1000 transformed docking position generated from PatchDock server was re-scored by FireDock server (http://bioinfo3d.cs.tau.ac.il/FireDock/php.php)^68,69^ and global energy of each vaccine candidate with TLR2 was obtained.

### Ligplot analysis and molecular dynamic simulation of Docking complex

Interaction of TLR2 with vaccine candidates were assessed by Ligplot, docking complexes resulted from cluspro analysis were saved as PDB format and uploaded into Ligplot tool^70^ and intermolecular interactions such as hydrogen bonds, hydrophobic contact was obtained in the form of 2D representation.

The molecular dynamics simulation was performed to check the stability of docking complexes. The iMODS (http://imods.chaconlab.org/)^71–73^ web-server was used to calculate B-factor (disorder of atoms), structural deformability and eigenvalue.

### In silico cloning optimization of KFD multi-epitope vaccine candidates

The Java Codon Adaptation Tool (http://www.prodoric.de/JCat) was used for reverse translation and codon optimization of designed vaccine candidates^74^. Codon optimization was executed in order to express the KFD multi epitope vaccine construct in *E. coli* (strain K12). The output files were checked for Codon Adaptation Index (CAI) (> 0.8–1.0), and GC content (30–70%), to ensure transcription and translational efficiency of designed vaccine construct. Resulted optimized codon sequence of vaccine candidates were introduced with XhoI and NdeI restriction sties at N-terminal and C-terminal ends respectively. SnapGene (www.snapgene.com) was utilized to insert the vaccine sequence in expression vector pET-30a(+), between XhoI and NdeI cloning site, final clones of vaccine candidates VC1 and VC2 were obtained.

### Immune response simulation

Immune response profile of the constructed vaccine candidates was evaluated by C-IMMSIM server^75^. Experiment was performed by uploading PDB files of vaccine constructs, random speed, simulation volume and simulation steps were set as 12,345, 50 and 1100 respectively. Three insilco injections were given at the time steps of 1, 84 and 168 respectively (1-time step is equal to 8 h in real life), with no LPS and maintained minimum 30 days of time interval between two injections.

## Supplementary Information

Supplementary Tables.
