# Supplementary material for: In-silico design of envelope based multi-epitope vaccine candidate against Kyasanur forest disease virus
Source: Sci Rep. 2021 Aug 24;11:17118. doi: 10.1038/s41598-021-94488-8 (PMC8384868; doi:10.1038/s41598-021-94488-8)
Supplement: Supplementary file 1 — Supplementary Tables. [file 41598_2021_94488_MOESM1_ESM.pdf]

Supplementary information

In-Silico design of envelope based multi-epitope vaccine candidate against Kyasanur Forest Disease Virus.

Sathishkumar Arumugam*

Manipal Institute of Virology, Manipal Academy of Higher Education, Manipal-576104, Karnataka, India.

(E.mail: sathishkumar.a@manipal.edu)

Supplementary Table 1:

Multiple sequence alignment of KFDV envelope protein sequence by CLC workbench

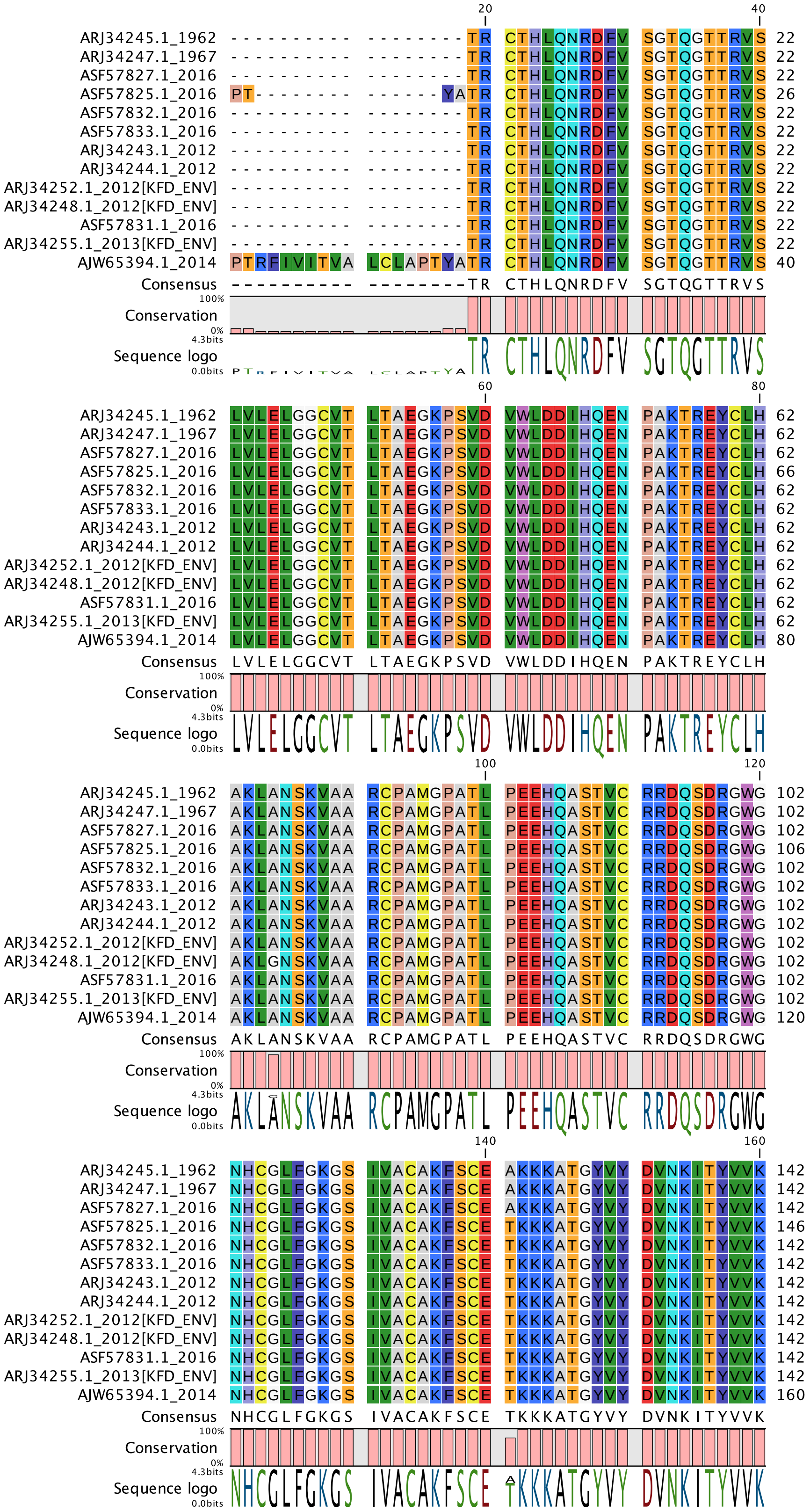

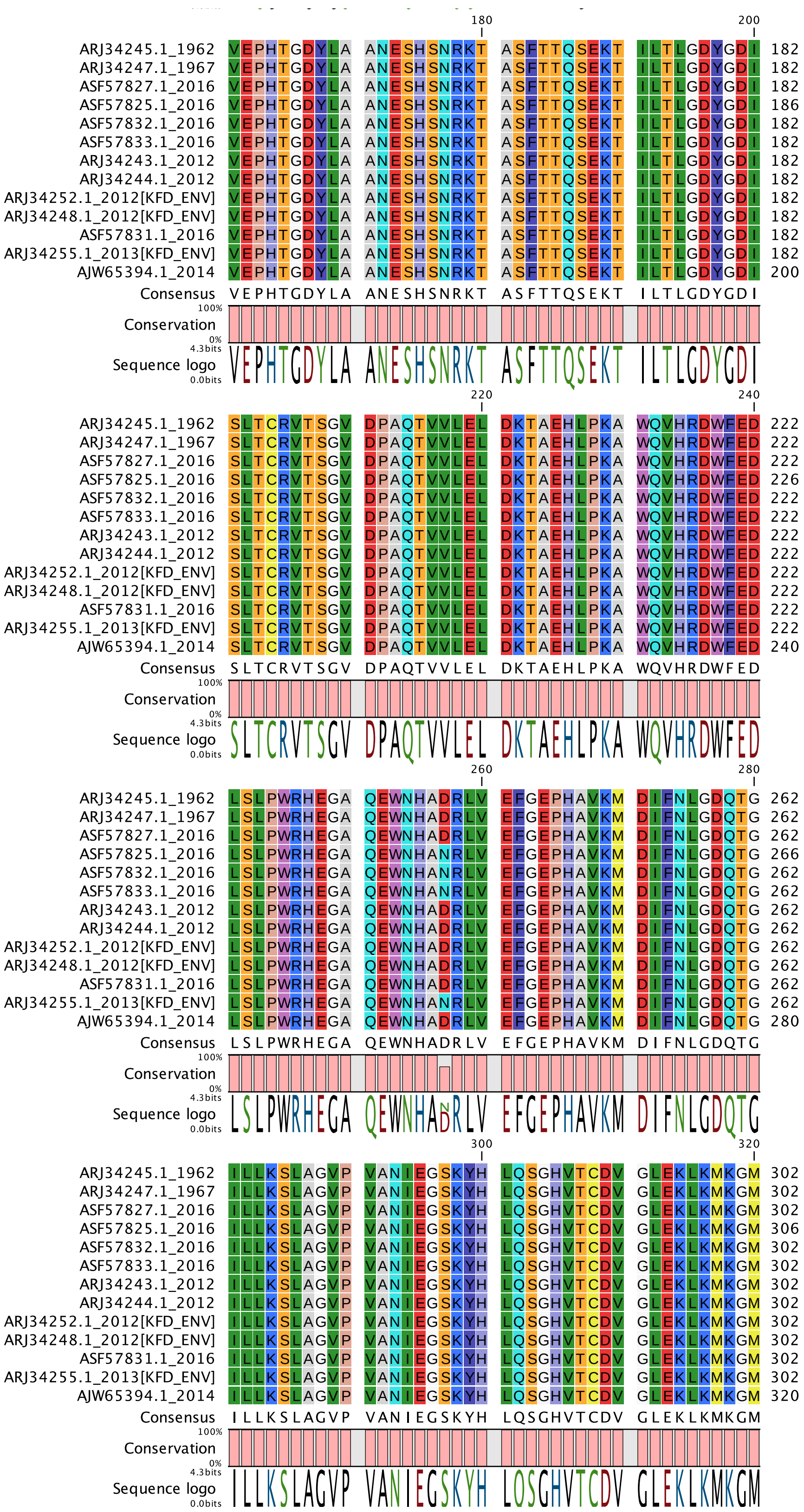
180
200
ARJ34245.1_1962 VEPHTGDYLA ANESHSNRKT ASFTTQSEKT ILTLGDYGDI 182
ARJ34247.1_1967 VEPHTGDYLA ANESHSNRKT ASFTTQSEKT ILTLGDYGDI 182
ASF57827.1_2016 VEPHTGDYLA ANESHSNRKT ASFTTQSEKT ILTLGDYGDI 182
ASF57825.1_2016 VEPHTGDYLA ANESHSNRKT ASFTTQSEKT ILTLGDYGDI 186
ASF57832.1_2016 VEPHTGDYLA ANESHSNRKT ASFTTQSEKT ILTLGDYGDI 182
ASF57833.1_2016 VEPHTGDYLA ANESHSNRKT ASFTTQSEKT ILTLGDYGDI 182
ARJ34243.1_2012 VEPHTGDYLA ANESHSNRKT ASFTTQSEKT ILTLGDYGDI 182
ARJ34244.1_2012 VEPHTGDYLA ANESHSNRKT ASFTTQSEKT ILTLGDYGDI 182
ARJ34252.1_2012[KFD_ENV] VEPHTGDYLA ANESHSNRKT ASFTTQSEKT ILTLGDYGDI 182
ARJ34248.1_2012[KFD_ENV] VEPHTGDYLA ANESHSNRKT ASFTTQSEKT ILTLGDYGDI 182
ASF57831.1_2016 VEPHTGDYLA ANESHSNRKT ASFTTQSEKT ILTLGDYGDI 182
ARJ34255.1_2013[KFD_ENV] VEPHTGDYLA ANESHSNRKT ASFTTQSEKT ILTLGDYGDI 182
AJW65394.1_2014 VEPHTGDYLA ANESHSNRKT ASFTTQSEKT ILTLGDYGDI 200
Consensus VEPHTGDYLA ANESHSNRKT ASFTTQSEKT ILTLGDYGDI
100%
Conservation
0%
4.3bits
Sequence logo
0.0bits
220
240
ARJ34245.1_1962 SLTCRVTSGV DPAQTVVLEL DKTAEHLPKA WQVHRDWFED 222
ARJ34247.1_1967 SLTCRVTSGV DPAQTVVLEL DKTAEHLPKA WQVHRDWFED 222
ASF57827.1_2016 SLTCRVTSGV DPAQTVVLEL DKTAEHLPKA WQVHRDWFED 222
ASF57825.1_2016 SLTCRVTSGV DPAQTVVLEL DKTAEHLPKA WQVHRDWFED 226
ASF57832.1_2016 SLTCRVTSGV DPAQTVVLEL DKTAEHLPKA WQVHRDWFED 222
ASF57833.1_2016 SLTCRVTSGV DPAQTVVLEL DKTAEHLPKA WQVHRDWFED 222
ARJ34243.1_2012 SLTCRVTSGV DPAQTVVLEL DKTAEHLPKA WQVHRDWFED 222
ARJ34244.1_2012 SLTCRVTSGV DPAQTVVLEL DKTAEHLPKA WQVHRDWFED 222
ARJ34252.1_2012[KFD_ENV] SLTCRVTSGV DPAQTVVLEL DKTAEHLPKA WQVHRDWFED 222
ARJ34248.1_2012[KFD_ENV] SLTCRVTSGV DPAQTVVLEL DKTAEHLPKA WQVHRDWFED 222
ASF57831.1_2016 SLTCRVTSGV DPAQTVVLEL DKTAEHLPKA WQVHRDWFED 222
ARJ34255.1_2013[KFD_ENV] SLTCRVTSGV DPAQTVVLEL DKTAEHLPKA WQVHRDWFED 222
AJW65394.1_2014 SLTCRVTSGV DPAQTVVLEL DKTAEHLPKA WQVHRDWFED 240
Consensus SLTCRVTSGV DPAQTVVLEL DKTAEHLPKA WQVHRDWFED
100%
Conservation
0%
4.3bits
Sequence logo
0.0bits
260
280
ARJ34245.1_1962 LSLPWRHEGA QEWNHADRLV EFGEPHAVKM DIFNLGDQTG 262
ARJ34247.1_1967 LSLPWRHEGA QEWNHADRLV EFGEPHAVKM DIFNLGDQTG 262
ASF57827.1_2016 LSLPWRHEGA QEWNHADRLV EFGEPHAVKM DIFNLGDQTG 262
ASF57825.1_2016 LSLPWRHEGA QEWNHANRLV EFGEPHAVKM DIFNLGDQTG 266
ASF57832.1_2016 LSLPWRHEGA QEWNHANRLV EFGEPHAVKM DIFNLGDQTG 262
ASF57833.1_2016 LSLPWRHEGA QEWNHANRLV EFGEPHAVKM DIFNLGDQTG 262
ARJ34243.1_2012 LSLPWRHEGA QEWNHADRLV EFGEPHAVKM DIFNLGDQTG 262
ARJ34244.1_2012 LSLPWRHEGA QEWNHADRLV EFGEPHAVKM DIFNLGDQTG 262
ARJ34252.1_2012[KFD_ENV] LSLPWRHEGA QEWNHADRLV EFGEPHAVKM DIFNLGDQTG 262
ARJ34248.1_2012[KFD_ENV] LSLPWRHEGA QEWNHADRLV EFGEPHAVKM DIFNLGDQTG 262
ASF57831.1_2016 LSLPWRHEGA QEWNHADRLV EFGEPHAVKM DIFNLGDQTG 262
ARJ34255.1_2013[KFD_ENV] LSLPWRHEGA QEWNHANRLV EFGEPHAVKM DIFNLGDQTG 262
AJW65394.1_2014 LSLPWRHEGA QEWNHADRLV EFGEPHAVKM DIFNLGDQTG 280
Consensus LSLPWRHEGA QEWNHADRLV EFGEPHAVKM DIFNLGDQTG
100%
Conservation
0%
4.3bits
Sequence logo
0.0bits
300
320
ARJ34245.1_1962 ILLKSLAGVP VANIEGSKYH LQSGHVTCDV GLEKLKMKGM 302
ARJ34247.1_1967 ILLKSLAGVP VANIEGSKYH LQSGHVTCDV GLEKLKMKGM 302
ASF57827.1_2016 ILLKSLAGVP VANIEGSKYH LQSGHVTCDV GLEKLKMKGM 302
ASF57825.1_2016 ILLKSLAGVP VANIEGSKYH LQSGHVTCDV GLEKLKMKGM 306
ASF57832.1_2016 ILLKSLAGVP VANIEGSKYH LQSGHVTCDV GLEKLKMKGM 302
ASF57833.1_2016 ILLKSLAGVP VANIEGSKYH LQSGHVTCDV GLEKLKMKGM 302
ARJ34243.1_2012 ILLKSLAGVP VANIEGSKYH LQSGHVTCDV GLEKLKMKGM 302
ARJ34244.1_2012 ILLKSLAGVP VANIEGSKYH LQSGHVTCDV GLEKLKMKGM 302
ARJ34252.1_2012[KFD_ENV] ILLKSLAGVP VANIEGSKYH LQSGHVTCDV GLEKLKMKGM 302
ARJ34248.1_2012[KFD_ENV] ILLKSLAGVP VANIEGSKYH LQSGHVTCDV GLEKLKMKGM 302
ASF57831.1_2016 ILLKSLAGVP VANIEGSKYH LQSGHVTCDV GLEKLKMKGM 302
ARJ34255.1_2013[KFD_ENV] ILLKSLAGVP VANIEGSKYH LQSGHVTCDV GLEKLKMKGM 302
AJW65394.1_2014 ILLKSLAGVP VANIEGSKYH LQSGHVTCDV GLEKLKMKGM 320
Consensus ILLKSLAGVP VANIEGSKYH LQSGHVTCDV GLEKLKMKGM
100%
Conservation
0%
4.3bits
Sequence logo
0.0bits

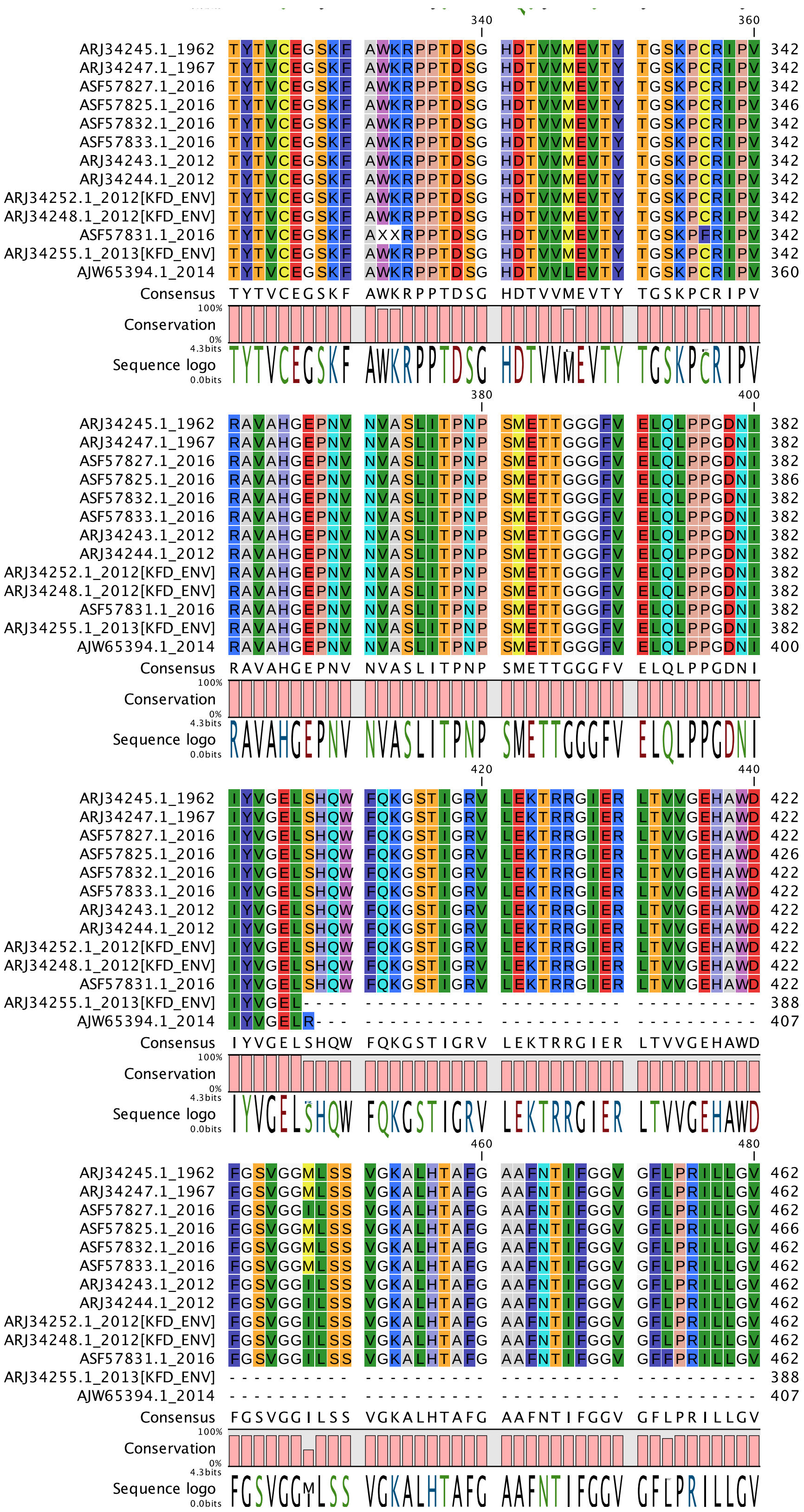
340 360
ARJ34245.1_1962 TYTVCEGSKF AWKRPPTDSG HDTVVMEVTY TGSKPCRIPV 342
ARJ34247.1_1967 TYTVCEGSKF AWKRPPTDSG HDTVVMEVTY TGSKPCRIPV 342
ASF57827.1_2016 TYTVCEGSKF AWKRPPTDSG HDTVVMEVTY TGSKPCRIPV 342
ASF57825.1_2016 TYTVCEGSKF AWKRPPTDSG HDTVVMEVTY TGSKPCRIPV 346
ASF57832.1_2016 TYTVCEGSKF AWKRPPTDSG HDTVVMEVTY TGSKPCRIPV 342
ASF57833.1_2016 TYTVCEGSKF AWKRPPTDSG HDTVVMEVTY TGSKPCRIPV 342
ARJ34243.1_2012 TYTVCEGSKF AWKRPPTDSG HDTVVMEVTY TGSKPCRIPV 342
ARJ34244.1_2012 TYTVCEGSKF AWKRPPTDSG HDTVVMEVTY TGSKPCRIPV 342
ARJ34252.1_2012[KFD_ENV] TYTVCEGSKF AWKRPPTDSG HDTVVMEVTY TGSKPCRIPV 342
ARJ34248.1_2012[KFD_ENV] TYTVCEGSKF AWKRPPTDSG HDTVVMEVTY TGSKPCRIPV 342
ASF57831.1_2016 TYTVCEGSKF AXXRPPTDSG HDTVVMEVTY TGSKPFRIPV 342
ARJ34255.1_2013[KFD_ENV] TYTVCEGSKF AWKRPPTDSG HDTVVMEVTY TGSKPCRIPV 342
AJW65394.1_2014 TYTVCEGSKF AWKRPPTDSG HDTVVLEVTY TGSKPCRIPV 360
Consensus TYTVCEGSKF AWKRPPTDSG HDTVVMEVTY TGSKPCRIPV
Conservation
Sequence logo
380 400
ARJ34245.1_1962 RAVAHGEPNV NVASLITPNP SMETTGGGFV ELQLPPGDNI 382
ARJ34247.1_1967 RAVAHGEPNV NVASLITPNP SMETTGGGFV ELQLPPGDNI 382
ASF57827.1_2016 RAVAHGEPNV NVASLITPNP SMETTGGGFV ELQLPPGDNI 382
ASF57825.1_2016 RAVAHGEPNV NVASLITPNP SMETTGGGFV ELQLPPGDNI 386
ASF57832.1_2016 RAVAHGEPNV NVASLITPNP SMETTGGGFV ELQLPPGDNI 382
ASF57833.1_2016 RAVAHGEPNV NVASLITPNP SMETTGGGFV ELQLPPGDNI 382
ARJ34243.1_2012 RAVAHGEPNV NVASLITPNP SMETTGGGFV ELQLPPGDNI 382
ARJ34244.1_2012 RAVAHGEPNV NVASLITPNP SMETTGGGFV ELQLPPGDNI 382
ARJ34252.1_2012[KFD_ENV] RAVAHGEPNV NVASLITPNP SMETTGGGFV ELQLPPGDNI 382
ARJ34248.1_2012[KFD_ENV] RAVAHGEPNV NVASLITPNP SMETTGGGFV ELQLPPGDNI 382
ASF57831.1_2016 RAVAHGEPNV NVASLITPNP SMETTGGGFV ELQLPPGDNI 382
ARJ34255.1_2013[KFD_ENV] RAVAHGEPNV NVASLITPNP SMETTGGGFV ELQLPPGDNI 382
AJW65394.1_2014 RAVAHGEPNV NVASLITPNP SMETTGGGFV ELQLPPGDNI 400
Consensus RAVAHGEPNV NVASLITPNP SMETTGGGFV ELQLPPGDNI
Conservation
Sequence logo
420 440
ARJ34245.1_1962 IYVGELSHQW FQKGSTIGRV LEKTRRGIER LTVVGEHAWD 422
ARJ34247.1_1967 IYVGELSHQW FQKGSTIGRV LEKTRRGIER LTVVGEHAWD 422
ASF57827.1_2016 IYVGELSHQW FQKGSTIGRV LEKTRRGIER LTVVGEHAWD 422
ASF57825.1_2016 IYVGELSHQW FQKGSTIGRV LEKTRRGIER LTVVGEHAWD 426
ASF57832.1_2016 IYVGELSHQW FQKGSTIGRV LEKTRRGIER LTVVGEHAWD 422
ASF57833.1_2016 IYVGELSHQW FQKGSTIGRV LEKTRRGIER LTVVGEHAWD 422
ARJ34243.1_2012 IYVGELSHQW FQKGSTIGRV LEKTRRGIER LTVVGEHAWD 422
ARJ34244.1_2012 IYVGELSHQW FQKGSTIGRV LEKTRRGIER LTVVGEHAWD 422
ARJ34252.1_2012[KFD_ENV] IYVGELSHQW FQKGSTIGRV LEKTRRGIER LTVVGEHAWD 422
ARJ34248.1_2012[KFD_ENV] IYVGELSHQW FQKGSTIGRV LEKTRRGIER LTVVGEHAWD 422
ASF57831.1_2016 IYVGELSHQW FQKGSTIGRV LEKTRRGIER LTVVGEHAWD 422
ARJ34255.1_2013[KFD_ENV] IYVGEL---- ---------- ---------- ---------- 388
AJW65394.1_2014 IYVGELR--- ---------- ---------- ---------- 407
Consensus IYVGELSHQW FQKGSTIGRV LEKTRRGIER LTVVGEHAWD
Conservation
Sequence logo
460 480
ARJ34245.1_1962 FGSVGGMLSS VGKALHTAFG AAFNTIFGGV GFLPRILLGV 462
ARJ34247.1_1967 FGSVGGMLSS VGKALHTAFG AAFNTIFGGV GFLPRILLGV 462
ASF57827.1_2016 FGSVGGILSS VGKALHTAFG AAFNTIFGGV GFLPRILLGV 462
ASF57825.1_2016 FGSVGGMLSS VGKALHTAFG AAFNTIFGGV GFLPRILLGV 466
ASF57832.1_2016 FGSVGGMLSS VGKALHTAFG AAFNTIFGGV GFLPRILLGV 462
ASF57833.1_2016 FGSVGGMLSS VGKALHTAFG AAFNTIFGGV GFLPRILLGV 462
ARJ34243.1_2012 FGSVGGILSS VGKALHTAFG AAFNTIFGGV GFLPRILLGV 462
ARJ34244.1_2012 FGSVGGILSS VGKALHTAFG AAFNTIFGGV GFLPRILLGV 462
ARJ34252.1_2012[KFD_ENV] FGSVGGILSS VGKALHTAFG AAFNTIFGGV GFLPRILLGV 462
ARJ34248.1_2012[KFD_ENV] FGSVGGILSS VGKALHTAFG AAFNTIFGGV GFLPRILLGV 462
ASF57831.1_2016 FGSVGGILSS VGKALHTAFG AAFNTIFGGV GFFPRILLGV 462
ARJ34255.1_2013[KFD_ENV] ---------- ---------- ---------- ---------- 388
AJW65394.1_2014 ---------- ---------- ---------- ---------- 407
Consensus FGSVGGILSS VGKALHTAFG AAFNTIFGGV GFLPRILLGV
Conservation
Sequence logo

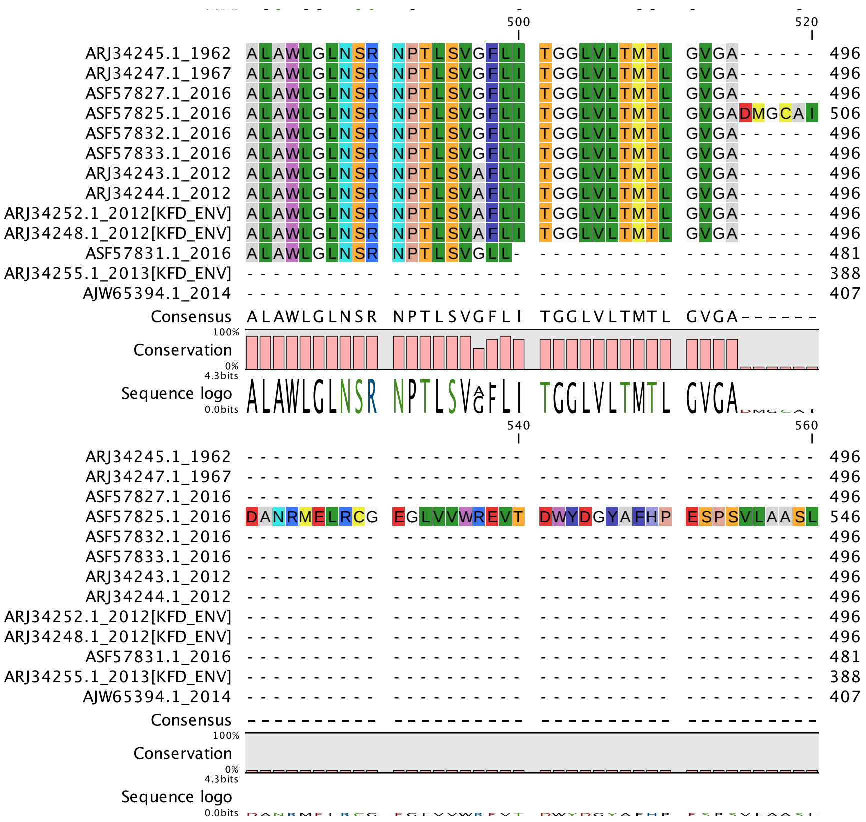
500
520
ARJ34245.1_1962 ALAWLGLNSR NPTLSVGFLI TGGLVLTMTL GVGA------ 496
ARJ34247.1_1967 ALAWLGLNSR NPTLSVGFLI TGGLVLTMTL GVGA------ 496
ASF57827.1_2016 ALAWLGLNSR NPTLSVGFLI TGGLVLTMTL GVGA------ 496
ASF57825.1_2016 ALAWLGLNSR NPTLSVGFLI TGGLVLTMTL GVGADMGCAI 506
ASF57832.1_2016 ALAWLGLNSR NPTLSVGFLI TGGLVLTMTL GVGA------ 496
ASF57833.1_2016 ALAWLGLNSR NPTLSVGFLI TGGLVLTMTL GVGA------ 496
ARJ34243.1_2012 ALAWLGLNSR NPTLSVAFLI TGGLVLTMTL GVGA------ 496
ARJ34244.1_2012 ALAWLGLNSR NPTLSVAFLI TGGLVLTMTL GVGA------ 496
ARJ34252.1_2012[KFD_ENV] ALAWLGLNSR NPTLSVAFLI TGGLVLTMTL GVGA------ 496
ARJ34248.1_2012[KFD_ENV] ALAWLGLNSR NPTLSVAFLI TGGLVLTMTL GVGA------ 496
ASF57831.1_2016 ALAWLGLNSR NPTLSVGLL- ---------- ---------- 481
ARJ34255.1_2013[KFD_ENV] ---------- ---------- ---------- ---------- 388
AJW65394.1_2014 ---------- ---------- ---------- ---------- 407
Consensus ALAWLGLNSR NPTLSVGFLI TGGLVLTMTL GVGA------
Conservation
100%
0%
Sequence logo
4.3bits
0.0bits
540
560
ARJ34245.1_1962 ---------- ---------- ---------- ---------- 496
ARJ34247.1_1967 ---------- ---------- ---------- ---------- 496
ASF57827.1_2016 ---------- ---------- ---------- ---------- 496
ASF57825.1_2016 DANRMELRCG EGLVVWREVT DWYDGYAFHP ESPSVLAASL 546
ASF57832.1_2016 ---------- ---------- ---------- ---------- 496
ASF57833.1_2016 ---------- ---------- ---------- ---------- 496
ARJ34243.1_2012 ---------- ---------- ---------- ---------- 496
ARJ34244.1_2012 ---------- ---------- ---------- ---------- 496
ARJ34252.1_2012[KFD_ENV] ---------- ---------- ---------- ---------- 496
ARJ34248.1_2012[KFD_ENV] ---------- ---------- ---------- ---------- 496
ASF57831.1_2016 ---------- ---------- ---------- ---------- 481
ARJ34255.1_2013[KFD_ENV] ---------- ---------- ---------- ---------- 388
AJW65394.1_2014 ---------- ---------- ---------- ---------- 407
Consensus ---------- ---------- ---------- ----------
Conservation
100%
0%
Sequence logo
4.3bits
0.0bits

Supplementary Table 2:

Conservation scores of KFDV envelope protein sequence

Amino acid Position	Conservation score
1	0.154
2	0.154
3	0.077
4	0.077
5	0.077
6	0.077
7	0.077
8	0.077
9	0.077
10	0.077
11	0.077
12	0.077
13	0.077
14	0.077
15	0.077
16	0.077
17	0.154
18	0.154
19	1
20	1
21	1
22	1
23	1
24	1
25	1
26	1
27	1
28	1
29	1
30	1
31	1
32	1
33	1
34	1
35	1
36	1
37	1
38	1
39	1
40	1

41	1
42	1
43	1
44	1
45	1
46	1
47	1
48	1
49	1
50	1
51	1
52	1
53	1
54	1
55	1
56	1
57	1
58	1
59	1
60	1
61	1
62	1
63	1
64	1
65	1
66	1
67	1
68	1
69	1
70	1
71	1
72	1
73	1
74	1
75	1
76	1
77	1
78	1
79	1
80	1
81	1
82	1
83	1
84	0.923
85	1

86	1
87	1
88	1
89	1
90	1
91	1
92	1
93	1
94	1
95	1
96	1
97	1
98	1
99	1
100	1
101	1
102	1
103	1
104	1
105	1
106	1
107	1
108	1
109	1
110	1
111	1
112	1
113	1
114	1
115	1
116	1
117	1
118	1
119	1
120	1
121	1
122	1
123	1
124	1
125	1
126	1
127	1
128	1
129	1
130	1

131	1
132	1
133	1
134	1
135	1
136	1
137	1
138	1
139	1
140	1
141	0.769
142	1
143	1
144	1
145	1
146	1
147	1
148	1
149	1
150	1
151	1
152	1
153	1
154	1
155	1
156	1
157	1
158	1
159	1
160	1
161	1
162	1
163	1
164	1
165	1
166	1
167	1
168	1
169	1
170	1
171	1
172	1
173	1
174	1
175	1

176	1
177	1
178	1
179	1
180	1
181	1
182	1
183	1
184	1
185	1
186	1
187	1
188	1
189	1
190	1
191	1
192	1
193	1
194	1
195	1
196	1
197	1
198	1
199	1
200	1
201	1
202	1
203	1
204	1
205	1
206	1
207	1
208	1
209	1
210	1
211	1
212	1
213	1
214	1
215	1
216	1
217	1
218	1
219	1
220	1

221	1
222	1
223	1
224	1
225	1
226	1
227	1
228	1
229	1
230	1
231	1
232	1
233	1
234	1
235	1
236	1
237	1
238	1
239	1
240	1
241	1
242	1
243	1
244	1
245	1
246	1
247	1
248	1
249	1
250	1
251	1
252	1
253	1
254	1
255	1
256	1
257	0.692
258	1
259	1
260	1
261	1
262	1
263	1
264	1
265	1

266	1
267	1
268	1
269	1
270	1
271	1
272	1
273	1
274	1
275	1
276	1
277	1
278	1
279	1
280	1
281	1
282	1
283	1
284	1
285	1
286	1
287	1
288	1
289	1
290	1
291	1
292	1
293	1
294	1
295	1
296	1
297	1
298	1
299	1
300	1
301	1
302	1
303	1
304	1
305	1
306	1
307	1
308	1
309	1
310	1

311	1
312	1
313	1
314	1
315	1
316	1
317	1
318	1
319	1
320	1
321	1
322	1
323	1
324	1
325	1
326	1
327	1
328	1
329	1
330	1
331	1
332	0.923
333	0.923
334	1
335	1
336	1
337	1
338	1
339	1
340	1
341	1
342	1
343	1
344	1
345	1
346	0.923
347	1
348	1
349	1
350	1
351	1
352	1
353	1
354	1
355	1

356	0.923
357	1
358	1
359	1
360	1
361	1
362	1
363	1
364	1
365	1
366	1
367	1
368	1
369	1
370	1
371	1
372	1
373	1
374	1
375	1
376	1
377	1
378	1
379	1
380	1
381	1
382	1
383	1
384	1
385	1
386	1
387	1
388	1
389	1
390	1
391	1
392	1
393	1
394	1
395	1
396	1
397	1
398	1
399	1
400	1

401	1
402	1
403	1
404	1
405	1
406	1
407	0.846
408	0.846
409	0.846
410	0.846
411	0.846
412	0.846
413	0.846
414	0.846
415	0.846
416	0.846
417	0.846
418	0.846
419	0.846
420	0.846
421	0.846
422	0.846
423	0.846
424	0.846
425	0.846
426	0.846
427	0.846
428	0.846
429	0.846
430	0.846
431	0.846
432	0.846
433	0.846
434	0.846
435	0.846
436	0.846
437	0.846
438	0.846
439	0.846
440	0.846
441	0.846
442	0.846
443	0.846
444	0.846
445	0.846

446	0.846
447	0.462
448	0.846
449	0.846
450	0.846
451	0.846
452	0.846
453	0.846
454	0.846
455	0.846
456	0.846
457	0.846
458	0.846
459	0.846
460	0.846
461	0.846
462	0.846
463	0.846
464	0.846
465	0.846
466	0.846
467	0.846
468	0.846
469	0.846
470	0.846
471	0.846
472	0.846
473	0.769
474	0.846
475	0.846
476	0.846
477	0.846
478	0.846
479	0.846
480	0.846
481	0.846
482	0.846
483	0.846
484	0.846
485	0.846
486	0.846
487	0.846
488	0.846
489	0.846
490	0.846

491	0.846
492	0.846
493	0.846
494	0.846
495	0.846
496	0.846
497	0.538
498	0.769
499	0.846
500	0.769
501	0.769
502	0.769
503	0.769
504	0.769
505	0.769
506	0.769
507	0.769
508	0.769
509	0.769
510	0.769
511	0.769
512	0.769
513	0.769
514	0.769
515	0.077
516	0.077
517	0.077
518	0.077
519	0.077
520	0.077
521	0.077
522	0.077
523	0.077
524	0.077
525	0.077
526	0.077
527	0.077
528	0.077
529	0.077
530	0.077
531	0.077
532	0.077
533	0.077
534	0.077
535	0.077

536	0.077
537	0.077
538	0.077
539	0.077
540	0.077
541	0.077
542	0.077
543	0.077
544	0.077
545	0.077
546	0.077
547	0.077
548	0.077
549	0.077
550	0.077
551	0.077
552	0.077
553	0.077
554	0.077
555	0.077
556	0.077
557	0.077
558	0.077
559	0.077
560	0.077

Supplementary Table 3:

List of epitopes obtained from IEDB-MHC-I tool.

Allele	length	peptide	Percentile Rank
HLA-B*57:01	9	KAWQVHRDW	0.06
HLA-A*30:02	9	KTILTLGDY	0.06
HLA-B*57:01	10	KTAEHLPKAW	0.09
HLA-A*11:01	9	STIGRVLEK	0.11
HLA-A*33:01	10	DYGDISLTCR	0.15
HLA-B*58:01	10	KTAEHLPKAW	0.17
HLA-B*07:02	10	KPCRIPVRAV	0.18
HLA-A*11:01	9	ASFTTQSEK	0.2
HLA-B*58:01	9	KAWQVHRDW	0.2
HLA-A*11:01	9	VTCDVGLEK	0.2
HLA-B*40:01	9	QEWNHADRL	0.24
HLA-A*68:01	10	MTYTVCEGSK	0.24
HLA-A*02:03	9	ILLKSLAGV	0.24
HLA-B*40:01	10	GEPNVNVASL	0.25
HLA-A*30:02	9	KVEPHTGDY	0.26
HLA-A*02:03	9	KMKGMTYTV	0.27
HLA-B*44:03	9	QEWNHADRL	0.29
HLA-A*26:01	9	YTVCEGSKF	0.29
HLA-A*03:01	9	ASFTTQSEK	0.33
HLA-A*02:06	9	ILLKSLAGV	0.33
HLA-A*02:03	10	SLTCRVTSGV	0.35
HLA-A*02:03	10	SLAGVPVANI	0.39
HLA-A*24:02	10	IYVGELSHQW	0.4
HLA-A*02:01	9	ILLKSLAGV	0.4
HLA-A*32:01	9	KMKGMTYTV	0.4
HLA-B*35:01	9	LPPGDNIIY	0.4
HLA-A*68:01	10	TASFTTQSEK	0.41
HLA-B*40:01	9	METTGGGFV	0.41
HLA-A*02:03	10	LKMKGMTYTV	0.42
HLA-A*11:01	10	TASFTTQSEK	0.44
HLA-A*03:01	10	MTYTVCEGSK	0.44
HLA-B*51:01	9	LAGVPVANI	0.44
HLA-A*23:01	10	TYTVCEGSKF	0.48
HLA-B*58:01	9	YVGELSHQW	0.5

Query results

Supplementary Table 4:
List of epitopes predicted by MAPPP tool.

Parameters	
Length of peptide sequence	
Calculated subsequence scores	
Max. number of results	4
Min. score	1
Matrices to get values	SYFPEITHI

Go to
HLA A1 - 8 mers, HLA A1 - 9 mers, HLA A1 - 10 mers,
HLA A3 - 8 mers, HLA A3 - 9 mers, HLA A3 - 10 mers,
HLA A24 - 8 mers, HLA A24 - 9 mers, HLA A24 - 10 mers,
HLA A26 - 8 mers, HLA A26 - 9 mers, HLA A26 - 10 mers,
HLA A68.1 - 8 mers, HLA A68.1 - 9 mers, HLA A68.1 - 10 mers,
HLA A 0201 - 8 mers, HLA A 0201 - 9 mers, HLA A 0201 - 10 mers,
HLA A 02 05 - 8 mers, HLA A 02 05 - 9 mers, HLA A 02 05 - 10 mers,
HLA A 1101 - 8 mers, HLA A 1101 - 9 mers, HLA A 1101 - 10 mers,
HLA A 3101 - 8 mers, HLA A 3101 - 9 mers, HLA A 3101 - 10 mers,
HLA A 3302 - 8 mers, HLA A 3302 - 9 mers, HLA A 3302 - 10 mers,
HLA B14 - 8 mers, HLA B14 - 9 mers, HLA B14 - 10 mers,
HLA B40 - 8 mers, HLA B40 - 9 mers, HLA B40 - 10 mers,
HLA B60 - 8 mers, HLA B60 - 9 mers, HLA B60 - 10 mers,
HLA B61 - 8 mers, HLA B61 - 9 mers, HLA B61 - 10 mers,
HLA B62 - 8 mers, HLA B62 - 9 mers, HLA B62 - 10 mers,
HLA B7 - 8 mers, HLA B7 - 9 mers, HLA B7 - 10 mers,
HLA B8 - 8 mers, HLA B8 - 9 mers, HLA B8 - 10 mers,
HLA B 0702 - 8 mers, HLA B 0702 - 9 mers, HLA B 0702 - 10 mers,
HLA B 1510 - 8 mers, HLA B 1510 - 9 mers, HLA B 1510 - 10 mers,
HLA B 2702 - 8 mers, HLA B 2702 - 9 mers, HLA B 2702 - 10 mers,
HLA B 2705 - 8 mers, HLA B 2705 - 9 mers, HLA B 2705 - 10 mers,
HLA B 3501 - 8 mers, HLA B 3501 - 9 mers, HLA B 3501 - 10 mers,
HLA B 3701 - 8 mers, HLA B 3701 - 9 mers, HLA B 3701 - 10 mers,
HLA B 3801 - 8 mers, HLA B 3801 - 9 mers, HLA B 3801 - 10 mers,
HLA B 3 901 - 8 mers, HLA B 3 901 - 9 mers, HLA B 3 901 - 10 mers,
HLA B 4403 - 8 mers, HLA B 4403 - 9 mers, HLA B 4403 - 10 mers,
HLA B 5101 - 8 mers, HLA B 5101 - 9 mers, HLA B 5101 - 10 mers,
HLA B 5102 - 8 mers, HLA B 5102 - 9 mers, HLA B 5102 - 10 mers,
HLA B 5103 - 8 mers, HLA B 5103 - 9 mers, HLA B 5103 - 10 mers,
HLA B 5201 - 8 mers, HLA B 5201 - 9 mers, HLA B 5201 - 10 mers,
HLA B 5 801 - 8 mers, HLA B 5 801 - 9 mers, HLA B 5 801 - 10 mers,
HLA Cw 0301 - 8 mers, HLA Cw 0301 - 9 mers, HLA Cw 0301 - 10 mers,
HLA Cw 0401 - 8 mers, HLA Cw 0401 - 9 mers, HLA Cw 0401 - 10 mers,
HLA Cw 0602 - 8 mers, HLA Cw 0602 - 9 mers, HLA Cw 0602 - 10 mers,
HLA Cw 0702 - 8 mers, HLA Cw 0702 - 9 mers, HLA Cw 0702 - 10 mers,
H2 Db - 8 mers, H2 Db - 9 mers, H2 Db - 10 mers,
H2 Db revised - 8 mers, H2 Db revised - 9 mers, H2 Db revised - 10 mers,
H2 Dd - 8 mers, H2 Dd - 9 mers, H2 Dd - 10 mers,
H2 Kb - 8 mers, H2 Kb - 9 mers, H2 Kb - 10 mers,
H2 Kd - 8 mers, H2 Kd - 9 mers, H2 Kd - 10 mers,
H2 KD - 8 mers, H2 KD - 9 mers, H2 KD - 10 mers,
H2 Kk - 8 mers, H2 Kk - 9 mers, H2 Kk - 10 mers,
H2 Ld - 8 mers, H2 Ld - 9 mers, H2 Ld - 10 mers,
Cattle A20 - 8 mers, Cattle A20 - 9 mers, Cattle A20 - 10 mers,

HLA A1 - 8 mers
No corresponding coefficient table could be found

HLA A1 - 9 mers				
Max. score that could've been reached using this molecule type				50
**Rank**	**Start position**	**Sequence**	**% of max. score**	**Score**
1	66	DTVVMEVTY	42 %	21
2	118	LPPGDNIIY	42 %	21
3	15	VANIEGSKY	38 %	19
4	60	PTDSGHDTV	36 %	18

HLA A1 - 10 mers				
Max. score that could've been reached using this molecule type				50
**Rank**	**Start position**	**Sequence**	**% of max. score**	**Score**
1	66	DTVVMEVTYT	42 %	21
2	118	LPPGDNIIYV	42 %	21
3	15	VANIEGSKYH	38 %	19
4	60	PTDSGHDTVV	36 %	18

HLA A3 - 8 mers
No corresponding coefficient table could be found

HLA A3 - 9 mers				
Max. score that could've been reached using this molecule type				43
**Rank**	**Start position**	**Sequence**	**% of max. score**	**Score**
1	38	KLKMKGMTY	67.44 %	29
2	81	RIPVRAVAH	60.46 %	26
3	14	PVANIEGSK	58.13 %	25
4	124	IIYVGELSH	58.13 %	25

HLA A3 - 10 mers				
Max. score that could've been reached using this molecule type				43
**Rank**	**Start position**	**Sequence**	**% of max. score**	**Score**
1	38	KLKMKGMTYT	67.44 %	29
2	81	RIPVRAVAHG	60.46 %	26
3	14	PVANIEGSKY	58.13 %	25
4	124	IIYVGELSHQ	58.13 %	25

HLA A24 - 8 mers
No corresponding coefficient table could be found

HLA A24 - 9 mers
No corresponding coefficient table could be found

HLA A24 - 10 mers
No corresponding coefficient table could be found

HLA A26 - 8 mers
No corresponding coefficient table could be found

HLA A26 - 9 mers
No corresponding coefficient table could be found

HLA A26 - 10 mers
No corresponding coefficient table could be found

HLA A68.1 - 8 mers
No corresponding coefficient table could be found

HLA A68.1 - 9 mers
No corresponding coefficient table could be found

HLA A68.1 - 10 mers
No corresponding coefficient table could be found

HLA A 0201 - 8 mers
No corresponding coefficient table could be found

HLA A 0201 - 9 mers				
Max. score that could've been reached using this molecule type				36
**Rank**	**Start position**	**Sequence**	**% of max. score**	**Score**
1	5	ILLKSLAGV	86.11 %	31
2	40	KMKGMTYTV	69.44 %	25
3	117	QLPPGDNII	63.88 %	23
4	98	SLITPNPSM	61.11 %	22

HLA A 0201 - 10 mers				
Max. score that could've been reached using this molecule type				36
**Rank**	**Start position**	**Sequence**	**% of max. score**	**Score**
1	5	ILLKSLAGVP	86.11 %	31
2	40	KMKGMTYTVC	69.44 %	25
3	117	QLPPGDNIIY	63.88 %	23
4	98	SLITPNPSME	61.11 %	22

HLA A 02 05 - 8 mers
No corresponding coefficient table could be found

HLA A 02 05 - 9 mers
No corresponding coefficient table could be found

HLA A 02 05 - 10 mers
No corresponding coefficient table could be found

HLA A 1101 - 8 mers
No corresponding coefficient table could be found

HLA A 1101 - 9 mers
No corresponding coefficient table could be found

HLA A 1101 - 10 mers
No corresponding coefficient table could be found

HLA A 3101 - 8 mers
No corresponding coefficient table could be found

HLA A 3101 - 9 mers
No corresponding coefficient table could be found

HLA A 3101 - 10 mers
No corresponding coefficient table could be found

HLA A 3302 - 8 mers
No corresponding coefficient table could be found

HLA A 3302 - 9 mers
No corresponding coefficient table could be found

HLA A 3302 - 10 mers
No corresponding coefficient table could be found

HLA B14 - 8 mers
No corresponding coefficient table could be found

HLA B14 - 9 mers
No corresponding coefficient table could be found

HLA B14 - 10 mers
No corresponding coefficient table could be found

HLA B40 - 8 mers
No corresponding coefficient table could be found

HLA B40 - 9 mers
No corresponding coefficient table could be found

HLA B40 - 10 mers
No corresponding coefficient table could be found

HLA B60 - 8 mers
No corresponding coefficient table could be found

HLA B60 - 9 mers
No corresponding coefficient table could be found

HLA B60 - 10 mers
No corresponding coefficient table could be found

HLA B61 - 8 mers
No corresponding coefficient table could be found

HLA B61 - 9 mers
No corresponding coefficient table could be found

HLA B61 - 10 mers
No corresponding coefficient table could be found

HLA B62 - 8 mers
No corresponding coefficient table could be found

HLA B62 - 9 mers
No corresponding coefficient table could be found

HLA B62 - 10 mers
No corresponding coefficient table could be found

HLA B7 - 8 mers
No corresponding coefficient table could be found

HLA B7 - 9 mers
No corresponding coefficient table could be found

HLA B7 - 10 mers
No corresponding coefficient table could be found

HLA B8 - 8 mers				
Max. score that could've been reached using this molecule type				40
**Rank**	**Start position**	**Sequence**	**% of max. score**	**Score**
1	38	KLKMKGMT	65 %	26
2	18	IEGSKYHL	52.5 %	21
3	36	LEKLKMKG	52.5 %	21
4	4	GILLKSLA	40 %	16

HLA B8 - 9 mers				
Max. score that could've been reached using this molecule type				43
**Rank**	**Start position**	**Sequence**	**% of max. score**	**Score**
1	38	KLKMKGMTY	60.46 %	26
2	36	LEKLKMKGM	51.16 %	22
3	91	EPNVNVASL	46.51 %	20
4	4	GILLKSLAG	39.53 %	17

HLA B8 - 10 mers				
Max. score that could've been reached using this molecule type				43
**Rank**	**Start position**	**Sequence**	**% of max. score**	**Score**
1	38	KLKMKGMTYT	60.46 %	26
2	36	LEKLKMKGMT	51.16 %	22
3	91	EPNVNVASLI	46.51 %	20
4	4	GILLKSLAGV	39.53 %	17

HLA B 0702 - 8 mers
No corresponding coefficient table could be found

HLA B 0702 - 9 mers				
Max. score that could've been reached using this molecule type				35
**Rank**	**Start position**	**Sequence**	**% of max. score**	**Score**
1	91	EPNVNVASL	65.71 %	23
2	78	KPCRIPVRA	54.28 %	19
3	59	PPTDSGHDT	48.57 %	17
4	101	TPNPSMETT	48.57 %	17

HLA B 0702 - 10 mers	
Max. score that could've been reached using this molecule type	35

Rank	Start position	Sequence	% of max. score	Score
1	91	EPNVNVASLI	65.71 %	23
2	78	KPCRIPVRAV	54.28 %	19
3	59	PPTDSGHDTV	48.57 %	17
4	101	TPNPSMETTG	48.57 %	17

**HLA B 1510 - 8 mers**
No corresponding coefficient table could be found

**HLA B 1510 - 9 mers**				
Max. score that could've been reached using this molecule type				31
**Rank**	**Start position**	**Sequence**	**% of max. score**	**Score**
1	28	GHVTCDVGL	74.19 %	23
2	108	TTGGGFVEL	51.61 %	16
3	137	KGSTIGRVL	48.38 %	15
4	23	YHLQSGHVT	45.16 %	14

**HLA B 1510 - 10 mers**				
Max. score that could've been reached using this molecule type				31
**Rank**	**Start position**	**Sequence**	**% of max. score**	**Score**
1	28	GHVTCDVGLE	74.19 %	23
2	108	TTGGGFVELQ	51.61 %	16
3	137	KGSTIGRVLE	48.38 %	15
4	23	YHLQSGHVTC	45.16 %	14

**HLA B 2702 - 8 mers**
No corresponding coefficient table could be found

**HLA B 2702 - 9 mers**
No corresponding coefficient table could be found

**HLA B 2702 - 10 mers**
No corresponding coefficient table could be found

**HLA B 2705 - 8 mers**
No corresponding coefficient table could be found

**HLA B 2705 - 9 mers**				
Max. score that could've been reached using this molecule type				37
**Rank**	**Start position**	**Sequence**	**% of max. score**	**Score**
1	57	KRPPTDSGH	62.16 %	23
2	0	GDQTGILLK	51.35 %	19
3	139	STIGRVLEK	45.94 %	17
4	28	GHVTCDVGL	43.24 %	16

**HLA B 2705 - 10 mers**				
Max. score that could've been reached using this molecule type				37
**Rank**	**Start position**	**Sequence**	**% of max. score**	**Score**
1	57	KRPPTDSGHD	62.16 %	23
2	0	GDQTGILLKS	51.35 %	19
3	139	STIGRVLEKT	45.94 %	17
4	28	GHVTCDVGLE	43.24 %	16

**HLA B 3501 - 8 mers**
No corresponding coefficient table could be found

**HLA B 3501 - 9 mers**
No corresponding coefficient table could be found

**HLA B 3501 - 10 mers**
No corresponding coefficient table could be found

**HLA B 3701 - 8 mers**
No corresponding coefficient table could be found

HLA B 3701 - 9 mers
No corresponding coefficient table could be found

HLA B 3701 - 10 mers
No corresponding coefficient table could be found

HLA B 3801 - 8 mers
No corresponding coefficient table could be found

HLA B 3801 - 9 mers
No corresponding coefficient table could be found

HLA B 3801 - 10 mers
No corresponding coefficient table could be found

HLA B 3 901 - 8 mers
No corresponding coefficient table could be found

HLA B 3 901 - 9 mers
No corresponding coefficient table could be found

HLA B 3 901 - 10 mers
No corresponding coefficient table could be found

HLA B 4403 - 8 mers
No corresponding coefficient table could be found

HLA B 4403 - 9 mers
No corresponding coefficient table could be found

HLA B 4403 - 10 mers
No corresponding coefficient table could be found

HLA B 5101 - 8 mers
No corresponding coefficient table could be found

HLA B 5101 - 9 mers
No corresponding coefficient table could be found

HLA B 5101 - 10 mers
No corresponding coefficient table could be found

HLA B 5102 - 8 mers
No corresponding coefficient table could be found

HLA B 5102 - 9 mers
No corresponding coefficient table could be found

HLA B 5102 - 10 mers
No corresponding coefficient table could be found

HLA B 5103 - 8 mers
No corresponding coefficient table could be found

HLA B 5103 - 9 mers
No corresponding coefficient table could be found

HLA B 5103 - 10 mers
No corresponding coefficient table could be found

HLA B 5201 - 8 mers
No corresponding coefficient table could be found

HLA B 5201 - 9 mers
No corresponding coefficient table could be found

HLA B 5201 - 10 mers
No corresponding coefficient table could be found

HLA B 5 801 - 8 mers
No corresponding coefficient table could be found

HLA B 5 801 - 9 mers
No corresponding coefficient table could be found

HLA B 5 801 - 10 mers
No corresponding coefficient table could be found

HLA Cw 0301 - 8 mers
No corresponding coefficient table could be found

HLA Cw 0301 - 9 mers
No corresponding coefficient table could be found

HLA Cw 0301 - 10 mers
No corresponding coefficient table could be found

HLA Cw 0401 - 8 mers
No corresponding coefficient table could be found

HLA Cw 0401 - 9 mers
No corresponding coefficient table could be found

HLA Cw 0401 - 10 mers
No corresponding coefficient table could be found

HLA Cw 0602 - 8 mers
No corresponding coefficient table could be found

HLA Cw 0602 - 9 mers
No corresponding coefficient table could be found

HLA Cw 0602 - 10 mers
No corresponding coefficient table could be found

HLA Cw 0702 - 8 mers
No corresponding coefficient table could be found

HLA Cw 0702 - 9 mers
No corresponding coefficient table could be found

HLA Cw 0702 - 10 mers
No corresponding coefficient table could be found

H2 Db - 8 mers
No corresponding coefficient table could be found

H2 Db - 9 mers				
Max. score that could've been reached using this molecule type				35
**Rank**	**Start position**	**Sequence**	**% of max. score**	**Score**
1	91	EPNVNVASL	62.85 %	22
2	119	PPGDNIIYV	54.28 %	19
3	31	TCDVGLEKL	48.57 %	17
4	10	LAGVPVANI	45.71 %	16

H2 Db - 10 mers	
Max. score that could've been reached using this molecule type	35

Rank	Start position	Sequence	% of max. score	Score
1	91	EPNVNVASLI	62.85 %	22
2	119	PPGDNIIYVG	54.28 %	19
3	31	TCDVGLEKLK	48.57 %	17
4	10	LAGVPVANIE	45.71 %	16

H2 Db revised - 8 mers
No corresponding coefficient table could be found

H2 Db revised - 9 mers
No corresponding coefficient table could be found

H2 Db revised - 10 mers
No corresponding coefficient table could be found

H2 Dd - 8 mers
No corresponding coefficient table could be found

H2 Dd - 9 mers
No corresponding coefficient table could be found

H2 Dd - 10 mers
No corresponding coefficient table could be found

H2 Kb - 8 mers				
Max. score that could've been reached using this molecule type				31
Rank	Start position	Sequence	% of max. score	Score
1	109	TGGGFVEL	67.74 %	21
2	3	TGILLKSL	38.70 %	12
3	19	EGSKYHLQ	38.70 %	12
4	32	CDVGLEKL	38.70 %	12

H2 Kb - 9 mers
No corresponding coefficient table could be found

H2 Kb - 10 mers
No corresponding coefficient table could be found

H2 Kd - 8 mers
No corresponding coefficient table could be found

H2 Kd - 9 mers				
Max. score that could've been reached using this molecule type				38
Rank	Start position	Sequence	% of max. score	Score
1	22	KYHLQSGHV	47.36 %	18
2	73	TYTGSKPCR	47.36 %	18
3	92	PNVNVASLI	47.36 %	18
4	116	LQLPPGDNI	47.36 %	18

H2 Kd - 10 mers				
Max. score that could've been reached using this molecule type				38
Rank	Start position	Sequence	% of max. score	Score
1	22	KYHLQSGHVT	47.36 %	18
2	73	TYTGSKPCRI	47.36 %	18
3	92	PNVNVASLIT	47.36 %	18
4	116	LQLPPGDNII	47.36 %	18

H2 KD - 8 mers
No corresponding coefficient table could be found

H2 KD - 9 mers
No corresponding coefficient table could be found

H2 KD - 10 mers

No corresponding coefficient table could be found

H2 Kk - 8 mers				
Max. score that could've been reached using this molecule type				30
**Rank**	**Start position**	**Sequence**	**% of max. score**	**Score**
1	61	TDSGHDTV	63.33 %	19
2	65	HDTVVMEV	60 %	18
3	11	AGVPVANI	46.66 %	14
4	106	METTGGGF	46.66 %	14

H2 Kk - 9 mers				
Max. score that could've been reached using this molecule type				30
**Rank**	**Start position**	**Sequence**	**% of max. score**	**Score**
1	106	METTGGGFV	73.33 %	22
2	61	TDSGHDTVV	60 %	18
3	116	LQLPPGDNI	53.33 %	16
4	10	LAGVPVANI	46.66 %	14

H2 Kk - 10 mers				
Max. score that could've been reached using this molecule type				30
**Rank**	**Start position**	**Sequence**	**% of max. score**	**Score**
1	106	METTGGGFVE	73.33 %	22
2	61	TDSGHDTVVM	60 %	18
3	116	LQLPPGDNII	53.33 %	16
4	10	LAGVPVANIE	46.66 %	14

H2 Ld - 8 mers
No corresponding coefficient table could be found

H2 Ld - 9 mers				
Max. score that could've been reached using this molecule type				31
**Rank**	**Start position**	**Sequence**	**% of max. score**	**Score**
1	91	EPNVNVASL	67.74 %	21
2	62	DSGHDTVVM	54.83 %	17
3	108	TTGGGFVEL	48.38 %	15
4	31	TCDVGLEKL	45.16 %	14

H2 Ld - 10 mers				
Max. score that could've been reached using this molecule type				31
**Rank**	**Start position**	**Sequence**	**% of max. score**	**Score**
1	91	EPNVNVASLI	67.74 %	21
2	62	DSGHDTVVME	54.83 %	17
3	108	TTGGGFVELQ	48.38 %	15
4	31	TCDVGLEKLK	45.16 %	14

Cattle A20 - 8 mers
No corresponding coefficient table could be found

Cattle A20 - 9 mers
No corresponding coefficient table could be found

Cattle A20 - 10 mers
No corresponding coefficient table could be found

The protein sequence was :

```
GDQTGILLKSLAGVPVANIEGSKYHLQSGHVTCDVGLEKLKMKGMTYTVCEGSKFAWKRPPTDSGHDTVVMEVTYTGSKPCRIPVRAVAHGEPNVNVASLITPNPSMETTGG
```

Query results

Parameters	
Length of peptide sequence	
Calculated subsequence scores	
Max. number of results	4
Min. score	1
Matrices to get values	SYFPEITHI

Go to
HLA A1 - 8 mers, HLA A1 - 9 mers, HLA A1 - 10 mers,
HLA A3 - 8 mers, HLA A3 - 9 mers, HLA A3 - 10 mers,
HLA A24 - 8 mers, HLA A24 - 9 mers, HLA A24 - 10 mers,
HLA A26 - 8 mers, HLA A26 - 9 mers, HLA A26 - 10 mers,
HLA A68.1 - 8 mers, HLA A68.1 - 9 mers, HLA A68.1 - 10 mers,
HLA A 0201 - 8 mers, HLA A 0201 - 9 mers, HLA A 0201 - 10 mers,
HLA A 02 05 - 8 mers, HLA A 02 05 - 9 mers, HLA A 02 05 - 10 mers,
HLA A 1101 - 8 mers, HLA A 1101 - 9 mers, HLA A 1101 - 10 mers,
HLA A 3101 - 8 mers, HLA A 3101 - 9 mers, HLA A 3101 - 10 mers,
HLA A 3302 - 8 mers, HLA A 3302 - 9 mers, HLA A 3302 - 10 mers,
HLA B14 - 8 mers, HLA B14 - 9 mers, HLA B14 - 10 mers,
HLA B40 - 8 mers, HLA B40 - 9 mers, HLA B40 - 10 mers,
HLA B60 - 8 mers, HLA B60 - 9 mers, HLA B60 - 10 mers,
HLA B61 - 8 mers, HLA B61 - 9 mers, HLA B61 - 10 mers,
HLA B62 - 8 mers, HLA B62 - 9 mers, HLA B62 - 10 mers,
HLA B7 - 8 mers, HLA B7 - 9 mers, HLA B7 - 10 mers,
HLA B8 - 8 mers, HLA B8 - 9 mers, HLA B8 - 10 mers,
HLA B 0702 - 8 mers, HLA B 0702 - 9 mers, HLA B 0702 - 10 mers,
HLA B 1510 - 8 mers, HLA B 1510 - 9 mers, HLA B 1510 - 10 mers,
HLA B 2702 - 8 mers, HLA B 2702 - 9 mers, HLA B 2702 - 10 mers,
HLA B 2705 - 8 mers, HLA B 2705 - 9 mers, HLA B 2705 - 10 mers,
HLA B 3501 - 8 mers, HLA B 3501 - 9 mers, HLA B 3501 - 10 mers,
HLA B 3701 - 8 mers, HLA B 3701 - 9 mers, HLA B 3701 - 10 mers,
HLA B 3801 - 8 mers, HLA B 3801 - 9 mers, HLA B 3801 - 10 mers,
HLA B 3 901 - 8 mers, HLA B 3 901 - 9 mers, HLA B 3 901 - 10 mers,
HLA B 4403 - 8 mers, HLA B 4403 - 9 mers, HLA B 4403 - 10 mers,
HLA B 5101 - 8 mers, HLA B 5101 - 9 mers, HLA B 5101 - 10 mers,
HLA B 5102 - 8 mers, HLA B 5102 - 9 mers, HLA B 5102 - 10 mers,
HLA B 5103 - 8 mers, HLA B 5103 - 9 mers, HLA B 5103 - 10 mers,
HLA B 5201 - 8 mers, HLA B 5201 - 9 mers, HLA B 5201 - 10 mers,
HLA B 5 801 - 8 mers, HLA B 5 801 - 9 mers, HLA B 5 801 - 10 mers,
HLA Cw 0301 - 8 mers, HLA Cw 0301 - 9 mers, HLA Cw 0301 - 10 mers,
HLA Cw 0401 - 8 mers, HLA Cw 0401 - 9 mers, HLA Cw 0401 - 10 mers,
HLA Cw 0602 - 8 mers, HLA Cw 0602 - 9 mers, HLA Cw 0602 - 10 mers,
HLA Cw 0702 - 8 mers, HLA Cw 0702 - 9 mers, HLA Cw 0702 - 10 mers,
H2 Db - 8 mers, H2 Db - 9 mers, H2 Db - 10 mers,
H2 Db revised - 8 mers, H2 Db revised - 9 mers, H2 Db revised - 10 mers,
H2 Dd - 8 mers, H2 Dd - 9 mers, H2 Dd - 10 mers,
H2 Kb - 8 mers, H2 Kb - 9 mers, H2 Kb - 10 mers,
H2 Kd - 8 mers, H2 Kd - 9 mers, H2 Kd - 10 mers,
H2 KD - 8 mers, H2 KD - 9 mers, H2 KD - 10 mers,
H2 Kk - 8 mers, H2 Kk - 9 mers, H2 Kk - 10 mers,
H2 Ld - 8 mers, H2 Ld - 9 mers, H2 Ld - 10 mers,
Cattle A20 - 8 mers, Cattle A20 - 9 mers, Cattle A20 - 10 mers,

HLA A1 - 8 mers
No corresponding coefficient table could be found

HLA A1 - 9 mers				
Max. score that could've been reached using this molecule type				50
**Rank**	**Start position**	**Sequence**	**% of max. score**	**Score**
1	0	KVEPHTGDY	58 %	29
2	29	KTILTLGDY	42 %	21
3	26	QSEKTILTL	40 %	20
4	102	FGEPHAVKM	34 %	17

HLA A1 - 10 mers				
Max. score that could've been reached using this molecule type				50
**Rank**	**Start position**	**Sequence**	**% of max. score**	**Score**
1	0	KVEPHTGDYL	58 %	29
2	29	KTILTLGDYG	42 %	21
3	26	QSEKTILTLG	40 %	20
4	102	FGEPHAVKMD	34 %	17

HLA A3 - 8 mers
No corresponding coefficient table could be found

HLA A3 - 9 mers				
Max. score that could've been reached using this molecule type				43
**Rank**	**Start position**	**Sequence**	**% of max. score**	**Score**
1	0	KVEPHTGDY	53.48 %	23
2	98	RLVEFGEPH	51.16 %	22
3	66	HLPKAWQVH	44.18 %	19
4	49	GVDPAQTVV	41.86 %	18

HLA A3 - 10 mers				
Max. score that could've been reached using this molecule type				43
**Rank**	**Start position**	**Sequence**	**% of max. score**	**Score**
1	0	KVEPHTGDYL	53.48 %	23
2	98	RLVEFGEPHA	51.16 %	22
3	66	HLPKAWQVHR	44.18 %	19
4	49	GVDPAQTVVL	41.86 %	18

HLA A24 - 8 mers
No corresponding coefficient table could be found

HLA A24 - 9 mers
No corresponding coefficient table could be found

HLA A24 - 10 mers
No corresponding coefficient table could be found

HLA A26 - 8 mers
No corresponding coefficient table could be found

HLA A26 - 9 mers
No corresponding coefficient table could be found

HLA A26 - 10 mers
No corresponding coefficient table could be found

HLA A68.1 - 8 mers
No corresponding coefficient table could be found

HLA A68.1 - 9 mers
No corresponding coefficient table could be found

HLA A68.1 - 10 mers
No corresponding coefficient table could be found

HLA A 0201 - 8 mers
No corresponding coefficient table could be found

HLA A 0201 - 9 mers				
Max. score that could've been reached using this molecule type				36
**Rank**	**Start position**	**Sequence**	**% of max. score**	**Score**
1	62	KTAEHLPKA	55.55 %	20
2	42	LTCRVTSGV	52.77 %	19
3	32	LTLGDYGDI	50 %	18
4	52	PAQTVVLEL	50 %	18

HLA A 0201 - 10 mers				
Max. score that could've been reached using this molecule type				36
**Rank**	**Start position**	**Sequence**	**% of max. score**	**Score**
1	62	KTAEHLPKAW	55.55 %	20
2	42	LTCRVTSGVD	52.77 %	19
3	32	LTLGDYGDIS	50 %	18
4	52	PAQTVVLELD	50 %	18

HLA A 02 05 - 8 mers
No corresponding coefficient table could be found

HLA A 02 05 - 9 mers
No corresponding coefficient table could be found

HLA A 02 05 - 10 mers
No corresponding coefficient table could be found

HLA A 1101 - 8 mers
No corresponding coefficient table could be found

HLA A 1101 - 9 mers
No corresponding coefficient table could be found

HLA A 1101 - 10 mers
No corresponding coefficient table could be found

HLA A 3101 - 8 mers
No corresponding coefficient table could be found

HLA A 3101 - 9 mers
No corresponding coefficient table could be found

HLA A 3101 - 10 mers
No corresponding coefficient table could be found

HLA A 3302 - 8 mers
No corresponding coefficient table could be found

HLA A 3302 - 9 mers
No corresponding coefficient table could be found

HLA A 3302 - 10 mers
No corresponding coefficient table could be found

HLA B14 - 8 mers
No corresponding coefficient table could be found

HLA B14 - 9 mers
No corresponding coefficient table could be found

HLA B14 - 10 mers
No corresponding coefficient table could be found

HLA B40 - 8 mers
No corresponding coefficient table could be found

HLA B40 - 9 mers
No corresponding coefficient table could be found

HLA B40 - 10 mers
No corresponding coefficient table could be found

HLA B60 - 8 mers
No corresponding coefficient table could be found

HLA B60 - 9 mers
No corresponding coefficient table could be found

HLA B60 - 10 mers
No corresponding coefficient table could be found

HLA B61 - 8 mers
No corresponding coefficient table could be found

HLA B61 - 9 mers
No corresponding coefficient table could be found

HLA B61 - 10 mers
No corresponding coefficient table could be found

HLA B62 - 8 mers
No corresponding coefficient table could be found

HLA B62 - 9 mers
No corresponding coefficient table could be found

HLA B62 - 10 mers
No corresponding coefficient table could be found

HLA B7 - 8 mers
No corresponding coefficient table could be found

HLA B7 - 9 mers
No corresponding coefficient table could be found

HLA B7 - 10 mers
No corresponding coefficient table could be found

HLA B8 - 8 mers				
Max. score that could've been reached using this molecule type				40
**Rank**	**Start position**	**Sequence**	**% of max. score**	**Score**
1	25	TQSEKTIL	55 %	22
2	27	SEKTILTL	55 %	22
3	60	LDKTAEHL	50 %	20
4	2	EPHTGDYL	45 %	18

HLA B8 - 9 mers				
Max. score that could've been reached using this molecule type				43
**Rank**	**Start position**	**Sequence**	**% of max. score**	**Score**
1	59	ELDKTAEHL	41.86 %	18
2	15	HSNRKTASF	39.53 %	17
3	41	SLTCRVTSG	39.53 %	17
4	67	LPKAWQVHR	39.53 %	17

HLA B8 - 10 mers				
Max. score that could've been reached using this molecule type				43
**Rank**	**Start position**	**Sequence**	**% of max. score**	**Score**
1	59	ELDKTAEHLP	41.86 %	18
2	15	HSNRKTASFT	39.53 %	17
3	41	SLTCRVTSGV	39.53 %	17
4	67	LPKAWQVHRD	39.53 %	17

HLA B 0702 - 8 mers
No corresponding coefficient table could be found

HLA B 0702 - 9 mers				
Max. score that could've been reached using this molecule type				35
**Rank**	**Start position**	**Sequence**	**% of max. score**	**Score**
1	104	EPHAVKMDI	51.42 %	18
2	2	EPHTGDYLA	48.57 %	17
3	50	VDPAQTVVL	42.85 %	15
4	73	VHRDWFEDL	42.85 %	15

HLA B 0702 - 10 mers	
Max. score that could've been reached using this molecule type	35

Rank	Start position	Sequence	% of max. score	Score
1	104	EPHAVKMDIF	51.42 %	18
2	2	EPHTGDYLAA	48.57 %	17
3	50	VDPAQTVVLE	42.85 %	15
4	73	VHRDWFEDLS	42.85 %	15

HLA B 1510 - 8 mers
No corresponding coefficient table could be found

HLA B 1510 - 9 mers				
Max. score that could've been reached using this molecule type				31
Rank	Start position	Sequence	% of max. score	Score
1	73	VHRDWFEDL	67.74 %	21
2	94	NHADRLVEF	67.74 %	21
3	105	PHAVKMDIF	61.29 %	19
4	26	QSEKTILTL	45.16 %	14

HLA B 1510 - 10 mers				
Max. score that could've been reached using this molecule type				31
Rank	Start position	Sequence	% of max. score	Score
1	73	VHRDWFEDLS	67.74 %	21
2	94	NHADRLVEFG	67.74 %	21
3	105	PHAVKMDIFN	61.29 %	19
4	26	QSEKTILTLG	45.16 %	14

HLA B 2702 - 8 mers
No corresponding coefficient table could be found

HLA B 2702 - 9 mers
No corresponding coefficient table could be found

HLA B 2702 - 10 mers
No corresponding coefficient table could be found

HLA B 2705 - 8 mers
No corresponding coefficient table could be found

HLA B 2705 - 9 mers				
Max. score that could've been reached using this molecule type				37
Rank	Start position	Sequence	% of max. score	Score
1	21	ASFTTQSEK	48.64 %	18
2	75	RDWFEDLSL	48.64 %	18
3	26	QSEKTILTL	45.94 %	17
4	10	AANESHSNR	43.24 %	16

HLA B 2705 - 10 mers				
Max. score that could've been reached using this molecule type				37
Rank	Start position	Sequence	% of max. score	Score
1	21	ASFTTQSEKT	48.64 %	18
2	75	RDWFEDLSLP	48.64 %	18
3	26	QSEKTILTLG	45.94 %	17
4	10	AANESHSNRK	43.24 %	16

HLA B 3501 - 8 mers
No corresponding coefficient table could be found

HLA B 3501 - 9 mers
No corresponding coefficient table could be found

HLA B 3501 - 10 mers
No corresponding coefficient table could be found

HLA B 3701 - 8 mers
No corresponding coefficient table could be found

HLA B 3701 - 9 mers
No corresponding coefficient table could be found

HLA B 3701 - 10 mers
No corresponding coefficient table could be found

HLA B 3801 - 8 mers
No corresponding coefficient table could be found

HLA B 3801 - 9 mers
No corresponding coefficient table could be found

HLA B 3801 - 10 mers
No corresponding coefficient table could be found

HLA B 3 901 - 8 mers
No corresponding coefficient table could be found

HLA B 3 901 - 9 mers
No corresponding coefficient table could be found

HLA B 3 901 - 10 mers
No corresponding coefficient table could be found

HLA B 4403 - 8 mers
No corresponding coefficient table could be found

HLA B 4403 - 9 mers
No corresponding coefficient table could be found

HLA B 4403 - 10 mers
No corresponding coefficient table could be found

HLA B 5101 - 8 mers
No corresponding coefficient table could be found

HLA B 5101 - 9 mers
No corresponding coefficient table could be found

HLA B 5101 - 10 mers
No corresponding coefficient table could be found

HLA B 5102 - 8 mers
No corresponding coefficient table could be found

HLA B 5102 - 9 mers
No corresponding coefficient table could be found

HLA B 5102 - 10 mers
No corresponding coefficient table could be found

HLA B 5103 - 8 mers
No corresponding coefficient table could be found

HLA B 5103 - 9 mers
No corresponding coefficient table could be found

HLA B 5103 - 10 mers
No corresponding coefficient table could be found

HLA B 5201 - 8 mers
No corresponding coefficient table could be found

HLA B 5201 - 9 mers
No corresponding coefficient table could be found

HLA B 5201 - 10 mers
No corresponding coefficient table could be found

HLA B 5 801 - 8 mers
No corresponding coefficient table could be found

HLA B 5 801 - 9 mers
No corresponding coefficient table could be found

HLA B 5 801 - 10 mers
No corresponding coefficient table could be found

HLA Cw 0301 - 8 mers
No corresponding coefficient table could be found

HLA Cw 0301 - 9 mers
No corresponding coefficient table could be found

HLA Cw 0301 - 10 mers
No corresponding coefficient table could be found

HLA Cw 0401 - 8 mers
No corresponding coefficient table could be found

HLA Cw 0401 - 9 mers
No corresponding coefficient table could be found

HLA Cw 0401 - 10 mers
No corresponding coefficient table could be found

HLA Cw 0602 - 8 mers
No corresponding coefficient table could be found

HLA Cw 0602 - 9 mers
No corresponding coefficient table could be found

HLA Cw 0602 - 10 mers
No corresponding coefficient table could be found

HLA Cw 0702 - 8 mers
No corresponding coefficient table could be found

HLA Cw 0702 - 9 mers
No corresponding coefficient table could be found

HLA Cw 0702 - 10 mers
No corresponding coefficient table could be found

H2 Db - 8 mers
No corresponding coefficient table could be found

H2 Db - 9 mers				
Max. score that could've been reached using this molecule type				35
**Rank**	**Start position**	**Sequence**	**% of max. score**	**Score**
1	1	VEPHTGDYL	51.42 %	18
2	48	SGVDPAQTV	48.57 %	17
3	50	VDPAQTVVL	42.85 %	15
4	52	PAQTVVLEL	42.85 %	15

H2 Db - 10 mers	
Max. score that could've been reached using this molecule type	35

Rank	Start position	Sequence	% of max. score	Score
1	1	VEPHTGDYLA	51.42 %	18
2	48	SGVDPAQTVV	48.57 %	17
3	50	VDPAQTVVLE	42.85 %	15
4	52	PAQTVVLELD	42.85 %	15

H2 Db revised - 8 mers
No corresponding coefficient table could be found

H2 Db revised - 9 mers
No corresponding coefficient table could be found

H2 Db revised - 10 mers
No corresponding coefficient table could be found

H2 Dd - 8 mers
No corresponding coefficient table could be found

H2 Dd - 9 mers
No corresponding coefficient table could be found

H2 Dd - 10 mers
No corresponding coefficient table could be found

H2 Kb - 8 mers				
Max. score that could've been reached using this molecule type				31
Rank	Start position	Sequence	% of max. score	Score
1	74	HRDWFEDL	67.74 %	21
2	33	TLGDYGDI	54.83 %	17
3	35	GDYGDISL	54.83 %	17
4	19	KTASFTTQ	38.70 %	12

H2 Kb - 9 mers
No corresponding coefficient table could be found

H2 Kb - 10 mers
No corresponding coefficient table could be found

H2 Kd - 8 mers
No corresponding coefficient table could be found

H2 Kd - 9 mers				
Max. score that could've been reached using this molecule type				38
Rank	Start position	Sequence	% of max. score	Score
1	24	TTQSEKTIL	57.89 %	22
2	52	PAQTVVLEL	47.36 %	18
3	7	DYLAANESH	44.73 %	17
4	48	SGVDPAQTV	44.73 %	17

H2 Kd - 10 mers				
Max. score that could've been reached using this molecule type				38
Rank	Start position	Sequence	% of max. score	Score
1	24	TTQSEKTILT	57.89 %	22
2	52	PAQTVVLELD	47.36 %	18
3	7	DYLAANESHS	44.73 %	17
4	48	SGVDPAQTVV	44.73 %	17

H2 KD - 8 mers
No corresponding coefficient table could be found

H2 KD - 9 mers
No corresponding coefficient table could be found

H2 KD - 10 mers

No corresponding coefficient table could be found

H2 Kk - 8 mers				
Max. score that could've been reached using this molecule type				30
**Rank**	**Start position**	**Sequence**	**% of max. score**	**Score**
1	50	VDPAQTVV	56.66 %	17
2	24	TTQSEKTI	46.66 %	14
3	27	SEKTILTL	46.66 %	14
4	33	TLGDYGDI	43.33 %	13

H2 Kk - 9 mers				
Max. score that could've been reached using this molecule type				30
**Rank**	**Start position**	**Sequence**	**% of max. score**	**Score**
1	100	VEFGEPHAV	70 %	21
2	38	GDISLTCRV	53.33 %	16
3	23	FTTQSEKTI	46.66 %	14
4	32	LTLGDYGDI	46.66 %	14

H2 Kk - 10 mers				
Max. score that could've been reached using this molecule type				30
**Rank**	**Start position**	**Sequence**	**% of max. score**	**Score**
1	100	VEFGEPHAVK	70 %	21
2	38	GDISLTCRVT	53.33 %	16
3	23	FTTQSEKTIL	46.66 %	14
4	32	LTLGDYGDIS	46.66 %	14

H2 Ld - 8 mers
No corresponding coefficient table could be found

H2 Ld - 9 mers				
Max. score that could've been reached using this molecule type				31
**Rank**	**Start position**	**Sequence**	**% of max. score**	**Score**
1	15	HSNRKTASF	61.29 %	19
2	26	QSEKTILTL	61.29 %	19
3	104	EPHAVKMDI	58.06 %	18
4	50	VDPAQTVVL	45.16 %	14

H2 Ld - 10 mers				
Max. score that could've been reached using this molecule type				31
**Rank**	**Start position**	**Sequence**	**% of max. score**	**Score**
1	15	HSNRKTASFT	61.29 %	19
2	26	QSEKTILTLG	61.29 %	19
3	104	EPHAVKMDIF	58.06 %	18
4	50	VDPAQTVVLE	45.16 %	14

Cattle A20 - 8 mers
No corresponding coefficient table could be found

Cattle A20 - 9 mers
No corresponding coefficient table could be found

Cattle A20 - 10 mers
No corresponding coefficient table could be found

The protein sequence was :
KVEPHTGDYLAANESHSNRKTASFTTQSEKTILTLGDYGDISLTCRVTSGVDPAQTVVLELDKTAEHLPKAWQVHRDWFEDLSLPWRHEGAQEWNHADRLVEFGEPHAVKMD

Query results

Parameters	
Length of peptide sequence	
Calculated subsequence scores	
Max. number of results	4
Min. score	1
Matrices to get values	SYFPEITHI

Go to
HLA A1 - 8 mers, HLA A1 - 9 mers, HLA A1 - 10 mers,
HLA A3 - 8 mers, HLA A3 - 9 mers, HLA A3 - 10 mers,
HLA A24 - 8 mers, HLA A24 - 9 mers, HLA A24 - 10 mers,
HLA A26 - 8 mers, HLA A26 - 9 mers, HLA A26 - 10 mers,
HLA A68.1 - 8 mers, HLA A68.1 - 9 mers, HLA A68.1 - 10 mers,
HLA A 0201 - 8 mers, HLA A 0201 - 9 mers, HLA A 0201 - 10 mers,
HLA A 02 05 - 8 mers, HLA A 02 05 - 9 mers, HLA A 02 05 - 10 mers,
HLA A 1101 - 8 mers, HLA A 1101 - 9 mers, HLA A 1101 - 10 mers,
HLA A 3101 - 8 mers, HLA A 3101 - 9 mers, HLA A 3101 - 10 mers,
HLA A 3302 - 8 mers, HLA A 3302 - 9 mers, HLA A 3302 - 10 mers,
HLA B14 - 8 mers, HLA B14 - 9 mers, HLA B14 - 10 mers,
HLA B40 - 8 mers, HLA B40 - 9 mers, HLA B40 - 10 mers,
HLA B60 - 8 mers, HLA B60 - 9 mers, HLA B60 - 10 mers,
HLA B61 - 8 mers, HLA B61 - 9 mers, HLA B61 - 10 mers,
HLA B62 - 8 mers, HLA B62 - 9 mers, HLA B62 - 10 mers,
HLA B7 - 8 mers, HLA B7 - 9 mers, HLA B7 - 10 mers,
HLA B8 - 8 mers, HLA B8 - 9 mers, HLA B8 - 10 mers,
HLA B 0702 - 8 mers, HLA B 0702 - 9 mers, HLA B 0702 - 10 mers,
HLA B 1510 - 8 mers, HLA B 1510 - 9 mers, HLA B 1510 - 10 mers,
HLA B 2702 - 8 mers, HLA B 2702 - 9 mers, HLA B 2702 - 10 mers,
HLA B 2705 - 8 mers, HLA B 2705 - 9 mers, HLA B 2705 - 10 mers,
HLA B 3501 - 8 mers, HLA B 3501 - 9 mers, HLA B 3501 - 10 mers,
HLA B 3701 - 8 mers, HLA B 3701 - 9 mers, HLA B 3701 - 10 mers,
HLA B 3801 - 8 mers, HLA B 3801 - 9 mers, HLA B 3801 - 10 mers,
HLA B 3 901 - 8 mers, HLA B 3 901 - 9 mers, HLA B 3 901 - 10 mers,
HLA B 4403 - 8 mers, HLA B 4403 - 9 mers, HLA B 4403 - 10 mers,
HLA B 5101 - 8 mers, HLA B 5101 - 9 mers, HLA B 5101 - 10 mers,
HLA B 5102 - 8 mers, HLA B 5102 - 9 mers, HLA B 5102 - 10 mers,
HLA B 5103 - 8 mers, HLA B 5103 - 9 mers, HLA B 5103 - 10 mers,
HLA B 5201 - 8 mers, HLA B 5201 - 9 mers, HLA B 5201 - 10 mers,
HLA B 5 801 - 8 mers, HLA B 5 801 - 9 mers, HLA B 5 801 - 10 mers,
HLA Cw 0301 - 8 mers, HLA Cw 0301 - 9 mers, HLA Cw 0301 - 10 mers,
HLA Cw 0401 - 8 mers, HLA Cw 0401 - 9 mers, HLA Cw 0401 - 10 mers,
HLA Cw 0602 - 8 mers, HLA Cw 0602 - 9 mers, HLA Cw 0602 - 10 mers,
HLA Cw 0702 - 8 mers, HLA Cw 0702 - 9 mers, HLA Cw 0702 - 10 mers,
H2 Db - 8 mers, H2 Db - 9 mers, H2 Db - 10 mers,
H2 Db revised - 8 mers, H2 Db revised - 9 mers, H2 Db revised - 10 mers,
H2 Dd - 8 mers, H2 Dd - 9 mers, H2 Dd - 10 mers,
H2 Kb - 8 mers, H2 Kb - 9 mers, H2 Kb - 10 mers,
H2 Kd - 8 mers, H2 Kd - 9 mers, H2 Kd - 10 mers,
H2 KD - 8 mers, H2 KD - 9 mers, H2 KD - 10 mers,
H2 Kk - 8 mers, H2 Kk - 9 mers, H2 Kk - 10 mers,
H2 Ld - 8 mers, H2 Ld - 9 mers, H2 Ld - 10 mers,
Cattle A20 - 8 mers, Cattle A20 - 9 mers, Cattle A20 - 10 mers,

HLA A1 - 8 mers
No corresponding coefficient table could be found

HLA A1 - 9 mers				
Max. score that could've been reached using this molecule type				50
**Rank**	**Start position**	**Sequence**	**% of max. score**	**Score**
1	112	VYDVNKITY	60 %	30
2	103	ETKKKATGY	42 %	21
3	105	KKKATGYVY	34 %	17
4	5	VLELGGCVT	32 %	16

HLA A1 - 10 mers				
Max. score that could've been reached using this molecule type				50
**Rank**	**Start position**	**Sequence**	**% of max. score**	**Score**
1	112	VYDVNKITYV	60 %	30
2	103	ETKKKATGYV	42 %	21
3	105	KKKATGYVYD	34 %	17
4	5	VLELGGCVTL	32 %	16

HLA A3 - 8 mers
No corresponding coefficient table could be found

HLA A3 - 9 mers				
Max. score that could've been reached using this molecule type				43
**Rank**	**Start position**	**Sequence**	**% of max. score**	**Score**
1	11	CVTLTAEGK	53.48 %	23
2	45	KLANSKVAA	46.51 %	20
3	5	VLELGGCVT	44.18 %	19
4	28	DIHQENPAK	44.18 %	19

HLA A3 - 10 mers				
Max. score that could've been reached using this molecule type				43
**Rank**	**Start position**	**Sequence**	**% of max. score**	**Score**
1	11	CVTLTAEGKP	53.48 %	23
2	45	KLANSKVAAR	46.51 %	20
3	5	VLELGGCVTL	44.18 %	19
4	28	DIHQENPAKT	44.18 %	19

HLA A24 - 8 mers
No corresponding coefficient table could be found

HLA A24 - 9 mers
No corresponding coefficient table could be found

HLA A24 - 10 mers
No corresponding coefficient table could be found

HLA A26 - 8 mers
No corresponding coefficient table could be found

HLA A26 - 9 mers
No corresponding coefficient table could be found

HLA A26 - 10 mers
No corresponding coefficient table could be found

HLA A68.1 - 8 mers
No corresponding coefficient table could be found

HLA A68.1 - 9 mers
No corresponding coefficient table could be found

HLA A68.1 - 10 mers
No corresponding coefficient table could be found

HLA A 0201 - 8 mers
No corresponding coefficient table could be found

HLA A 0201 - 9 mers				
Max. score that could've been reached using this molecule type				36
**Rank**	**Start position**	**Sequence**	**% of max. score**	**Score**
1	14	LTAEGKPSV	69.44 %	25
2	87	GLFGKGSIV	69.44 %	25
3	6	LELGGCVTL	61.11 %	22
4	107	KATGYVYDV	61.11 %	22

HLA A 0201 - 10 mers				
Max. score that could've been reached using this molecule type				36
**Rank**	**Start position**	**Sequence**	**% of max. score**	**Score**
1	14	LTAEGKPSVD	69.44 %	25
2	87	GLFGKGSIVA	69.44 %	25
3	6	LELGGCVTLT	61.11 %	22
4	107	KATGYVYDVN	61.11 %	22

HLA A 02 05 - 8 mers
No corresponding coefficient table could be found

HLA A 02 05 - 9 mers
No corresponding coefficient table could be found

HLA A 02 05 - 10 mers
No corresponding coefficient table could be found

HLA A 1101 - 8 mers
No corresponding coefficient table could be found

HLA A 1101 - 9 mers
No corresponding coefficient table could be found

HLA A 1101 - 10 mers
No corresponding coefficient table could be found

HLA A 3101 - 8 mers
No corresponding coefficient table could be found

HLA A 3101 - 9 mers
No corresponding coefficient table could be found

HLA A 3101 - 10 mers
No corresponding coefficient table could be found

HLA A 3302 - 8 mers
No corresponding coefficient table could be found

HLA A 3302 - 9 mers
No corresponding coefficient table could be found

HLA A 3302 - 10 mers
No corresponding coefficient table could be found

HLA B14 - 8 mers
No corresponding coefficient table could be found

HLA B14 - 9 mers
No corresponding coefficient table could be found

HLA B14 - 10 mers
No corresponding coefficient table could be found

HLA B40 - 8 mers
No corresponding coefficient table could be found

HLA B40 - 9 mers
No corresponding coefficient table could be found

HLA B40 - 10 mers
No corresponding coefficient table could be found

HLA B60 - 8 mers
No corresponding coefficient table could be found

HLA B60 - 9 mers
No corresponding coefficient table could be found

HLA B60 - 10 mers
No corresponding coefficient table could be found

HLA B61 - 8 mers
No corresponding coefficient table could be found

HLA B61 - 9 mers
No corresponding coefficient table could be found

HLA B61 - 10 mers
No corresponding coefficient table could be found

HLA B62 - 8 mers
No corresponding coefficient table could be found

HLA B62 - 9 mers
No corresponding coefficient table could be found

HLA B62 - 10 mers
No corresponding coefficient table could be found

HLA B7 - 8 mers
No corresponding coefficient table could be found

HLA B7 - 9 mers
No corresponding coefficient table could be found

HLA B7 - 10 mers
No corresponding coefficient table could be found

HLA B8 - 8 mers				
Max. score that could've been reached using this molecule type				40
**Rank**	**Start position**	**Sequence**	**% of max. score**	**Score**
1	87	GLFGKGSI	57.5 %	23
2	34	PAKTREYC	55 %	22
3	103	ETKKKATG	55 %	22
4	7	ELGGCVTL	45 %	18

HLA B8 - 9 mers				
Max. score that could've been reached using this molecule type				43
**Rank**	**Start position**	**Sequence**	**% of max. score**	**Score**
1	34	PAKTREYCL	74.41 %	32
2	103	ETKKKATGY	51.16 %	22
3	43	HAKLANSKV	39.53 %	17
4	87	GLFGKGSIV	39.53 %	17

HLA B8 - 10 mers				
Max. score that could've been reached using this molecule type				43
**Rank**	**Start position**	**Sequence**	**% of max. score**	**Score**
1	34	PAKTREYCLH	74.41 %	32
2	103	ETKKKATGYV	51.16 %	22
3	43	HAKLANSKVA	39.53 %	17
4	87	GLFGKGSIVA	39.53 %	17

HLA B 0702 - 8 mers
No corresponding coefficient table could be found

HLA B 0702 - 9 mers				
Max. score that could've been reached using this molecule type				35
**Rank**	**Start position**	**Sequence**	**% of max. score**	**Score**
1	55	CPAMGPATL	62.85 %	22
2	63	LPEEHQAST	48.57 %	17
3	16	AEGKPSVDV	42.85 %	15
4	6	LELGGCVTL	40 %	14

HLA B 0702 - 10 mers	
Max. score that could've been reached using this molecule type	35

Rank	Start position	Sequence	% of max. score	Score
1	55	CPAMGPATLP	62.85 %	22
2	63	LPEEHQASTV	48.57 %	17
3	16	AEGKPSVDVW	42.85 %	15
4	6	LELGGCVTLT	40 %	14

HLA B 1510 - 8 mers
No corresponding coefficient table could be found

HLA B 1510 - 9 mers				
Max. score that could've been reached using this molecule type				31
Rank	Start position	Sequence	% of max. score	Score
1	6	LELGGCVTL	48.38 %	15
2	29	IHQENPAKT	48.38 %	15
3	55	CPAMGPATL	45.16 %	14
4	0	TRVSLVLEL	41.93 %	13

HLA B 1510 - 10 mers				
Max. score that could've been reached using this molecule type				31
Rank	Start position	Sequence	% of max. score	Score
1	6	LELGGCVTLT	48.38 %	15
2	29	IHQENPAKTR	48.38 %	15
3	55	CPAMGPATLP	45.16 %	14
4	0	TRVSLVLELG	41.93 %	13

HLA B 2702 - 8 mers
No corresponding coefficient table could be found

HLA B 2702 - 9 mers
No corresponding coefficient table could be found

HLA B 2702 - 10 mers
No corresponding coefficient table could be found

HLA B 2705 - 8 mers
No corresponding coefficient table could be found

HLA B 2705 - 9 mers				
Max. score that could've been reached using this molecule type				37
Rank	Start position	Sequence	% of max. score	Score
1	0	TRVSLVLEL	67.56 %	25
2	37	TREYCLHAK	56.75 %	21
3	38	REYCLHAKL	54.05 %	20
4	6	LELGGCVTL	48.64 %	18

HLA B 2705 - 10 mers				
Max. score that could've been reached using this molecule type				37
Rank	Start position	Sequence	% of max. score	Score
1	0	TRVSLVLELG	67.56 %	25
2	37	TREYCLHAKL	56.75 %	21
3	38	REYCLHAKLA	54.05 %	20
4	6	LELGGCVTLT	48.64 %	18

HLA B 3501 - 8 mers
No corresponding coefficient table could be found

HLA B 3501 - 9 mers
No corresponding coefficient table could be found

HLA B 3501 - 10 mers
No corresponding coefficient table could be found

HLA B 3701 - 8 mers
No corresponding coefficient table could be found

HLA B 3701 - 9 mers
No corresponding coefficient table could be found

HLA B 3701 - 10 mers
No corresponding coefficient table could be found

HLA B 3801 - 8 mers
No corresponding coefficient table could be found

HLA B 3801 - 9 mers
No corresponding coefficient table could be found

HLA B 3801 - 10 mers
No corresponding coefficient table could be found

HLA B 3 901 - 8 mers
No corresponding coefficient table could be found

HLA B 3 901 - 9 mers
No corresponding coefficient table could be found

HLA B 3 901 - 10 mers
No corresponding coefficient table could be found

HLA B 4403 - 8 mers
No corresponding coefficient table could be found

HLA B 4403 - 9 mers
No corresponding coefficient table could be found

HLA B 4403 - 10 mers
No corresponding coefficient table could be found

HLA B 5101 - 8 mers
No corresponding coefficient table could be found

HLA B 5101 - 9 mers
No corresponding coefficient table could be found

HLA B 5101 - 10 mers
No corresponding coefficient table could be found

HLA B 5102 - 8 mers
No corresponding coefficient table could be found

HLA B 5102 - 9 mers
No corresponding coefficient table could be found

HLA B 5102 - 10 mers
No corresponding coefficient table could be found

HLA B 5103 - 8 mers
No corresponding coefficient table could be found

HLA B 5103 - 9 mers
No corresponding coefficient table could be found

HLA B 5103 - 10 mers
No corresponding coefficient table could be found

HLA B 5201 - 8 mers
No corresponding coefficient table could be found

HLA B 5201 - 9 mers
No corresponding coefficient table could be found

HLA B 5201 - 10 mers
No corresponding coefficient table could be found

HLA B 5 801 - 8 mers
No corresponding coefficient table could be found

HLA B 5 801 - 9 mers
No corresponding coefficient table could be found

HLA B 5 801 - 10 mers
No corresponding coefficient table could be found

HLA Cw 0301 - 8 mers
No corresponding coefficient table could be found

HLA Cw 0301 - 9 mers
No corresponding coefficient table could be found

HLA Cw 0301 - 10 mers
No corresponding coefficient table could be found

HLA Cw 0401 - 8 mers
No corresponding coefficient table could be found

HLA Cw 0401 - 9 mers
No corresponding coefficient table could be found

HLA Cw 0401 - 10 mers
No corresponding coefficient table could be found

HLA Cw 0602 - 8 mers
No corresponding coefficient table could be found

HLA Cw 0602 - 9 mers
No corresponding coefficient table could be found

HLA Cw 0602 - 10 mers
No corresponding coefficient table could be found

HLA Cw 0702 - 8 mers
No corresponding coefficient table could be found

HLA Cw 0702 - 9 mers
No corresponding coefficient table could be found

HLA Cw 0702 - 10 mers
No corresponding coefficient table could be found

H2 Db - 8 mers
No corresponding coefficient table could be found

H2 Db - 9 mers				
Max. score that could've been reached using this molecule type				35
**Rank**	**Start position**	**Sequence**	**% of max. score**	**Score**
1	80	RGWGNHCGL	60 %	21
2	21	SVDVWLDDI	48.57 %	17
3	0	TRVSLVLEL	45.71 %	16
4	6	LELGGCVTL	42.85 %	15

H2 Db - 10 mers	
Max. score that could've been reached using this molecule type	35

Rank	Start position	Sequence	% of max. score	Score
1	80	RGWGNHCGLF	60 %	21
2	21	SVDVWLDDIH	48.57 %	17
3	0	TRVSLVLELG	45.71 %	16
4	6	LELGGCVTLT	42.85 %	15

**H2 Db revised - 8 mers**
No corresponding coefficient table could be found

**H2 Db revised - 9 mers**
No corresponding coefficient table could be found

**H2 Db revised - 10 mers**
No corresponding coefficient table could be found

**H2 Dd - 8 mers**
No corresponding coefficient table could be found

**H2 Dd - 9 mers**
No corresponding coefficient table could be found

**H2 Dd - 10 mers**
No corresponding coefficient table could be found

**H2 Kb - 8 mers**				
Max. score that could've been reached using this molecule type				31
**Rank**	**Start position**	**Sequence**	**% of max. score**	**Score**
1	109	TGYVYDVN	54.83 %	17
2	111	YVYDVNKI	45.16 %	14
3	96	ACAKFSCE	41.93 %	13
4	35	AKTREYCL	38.70 %	12

**H2 Kb - 9 mers**
No corresponding coefficient table could be found

**H2 Kb - 10 mers**
No corresponding coefficient table could be found

**H2 Kd - 8 mers**
No corresponding coefficient table could be found

**H2 Kd - 9 mers**				
Max. score that could've been reached using this molecule type				38
**Rank**	**Start position**	**Sequence**	**% of max. score**	**Score**
1	110	GYVYDVNKI	60.52 %	23
2	39	EYCLHAKLA	52.63 %	20
3	0	TRVSLVLEL	50 %	19
4	86	CGLFGKGSI	42.10 %	16

**H2 Kd - 10 mers**				
Max. score that could've been reached using this molecule type				38
**Rank**	**Start position**	**Sequence**	**% of max. score**	**Score**
1	110	GYVYDVNKIT	60.52 %	23
2	39	EYCLHAKLAN	52.63 %	20
3	0	TRVSLVLELG	50 %	19
4	86	CGLFGKGSIV	42.10 %	16

**H2 KD - 8 mers**
No corresponding coefficient table could be found

**H2 KD - 9 mers**
No corresponding coefficient table could be found

**H2 KD - 10 mers**

No corresponding coefficient table could be found

H2 Kk - 8 mers				
Max. score that could've been reached using this molecule type				30
**Rank**	**Start position**	**Sequence**	**% of max. score**	**Score**
1	65	EEHQASTV	70 %	21
2	22	VDVWLDDI	63.33 %	19
3	31	QENPAKTR	53.33 %	16
4	6	LELGGCVT	50 %	15

H2 Kk - 9 mers				
Max. score that could've been reached using this molecule type				30
**Rank**	**Start position**	**Sequence**	**% of max. score**	**Score**
1	64	PEEHQASTV	66.66 %	20
2	16	AEGKPSVDV	63.33 %	19
3	113	YDVNKITYV	53.33 %	16
4	110	GYVYDVNKI	50 %	15

H2 Kk - 10 mers				
Max. score that could've been reached using this molecule type				30
**Rank**	**Start position**	**Sequence**	**% of max. score**	**Score**
1	64	PEEHQASTVC	66.66 %	20
2	16	AEGKPSVDVW	63.33 %	19
3	110	GYVYDVNKIT	50 %	15
4	6	LELGGCVTLT	46.66 %	14

H2 Ld - 8 mers
No corresponding coefficient table could be found

H2 Ld - 9 mers				
Max. score that could've been reached using this molecule type				31
**Rank**	**Start position**	**Sequence**	**% of max. score**	**Score**
1	55	CPAMGPATL	74.19 %	23
2	92	GSIVACAKF	61.29 %	19
3	6	LELGGCVTL	45.16 %	14
4	33	NPAKTREYC	45.16 %	14

H2 Ld - 10 mers				
Max. score that could've been reached using this molecule type				31
**Rank**	**Start position**	**Sequence**	**% of max. score**	**Score**
1	55	CPAMGPATLP	74.19 %	23
2	92	GSIVACAKFS	61.29 %	19
3	6	LELGGCVTLT	45.16 %	14
4	33	NPAKTREYCL	45.16 %	14

Cattle A20 - 8 mers
No corresponding coefficient table could be found

Cattle A20 - 9 mers
No corresponding coefficient table could be found

Cattle A20 - 10 mers
No corresponding coefficient table could be found

The protein sequence was :

TRVSLVLELGGCVTLTAEGKPSVDVWLDDIHQENPAKTREYCLHAKLANSKVAARCPAMGPATLPEEHQASTVCRRDQSDRGWGNHCGLFGKGSIVACAKFSCETKKKATGY

Supplementary Table 5:
List of epitopes predicted by IEDB-combined predictor.

Allele	PepLength	Sequence	Proteasome Score	TAP Score	MHC Score	Processing Score	Total Score	MHC IC50[nM]
HLA-B*35:01	10	YVYDVNKITY	1.48	1.39	-1.5	2.87	1.37	31.55
HLA-A*30:02	9	KTILTLGDY	1.24	1.26	-1.23	2.51	1.27	17.03
HLA-B*40:01	9	REYCLHAKL	1.54	0.55	-0.93	2.09	1.16	8.43
HLA-B*40:01	9	LELGGCVTL	1.76	0.39	-1.04	2.15	1.11	10.93
HLA-A*30:02	9	KVEPHTGDY	0.96	1.26	-1.37	2.22	0.86	23.3
HLA-B*35:01	9	LPPGDNIIY	1.45	1.11	-1.77	2.57	0.8	59.09
HLA-A*26:01	9	ETKKKATGY	1.2	1.17	-1.59	2.37	0.78	38.75
HLA-B*58:01	10	KTAEHLPKAW	1.5	0.37	-1.12	1.87	0.75	13.13
HLA-B*15:01	10	YVYDVNKITY	1.48	1.39	-2.18	2.87	0.69	151.47
HLA-A*23:01	10	IYVGELSHQW	1.31	0.55	-1.18	1.86	0.68	15.12
HLA-B*58:01	10	KAWQVHRDWF	1.37	1.18	-1.9	2.55	0.65	79.14
HLA-A*02:03	9	KMKGMTYTV	1.22	0.25	-0.98	1.47	0.49	9.6
HLA-A*24:02	10	IYVGELSHQW	1.31	0.55	-1.4	1.86	0.45	25.41
HLA-A*30:01	9	KTREYCLHA	1.21	-0.11	-0.7	1.11	0.41	5.01
HLA-A*11:01	9	STIGRVLEK	0.92	0.23	-0.74	1.15	0.41	5.47
HLA-A*31:01	10	KLANSKVAAR	0.93	0.77	-1.33	1.71	0.38	21.37
HLA-B*58:01	9	KAWQVHRDW	1.08	0.46	-1.17	1.55	0.37	14.95
HLA-A*68:01	10	LAANESHSNR	1	0.65	-1.33	1.66	0.33	21.14
HLA-B*07:02	9	CPAMGPATL	1.42	0.33	-1.43	1.75	0.32	26.82
HLA-B*57:01	10	KAWQVHRDWF	1.37	1.18	-2.26	2.55	0.28	184.04
HLA-A*11:01	9	ASFTTQSEK	0.97	0.34	-1.04	1.31	0.27	10.93

**HLA-A*02:03**	**9**	**ILLKSLAGV**	**0.81**	**0.15**	**-0.72**	**0.96**	**0.24**	**5.29**
**HLA-A*26:01**	**9**	**DTVVMEVTY**	**1.52**	**1.15**	**-2.49**	**2.67**	**0.18**	**306.02**
**HLA-B*57:01**	**9**	**KAWQVHRDW**	**1.08**	**0.46**	**-1.36**	**1.55**	**0.18**	**23.05**
**HLA-A*02:01**	**9**	**ILLKSLAGV**	**0.81**	**0.15**	**-0.79**	**0.96**	**0.17**	**6.16**
**HLA-B*57:01**	**10**	**KTAEHLPKAW**	**1.5**	**0.37**	**-1.69**	**1.87**	**0.17**	**49.16**
**HLA-A*02:03**	**10**	**SLTCRVTSGV**	**0.82**	**0.16**	**-0.82**	**0.97**	**0.16**	**6.57**
**HLA-A*02:03**	**10**	**SLAGVPVANI**	**1.03**	**0.31**	**-1.21**	**1.35**	**0.14**	**16.31**
**HLA-B*58:01**	**9**	**YVGELSHQW**	**1.31**	**0.37**	**-1.56**	**1.68**	**0.11**	**36.71**
**HLA-A*26:01**	**9**	**YTVCEGSKF**	**1.26**	**1.12**	**-2.28**	**2.38**	**0.1**	**190.11**
**HLA-A*68:01**	**10**	**MTYTVCEGSK**	**0.76**	**0.28**	**-0.95**	**1.05**	**0.09**	**8.99**
**HLA-B*40:01**	**9**	**QEWNHADRL**	**1.39**	**0.5**	**-1.8**	**1.9**	**0.09**	**63.74**
**HLA-B*35:01**	**9**	**DTVVMEVTY**	**1.52**	**1.15**	**-2.62**	**2.67**	**0.05**	**414.31**
**HLA-A*11:01**	**10**	**YVYDVNKITY**	**1.48**	**1.39**	**-2.83**	**2.87**	**0.04**	**674.19**

Supplementary Table 6:
List of epitopes predicted by IEDB-MHC-II tool.

Allele	Peptide	Percentile rank
HLA-DRB3*01:01	KPSVDVWLDDIHQEN	0.1
HLA-DRB3*01:01	PSVDVWLDDIHQENP	0.11
HLA-DRB3*01:01	SVDVWLDDIHQENPA	0.11
HLA-DRB3*01:01	VDVWLDDIHQENPAK	0.12
HLA-DRB3*01:01	DVWLDDIHQENPAKT	0.26
HLA-DRB3*01:01	KKKATGYVYDVNKIT	0.38
HLA-DRB3*01:01	KKATGYVYDVNKITY	0.38
HLA-DRB3*01:01	KATGYVYDVNKITYV	0.39
HLA-DQA1*01:01/DQB1*05:01	KAWQVHRDWFEDLSL	0.02
HLA-DQA1*01:01/DQB1*05:01	AWQVHRDWFEDLSLP	0.02
HLA-DQA1*01:01/DQB1*05:01	WQVHRDWFEDLSLPW	0.02
HLA-DQA1*01:01/DQB1*05:01	QVHRDWFEDLSLPWR	0.04
HLA-DQA1*01:01/DQB1*05:01	VHRDWFEDLSLPWRH	0.05
HLA-DQA1*01:01/DQB1*05:01	PKAWQVHRDWFEDLS	0.23
HLA-DQA1*01:01/DQB1*05:01	HRDWFEDLSLPWRHE	0.33
HLA-DRB1*12:01	TGILLKSLAGVPVAN	0.42
HLA-DRB1*12:01	QTGILLKSLAGVPVA	0.43
HLA-DRB1*12:01	DQTGILLKSLAGVPV	0.44
HLA-DRB1*08:02	LLKSLAGVPVANIEG	0.45
HLA-DRB1*08:02	LKSLAGVPVANIEGS	0.45

Supplementary Table 7:
List of epitopes predicted by MHC2Pred tool.

MHC2Pred
SVM based method for prediction of promiscuous MHC class II binders

Home
Help
Information
Team
Contact

Antigen Name	Untitled
Scanned on	Mon Apr 29 02:52:51 2019
Length of input sequence	122 amino acids
Number of nonamers from input sequence	114
Threshold setting	1.5
Number of alleles in query	42

ALLELE: HLA-DR1

Threshold 1.5 as cutoff score

Prediction method	Rank	Sequence	Residue No.	Peptide Score
SVM	1	LANSKVAAR	47	0.728
SVM	2	VAARCPAMG	52	0.685
SVM	3	KATGYVYDV	108	0.394
SVM	4	VCRRDQSDR	73	0.292

ALLELE: HLA-DR4

Threshold 1.5 as cutoff score

Prediction method	Rank	Sequence	Residue No.	Peptide Score
SVM	1	LHAKLANSK	43	1.214
SVM	2	LTAEGKPSV	15	0.669
SVM	3	NPAKTREYC	34	0.656
SVM	4	STVCRRDQS	71	0.598

ALLELE: HLA-DR9

Threshold 1.5 as cutoff score

Prediction method	Rank	Sequence	Residue No.	Peptide Score
SVM	1	GWGNHCGLF	82	1.366
SVM	2	CGLFGKGSI	87	1.325
SVM	3	DQSDRGWGN	77	1.239
SVM	4	QSDRGWGNH	78	1.198

ALLELE: HLA-DR11

Threshold 1.5 as cutoff score

Prediction method	Rank	Sequence	Residue No.	Peptide Score
SVM	1	GSIVACAKF	93	0.183
SVM	2	ANSKVAARC	48	0.136
SVM	3	KGSIVACAK	92	0.122
SVM	4	GKPSVDVWL	19	0.105

ALLELE: HLA-DR2

Threshold 1.5 as cutoff score

Prediction method	Rank	Sequence	Residue No.	Peptide Score
SVM	1	KGSIVACAK	92	0.970
SVM	2	YVYDVNKIT	112	0.876

**SVM**	3	LHAKLANSK	43	0.609
**SVM**	4	ANSKVAARC	48	0.589

**ALLELE: HLA-DR3**				
Threshold 1.5 as cutoff score				
Prediction method	Rank	Sequence	Residue No.	Peptide Score
**SVM**	1	QASTVCRRD	69	1.121
**SVM**	2	PAKTREYCL	35	1.111
**SVM**	3	PATLPEEHQ	61	0.993
**SVM**	4	KKATGYVYD	107	0.940

**ALLELE: HLA-DR5**				
Threshold 1.5 as cutoff score				
Prediction method	Rank	Sequence	Residue No.	Peptide Score
**SVM**	1	GYVYDVNKI	111	0.114
**SVM**	2	IVACAKFSC	95	-0.015
**SVM**	3	GSIVACAKF	93	-0.051
**SVM**	4	LTAEGKPSV	15	-0.084

**ALLELE: HLA-DR7**				
Threshold 1.5 as cutoff score				
Prediction method	Rank	Sequence	Residue No.	Peptide Score
**SVM**	1	AKLANSKVA	45	1.424
**SVM**	2	KKKATGYVY	106	0.470
**SVM**	3	SIVACAKFS	94	0.368
**SVM**	4	SKVAARCPA	50	0.322

**ALLELE: HLA-DR8**				
Threshold 1.5 as cutoff score				
Prediction method	Rank	Sequence	Residue No.	Peptide Score
**SVM**	1	ASTVCRRDQ	70	0.652
**SVM**	2	HQASTVCRR	68	0.543
**SVM**	3	QASTVCRRD	69	0.478
**SVM**	4	DQSDRGWGN	77	0.437

**ALLELE: HLA-DR13**				
Threshold 1.5 as cutoff score				
Prediction method	Rank	Sequence	Residue No.	Peptide Score
**SVM**	1	VAARCPAMG	52	1.299
**SVM**	2	KVAARCPAM	51	1.250
**SVM**	3	KLANSKVAA	46	1.014
**SVM**	4	VSLVLELGG	3	0.876

**ALLELE: HLA-DR15**				
Threshold 1.5 as cutoff score				
Prediction method	Rank	Sequence	Residue No.	Peptide Score
**SVM**	1	SIVACAKFS	94	1.654
**SVM**	2	SKVAARCPA	50	0.924
**SVM**	3	ENPAKTREY	33	0.734
**SVM**	4	GLFGKGSIV	88	0.642

ALLELE: HLA-DR51				
Threshold 1.5 as cutoff score				
Prediction method	Rank	Sequence	Residue No.	Peptide Score
**SVM**	1	HQENPAKTR	31	1.123
**SVM**	2	AMGPATLPE	58	0.483
**SVM**	3	AARCPAMGP	53	0.446
**SVM**	4	NSKVAARCP	49	0.433

ALLELE: HLA-DR52				
Threshold 1.5 as cutoff score				
Prediction method	Rank	Sequence	Residue No.	Peptide Score
**SVM**	1	GLFGKGSIV	88	1.115
**SVM**	2	KGSIVACAK	92	0.798
**SVM**	3	FSCETKKKA	101	0.767
**SVM**	4	PAMGPATLP	57	0.690

ALLELE: HLA-DR53				
Threshold 1.5 as cutoff score				
Prediction method	Rank	Sequence	Residue No.	Peptide Score
**SVM**	1	RVSLVLELG	2	0.839
**SVM**	2	AEGKPSVDV	17	0.690
**SVM**	3	TAEGKPSVD	16	0.608
**SVM**	4	QENPAKTRE	32	0.605

ALLELE: HLA-DQ2				
Threshold 1.5 as cutoff score				
Prediction method	Rank	Sequence	Residue No.	Peptide Score
**SVM**	1	LANSKVAAR	47	0.482
**SVM**	2	IHQENPAKT	30	0.453
**SVM**	3	LVLELGGCV	5	0.396
**SVM**	4	VSLVLELGG	3	0.312

ALLELE: HLA-DQ4				
Threshold 1.5 as cutoff score				
Prediction method	Rank	Sequence	Residue No.	Peptide Score
**SVM**	1	SIVACAKFS	94	0.951
**SVM**	2	GYVYDVNKI	111	0.856
**SVM**	3	CLHAKLANS	42	0.838
**SVM**	4	VYDVNKITY	113	0.835

ALLELE: HLA-DQ6				
Threshold 1.5 as cutoff score				
Prediction method	Rank	Sequence	Residue No.	Peptide Score
**SVM**	1	REYCLHAKL	39	1.164
**SVM**	2	EGKPSVDVW	18	1.060
**SVM**	3	CGLFGKGSI	87	0.816
**SVM**	4	VDVWLDDIH	23	0.800

ALLELE: HLA-DQ7				
Threshold 1.5 as cutoff score				
Prediction method	Rank	Sequence	Residue No.	Peptide Score
SVM	1	RCPAMGPAT	55	1.846
SVM	2	YVYDVNKIT	112	1.209
SVM	3	TLTAEGKPS	14	1.160
SVM	4	KVAARCPAM	51	1.144

ALLELE: HLA-DQ8				
Threshold 1.5 as cutoff score				
Prediction method	Rank	Sequence	Residue No.	Peptide Score
SVM	1	SVDVWLDDI	22	1.700
SVM	2	KLANSKVAA	46	1.394
SVM	3	DVWLDDIHQ	24	1.362
SVM	4	SKVAARCPA	50	1.285

ALLELE: HLA-DQA1*0501				
Threshold 1.5 as cutoff score				
Prediction method	Rank	Sequence	Residue No.	Peptide Score
SVM	1	EYCLHAKLA	40	0.365
SVM	2	GPATLPEEH	60	0.318
SVM	3	SKVAARCPA	50	0.234
SVM	4	NPAKTREYC	34	0.234

ALLELE: HLA-DQB1*02				
Threshold 1.5 as cutoff score				
Prediction method	Rank	Sequence	Residue No.	Peptide Score
SVM	1	IHQENPAKT	30	0.202
SVM	2	QENPAKTRE	32	0.120
SVM	3	GGCVTLTAE	10	0.117
SVM	4	LELGGCVTL	7	0.109

ALLELE: HLA-DQB1*0201				
Threshold 1.5 as cutoff score				
Prediction method	Rank	Sequence	Residue No.	Peptide Score
SVM	1	CVTLTAEGK	12	0.365
SVM	2	EYCLHAKLA	40	0.255
SVM	3	ELGGCVTLT	8	0.191
SVM	4	ETKKKATGY	104	0.153

ALLELE: HLA-DQB1*03				
Threshold 1.5 as cutoff score				
Prediction method	Rank	Sequence	Residue No.	Peptide Score
SVM	1	PATLPEEHQ	61	1.205
SVM	2	WLDDIHQEN	26	0.917
SVM	3	HAKLANSKV	44	0.618
SVM	4	KATGYVYDV	108	0.594

ALLELE: HLA-DQB1*0301				
Threshold 1.5 as cutoff score				
Prediction method	Rank	Sequence	Residue No.	Peptide Score
**SVM**	1	PSVDVWLDD	21	0.978
**SVM**	2	GKGSIVACA	91	0.834
**SVM**	3	KVAARCPAM	51	0.730
**SVM**	4	YDVNKITYV	114	0.683

ALLELE: HLA-DQB1*0302				
Threshold 1.5 as cutoff score				
Prediction method	Rank	Sequence	Residue No.	Peptide Score
**SVM**	1	TLPEEHQAS	63	0.207
**SVM**	2	GGCVTLTAE	10	0.184
**SVM**	3	GPATLPEEH	60	0.170
**SVM**	4	DIHQENPAK	29	0.161

ALLELE: HLA-DRB1*0101				
Threshold 1.5 as cutoff score				
Prediction method	Rank	Sequence	Residue No.	Peptide Score
**SVM**	1	GLFGKGSIV	88	1.214
**SVM**	2	CLHAKLANS	42	0.856
**SVM**	3	IVACAKFSC	95	0.670
**SVM**	4	GWGNHCGLF	82	0.646

ALLELE: HLA-DRB4*0101				
Threshold 1.5 as cutoff score				
Prediction method	Rank	Sequence	Residue No.	Peptide Score
**SVM**	1	RVSLVLELG	2	0.626
**SVM**	2	MGPATLPEE	59	0.550
**SVM**	3	PATLPEEHQ	61	0.545
**SVM**	4	DIHQENPAK	29	0.507

ALLELE: HLA-DRB5*0101				
Threshold 1.5 as cutoff score				
Prediction method	Rank	Sequence	Residue No.	Peptide Score
**SVM**	1	TLTAEGKPS	14	1.014
**SVM**	2	AMGPATLPE	58	0.695
**SVM**	3	ASTVCRRDQ	70	0.643
**SVM**	4	KVAARCPAM	51	0.642

ALLELE: HLA-DRB1*0301				
Threshold 1.5 as cutoff score				
Prediction method	Rank	Sequence	Residue No.	Peptide Score
**SVM**	1	AKTREYCLH	36	0.950
**SVM**	2	RVSLVLELG	2	0.750
**SVM**	3	QENPAKTRE	32	0.567
**SVM**	4	VDVWLDDIH	23	0.464

ALLELE: HLA-DRB1*0401
Threshold 1.5 as cutoff score

Prediction method	Rank	Sequence	Residue No.	Peptide Score
**SVM**	1	PAKTREYCL	35	0.929
**SVM**	2	AARCPAMGP	53	0.662
**SVM**	3	CPAMGPATL	56	0.645
**SVM**	4	TKKKATGYV	105	0.596

**ALLELE: HLA-DRB1*0404**				
Threshold 1.5 as cutoff score				
Prediction method	Rank	Sequence	Residue No.	Peptide Score
**SVM**	1	KLANSKVAA	46	0.585
**SVM**	2	SLVLELGGC	4	0.571
**SVM**	3	LELGGCVTL	7	0.468
**SVM**	4	RCPAMGPAT	55	0.428

**ALLELE: HLA-DRB1*0405**				
Threshold 1.5 as cutoff score				
Prediction method	Rank	Sequence	Residue No.	Peptide Score
**SVM**	1	LTAEGKPSV	15	0.647
**SVM**	2	KLANSKVAA	46	0.643
**SVM**	3	EGKPSVDVW	18	0.638
**SVM**	4	SCETKKKAT	102	0.637

**ALLELE: HLA-DRB1*0802**				
Threshold 1.5 as cutoff score				
Prediction method	Rank	Sequence	Residue No.	Peptide Score
**SVM**	1	KLANSKVAA	46	1.111
**SVM**	2	CPAMGPATL	56	1.036
**SVM**	3	KVAARCPAM	51	0.737
**SVM**	4	EHQASTVCR	67	0.683

**ALLELE: HLA-DRB1*0901**				
Threshold 1.5 as cutoff score				
Prediction method	Rank	Sequence	Residue No.	Peptide Score
**SVM**	1	TRVSLVLEL	1	1.820
**SVM**	2	GYVYDVNKI	111	1.589
**SVM**	3	VSLVLELGG	3	1.589
**SVM**	4	KPSVDVWLD	20	1.580

**ALLELE: HLA-DRB1*1101**				
Threshold 1.5 as cutoff score				
Prediction method	Rank	Sequence	Residue No.	Peptide Score
**SVM**	1	GSIVACAKF	93	0.788
**SVM**	2	FGKGSIVAC	90	0.779
**SVM**	3	IVACAKFSC	95	0.683
**SVM**	4	NSKVAARCP	49	0.536

**ALLELE: HLA-DRB1*1302**				
Threshold 1.5 as cutoff score				
Prediction method	Rank	Sequence	Residue No.	Peptide Score

**SVM**	1	KVAARCPAM	51	0.889
**SVM**	2	IVACAKFSC	95	0.796
**SVM**	3	KLANSKVAA	46	0.791
**SVM**	4	HQENPAKTR	31	0.758

**ALLELE: HLA-DRB1*1501**				
Threshold 1.5 as cutoff score				
Prediction method	Rank	Sequence	Residue No.	Peptide Score
**SVM**	1	ETKKKATGY	104	0.911
**SVM**	2	KVAARCPAM	51	0.670
**SVM**	3	VCRRDQSDR	73	0.647
**SVM**	4	VYDVNKITY	113	0.646

**ALLELE: I-Ab**				
Threshold 1.5 as cutoff score				
Prediction method	Rank	Sequence	Residue No.	Peptide Score
**SVM**	1	PATLPEEHQ	61	0.910
**SVM**	2	IHQENPAKT	30	0.895
**SVM**	3	GYVYDVNKI	111	0.682
**SVM**	4	VWLDDIHQE	25	0.657

**ALLELE: I-Ad**				
Threshold 1.5 as cutoff score				
Prediction method	Rank	Sequence	Residue No.	Peptide Score
**SVM**	1	FGKGSIVAC	90	0.681
**SVM**	2	LANSKVAAR	47	0.492
**SVM**	3	SDRGWGNHC	79	0.484
**SVM**	4	LELGGCVTL	7	0.467

**ALLELE: I-Ag7**				
Threshold 1.5 as cutoff score				
Prediction method	Rank	Sequence	Residue No.	Peptide Score
**SVM**	1	SKVAARCPA	50	1.922
**SVM**	2	RCPAMGPAT	55	1.697
**SVM**	3	ENPAKTREY	33	1.652
**SVM**	4	SIVACAKFS	94	1.474

**ALLELE: I-As**				
Threshold 1.5 as cutoff score				
Prediction method	Rank	Sequence	Residue No.	Peptide Score
**SVM**	1	VSLVLELGG	3	0.104
**SVM**	2	TAEGKPSVD	16	-0.093
**SVM**	3	TRVSLVLEL	1	-0.111
**SVM**	4	CLHAKLANS	42	-0.114

**ALLELE: RT1.B**				
Threshold 1.5 as cutoff score				
Prediction method	Rank	Sequence	Residue No.	Peptide Score
**SVM**	1	PEEHQASTV	65	0.707
**SVM**	2	TREYCLHAK	38	0.565

**SVM**	3	MGPATLPEE	59	0.442
**SVM**	4	KKKATGYVY	106	0.359

>> created at Bioinformatics Centre, Institute of Microbial Technology, Chandigarh

MHC2Pred

SVM based method for prediction of promiscuous MHC class II binders

Antigen Name	Untitled
Scanned on	Mon Apr 29 02:53:34 2019
Length of input sequence	115 amino acids
Number of nonamers from input sequence	107
Threshold setting	1.5
Number of alleles in query	42

ALLELE: HLA-DR1				
Threshold 1.5 as cutoff score				
Prediction method	Rank	Sequence	Residue No.	Peptide Score
**SVM**	1	KVEPHTGDY	1	0.463
**SVM**	2	PAQTVVLEL	53	0.384
**SVM**	3	HSNRKTASF	16	0.186
**SVM**	4	PWRHEGAQE	85	0.169

ALLELE: HLA-DR4				
Threshold 1.5 as cutoff score				
Prediction method	Rank	Sequence	Residue No.	Peptide Score
**SVM**	1	KTASFTTQS	20	1.028
**SVM**	2	ISLTCRVTS	41	0.712
**SVM**	3	NRKTASFTT	18	0.595
**SVM**	4	LVEFGEPHA	100	0.526

ALLELE: HLA-DR9				
Threshold 1.5 as cutoff score				
Prediction method	Rank	Sequence	Residue No.	Peptide Score
**SVM**	1	TLGDYGDIS	34	1.356
**SVM**	2	SFTTQSEKT	23	1.350
**SVM**	3	TCRVTSGVD	44	1.328
**SVM**	4	RDWFEDLSL	76	1.326

ALLELE: HLA-DR11				
Threshold 1.5 as cutoff score				
Prediction method	Rank	Sequence	Residue No.	Peptide Score
**SVM**	1	DLSLPWRHE	81	0.128
**SVM**	2	HLPKAWQVH	67	0.123
**SVM**	3	SLTCRVTSG	42	0.118
**SVM**	4	VTSGVDPAQ	47	0.106

ALLELE: HLA-DR2				
Threshold 1.5 as cutoff score				
Prediction method	Rank	Sequence	Residue No.	Peptide Score
**SVM**	1	QSEKTILTL	27	0.972
**SVM**	2	KTILTLGDY	30	0.931

SVM	3	HLPKAWQVH	67	0.682
SVM	4	VVLELDKTA	57	0.576

ALLELE: HLA-DR3				
Threshold 1.5 as cutoff score				
Prediction method	Rank	Sequence	Residue No.	Peptide Score
SVM	1	FEDLSLPWR	79	1.168
SVM	2	PKAWQVHRD	69	0.819
SVM	3	SEKTILTLG	28	0.738
SVM	4	DLSLPWRHE	81	0.716

ALLELE: HLA-DR5				
Threshold 1.5 as cutoff score				
Prediction method	Rank	Sequence	Residue No.	Peptide Score
SVM	1	TVVLELDKT	56	0.211
SVM	2	KTAEHLPKA	63	-0.059
SVM	3	LPKAWQVHR	68	-0.105
SVM	4	LSLPWRHEG	82	-0.114

ALLELE: HLA-DR7				
Threshold 1.5 as cutoff score				
Prediction method	Rank	Sequence	Residue No.	Peptide Score
SVM	1	VDPAQTVVL	51	0.937
SVM	2	QEWNHADRL	92	0.463
SVM	3	LPKAWQVHR	68	0.351
SVM	4	HAVKMDIFN	107	0.252

ALLELE: HLA-DR8				
Threshold 1.5 as cutoff score				
Prediction method	Rank	Sequence	Residue No.	Peptide Score
SVM	1	DISLTCRVT	40	0.932
SVM	2	LDKTAEHLP	61	0.539
SVM	3	RHEGAQEWN	87	0.480
SVM	4	TTQSEKTIL	25	0.453

ALLELE: HLA-DR13				
Threshold 1.5 as cutoff score				
Prediction method	Rank	Sequence	Residue No.	Peptide Score
SVM	1	VEFGEPHAV	101	1.723
SVM	2	RLVEFGEPH	99	0.799
SVM	3	HADRLVEFG	96	0.573
SVM	4	HAVKMDIFN	107	0.547

ALLELE: HLA-DR15				
Threshold 1.5 as cutoff score				
Prediction method	Rank	Sequence	Residue No.	Peptide Score
SVM	1	SNRKTASFT	17	1.205
SVM	2	LPKAWQVHR	68	0.839
SVM	3	YLAANESHS	9	0.794
SVM	4	ESHSNRKTA	14	0.790

ALLELE: HLA-DR51				
Threshold 1.5 as cutoff score				
Prediction method	Rank	Sequence	Residue No.	Peptide Score
**SVM**	1	YLAANESHS	9	0.751
**SVM**	2	QSEKTILTL	27	0.480
**SVM**	3	FGEPHAVKM	103	0.303
**SVM**	4	GDYLAANES	7	0.290

ALLELE: HLA-DR52				
Threshold 1.5 as cutoff score				
Prediction method	Rank	Sequence	Residue No.	Peptide Score
**SVM**	1	GVDPAQTVV	50	1.104
**SVM**	2	GDISLTCRV	39	0.933
**SVM**	3	ELDKTAEHL	60	0.816
**SVM**	4	YGDISLTCR	38	0.789

ALLELE: HLA-DR53				
Threshold 1.5 as cutoff score				
Prediction method	Rank	Sequence	Residue No.	Peptide Score
**SVM**	1	GEPHAVKMD	104	0.807
**SVM**	2	TVVLELDKT	56	0.756
**SVM**	3	HLPKAWQVH	67	0.739
**SVM**	4	AEHLPKAWQ	65	0.617

ALLELE: HLA-DQ2				
Threshold 1.5 as cutoff score				
Prediction method	Rank	Sequence	Residue No.	Peptide Score
**SVM**	1	VVLELDKTA	57	0.403
**SVM**	2	EDLSLPWRH	80	0.211
**SVM**	3	LELDKTAEH	59	0.196
**SVM**	4	LVEFGEPHA	100	0.186

ALLELE: HLA-DQ4				
Threshold 1.5 as cutoff score				
Prediction method	Rank	Sequence	Residue No.	Peptide Score
**SVM**	1	YLAANESHS	9	0.862
**SVM**	2	DWFEDLSLP	77	0.847
**SVM**	3	HRDWFEDLS	75	0.847
**SVM**	4	RDWFEDLSL	76	0.816

ALLELE: HLA-DQ6				
Threshold 1.5 as cutoff score				
Prediction method	Rank	Sequence	Residue No.	Peptide Score
**SVM**	1	SHSNRKTAS	15	0.801
**SVM**	2	LGDYGDISL	35	0.651
**SVM**	3	EDLSLPWRH	80	0.639
**SVM**	4	NESHSNRKT	13	0.542

ALLELE: HLA-DQ7				
Threshold 1.5 as cutoff score				
Prediction method	Rank	Sequence	Residue No.	Peptide Score
**SVM**	1	RKTASFTTQ	19	2.150
**SVM**	2	GVDPAQTVV	50	1.445
**SVM**	3	YGDISLTCR	38	1.116
**SVM**	4	RVTSGVDPA	46	1.039

ALLELE: HLA-DQ8				
Threshold 1.5 as cutoff score				
Prediction method	Rank	Sequence	Residue No.	Peptide Score
**SVM**	1	RDWFEDLSL	76	1.797
**SVM**	2	TVVLELDKT	56	1.732
**SVM**	3	VLELDKTAE	58	1.504
**SVM**	4	RKTASFTTQ	19	1.393

ALLELE: HLA-DQA1*0501				
Threshold 1.5 as cutoff score				
Prediction method	Rank	Sequence	Residue No.	Peptide Score
**SVM**	1	WRHEGAQEW	86	0.350
**SVM**	2	TSGVDPAQT	48	0.324
**SVM**	3	EGAQEWNHA	89	0.273
**SVM**	4	EHLPKAWQV	66	0.261

ALLELE: HLA-DQB1*02				
Threshold 1.5 as cutoff score				
Prediction method	Rank	Sequence	Residue No.	Peptide Score
**SVM**	1	QEWNHADRL	92	0.114
**SVM**	2	GAQEWNHAD	90	0.097
**SVM**	3	TAEHLPKAW	64	0.027
**SVM**	4	SEKTILTLG	28	0.017

ALLELE: HLA-DQB1*0201				
Threshold 1.5 as cutoff score				
Prediction method	Rank	Sequence	Residue No.	Peptide Score
**SVM**	1	WRHEGAQEW	86	0.245
**SVM**	2	QEWNHADRL	92	0.172
**SVM**	3	TAEHLPKAW	64	0.171
**SVM**	4	EPHAVKMDI	105	0.147

ALLELE: HLA-DQB1*03				
Threshold 1.5 as cutoff score				
Prediction method	Rank	Sequence	Residue No.	Peptide Score
**SVM**	1	HADRLVEFG	96	0.959
**SVM**	2	HAVKMDIFN	107	0.888
**SVM**	3	TAEHLPKAW	64	0.821
**SVM**	4	PAQTVVLEL	53	0.795

ALLELE: HLA-DQB1*0301				
Threshold 1.5 as cutoff score				
Prediction method	Rank	Sequence	Residue No.	Peptide Score
**SVM**	1	SHSNRKTAS	15	1.038
**SVM**	2	PKAWQVHRD	69	0.897
**SVM**	3	KTASFTTQS	20	0.806
**SVM**	4	WFEDLSLPW	78	0.751

ALLELE: HLA-DQB1*0302				
Threshold 1.5 as cutoff score				
Prediction method	Rank	Sequence	Residue No.	Peptide Score
**SVM**	1	NHADRLVEF	95	0.362
**SVM**	2	GAQEWNHAD	90	0.262
**SVM**	3	DPAQTVVLE	52	0.194
**SVM**	4	SNRKTASFT	17	0.142

ALLELE: HLA-DRB1*0101				
Threshold 1.5 as cutoff score				
Prediction method	Rank	Sequence	Residue No.	Peptide Score
**SVM**	1	NRKTASFTT	18	1.025
**SVM**	2	GVDPAQTVV	50	0.996
**SVM**	3	YGDISLTCR	38	0.750
**SVM**	4	LPKAWQVHR	68	0.671

ALLELE: HLA-DRB4*0101				
Threshold 1.5 as cutoff score				
Prediction method	Rank	Sequence	Residue No.	Peptide Score
**SVM**	1	LSLPWRHEG	82	0.803
**SVM**	2	EDLSLPWRH	80	0.609
**SVM**	3	DLSLPWRHE	81	0.544
**SVM**	4	FEDLSLPWR	79	0.471

ALLELE: HLA-DRB5*0101				
Threshold 1.5 as cutoff score				
Prediction method	Rank	Sequence	Residue No.	Peptide Score
**SVM**	1	AWQVHRDWF	71	0.836
**SVM**	2	YLAANESHS	9	0.714
**SVM**	3	DKTAEHLPK	62	0.626
**SVM**	4	YGDISLTCR	38	0.444

ALLELE: HLA-DRB1*0301				
Threshold 1.5 as cutoff score				
Prediction method	Rank	Sequence	Residue No.	Peptide Score
**SVM**	1	SEKTILTLG	28	1.087
**SVM**	2	EKTILTLGD	29	0.888
**SVM**	3	ILTLGDYGD	32	0.857
**SVM**	4	LDKTAEHLP	61	0.701

ALLELE: HLA-DRB1*0401
Threshold 1.5 as cutoff score

Prediction method	Rank	Sequence	Residue No.	Peptide Score
**SVM**	1	NRKTASFTT	18	0.775
**SVM**	2	DKTAEHLPK	62	0.690
**SVM**	3	LELDKTAEH	59	0.638
**SVM**	4	GVDPAQTVV	50	0.609

**ALLELE: HLA-DRB1*0404**				
Threshold 1.5 as cutoff score				
Prediction method	Rank	Sequence	Residue No.	Peptide Score
**SVM**	1	VLELDKTAE	58	0.693
**SVM**	2	TVVLELDKT	56	0.481
**SVM**	3	RLVEFGEPH	99	0.435
**SVM**	4	LPWRHEGAQ	84	0.358

**ALLELE: HLA-DRB1*0405**				
Threshold 1.5 as cutoff score				
Prediction method	Rank	Sequence	Residue No.	Peptide Score
**SVM**	1	SFTTQSEKT	23	0.661
**SVM**	2	SHSNRKTAS	15	0.644
**SVM**	3	ISLTCRVTS	41	0.643
**SVM**	4	TVVLELDKT	56	0.638

**ALLELE: HLA-DRB1*0802**				
Threshold 1.5 as cutoff score				
Prediction method	Rank	Sequence	Residue No.	Peptide Score
**SVM**	1	DPAQTVVLE	52	0.816
**SVM**	2	VDPAQTVVL	51	0.753
**SVM**	3	KAWQVHRDW	70	0.672
**SVM**	4	KTASFTTQS	20	0.570

**ALLELE: HLA-DRB1*0901**				
Threshold 1.5 as cutoff score				
Prediction method	Rank	Sequence	Residue No.	Peptide Score
**SVM**	1	EKTILTLGD	29	1.657
**SVM**	2	SLTCRVTSG	42	1.539
**SVM**	3	QTVVLELDK	55	1.497
**SVM**	4	ILTLGDYGD	32	1.472

**ALLELE: HLA-DRB1*1101**				
Threshold 1.5 as cutoff score				
Prediction method	Rank	Sequence	Residue No.	Peptide Score
**SVM**	1	PHAVKMDIF	106	0.712
**SVM**	2	NHADRLVEF	95	0.674
**SVM**	3	LAANESHSN	10	0.651
**SVM**	4	NRKTASFTT	18	0.449

**ALLELE: HLA-DRB1*1302**				
Threshold 1.5 as cutoff score				
Prediction method	Rank	Sequence	Residue No.	Peptide Score

**SVM**	1	AQEWNHADR	91	0.556
**SVM**	2	CRVTSGVDP	45	0.464
**SVM**	3	TQSEKTILT	26	0.370
**SVM**	4	KVEPHTGDY	1	0.344

**ALLELE: HLA-DRB1*1501**				
Threshold 1.5 as cutoff score				
Prediction method	Rank	Sequence	Residue No.	Peptide Score
**SVM**	1	SNRKTASFT	17	0.816
**SVM**	2	NESHSNRKT	13	0.698
**SVM**	3	YLAANESHS	9	0.692
**SVM**	4	YGDISLTCR	38	0.637

**ALLELE: I-Ab**				
Threshold 1.5 as cutoff score				
Prediction method	Rank	Sequence	Residue No.	Peptide Score
**SVM**	1	DKTAEHLPK	62	1.442
**SVM**	2	PAQTVVLEL	53	1.075
**SVM**	3	RKTASFTTQ	19	1.061
**SVM**	4	WNHADRLVE	94	0.845

**ALLELE: I-Ad**				
Threshold 1.5 as cutoff score				
Prediction method	Rank	Sequence	Residue No.	Peptide Score
**SVM**	1	HADRLVEFG	96	0.678
**SVM**	2	GAQEWNHAD	90	0.601
**SVM**	3	PAQTVVLEL	53	0.496
**SVM**	4	TILTLGDYG	31	0.494

**ALLELE: I-Ag7**				
Threshold 1.5 as cutoff score				
Prediction method	Rank	Sequence	Residue No.	Peptide Score
**SVM**	1	GDYLAANES	7	1.466
**SVM**	2	DYLAANESH	8	1.343
**SVM**	3	EPHTGDYLA	3	1.263
**SVM**	4	TGDYLAANE	6	1.243

**ALLELE: I-As**				
Threshold 1.5 as cutoff score				
Prediction method	Rank	Sequence	Residue No.	Peptide Score
**SVM**	1	HSNRKTASF	16	0.089
**SVM**	2	TLGDYGDIS	34	0.080
**SVM**	3	LELDKTAEH	59	0.045
**SVM**	4	PHAVKMDIF	106	-0.089

**ALLELE: RT1.B**				
Threshold 1.5 as cutoff score				
Prediction method	Rank	Sequence	Residue No.	Peptide Score
**SVM**	1	FTTQSEKTI	24	1.015
**SVM**	2	TASFTTQSE	21	0.987

**SVM**	3	RKTASFTTQ	19	0.889
**SVM**	4	SFTTQSEKT	23	0.711

>> created at Bioinformatics Centre, Institute of Microbial Technology, Chandigarh

MHC2Pred

SVM based method for prediction of promiscuous MHC class II binders

Antigen Name	Untitled
Scanned on	Mon Apr 29 02:54:06 2019
Length of input sequence	149 amino acids
Number of nonamers from input sequence	141
Threshold setting	1.5
Number of alleles in query	42

**ALLELE: HLA-DR1**				
Threshold 1.5 as cutoff score				
Prediction method	Rank	Sequence	Residue No.	Peptide Score
**SVM**	1	IPVRAVAHG	83	0.959
**SVM**	2	VVMEVTYTG	69	0.485
**SVM**	3	NPSMETTGG	104	0.286
**SVM**	4	KLKMKGMTY	39	0.253

**ALLELE: HLA-DR4**				
Threshold 1.5 as cutoff score				
Prediction method	Rank	Sequence	Residue No.	Peptide Score
**SVM**	1	NVASLITPN	96	1.648
**SVM**	2	NVNVASLIT	94	0.978
**SVM**	3	VRAVAHGEP	85	0.824
**SVM**	4	VMEVTYTGS	70	0.780

**ALLELE: HLA-DR9**				
Threshold 1.5 as cutoff score				
Prediction method	Rank	Sequence	Residue No.	Peptide Score
**SVM**	1	TYTVCEGSK	46	1.689
**SVM**	2	METTGGGFV	107	1.671
**SVM**	3	HQWFQKGST	133	1.627
**SVM**	4	KGSTIGRVL	138	1.555

**ALLELE: HLA-DR11**				
Threshold 1.5 as cutoff score				
Prediction method	Rank	Sequence	Residue No.	Peptide Score
**SVM**	1	SLAGVPVAN	10	0.206
**SVM**	2	GFVELQLPP	113	0.157
**SVM**	3	SLITPNPSM	99	0.134
**SVM**	4	IYVGELSHQ	126	0.120

**ALLELE: HLA-DR2**				
Threshold 1.5 as cutoff score				
Prediction method	Rank	Sequence	Residue No.	Peptide Score
**SVM**	1	EGSKYHLQS	20	0.942
**SVM**	2	ILLKSLAGV	6	0.912

SVM	3	DQTGILLKS	2	0.699
SVM	4	PGDNIIYVG	121	0.667

ALLELE: HLA-DR3				
Threshold 1.5 as cutoff score				
Prediction method	Rank	Sequence	Residue No.	Peptide Score
SVM	1	IEGSKYHLQ	19	1.400
SVM	2	TIGRVLEKT	141	1.341
SVM	3	PGDNIIYVG	121	1.071
SVM	4	TGGGFVELQ	110	1.038

ALLELE: HLA-DR5				
Threshold 1.5 as cutoff score				
Prediction method	Rank	Sequence	Residue No.	Peptide Score
SVM	1	TCDVGLEKL	32	0.146
SVM	2	IYVGELSHQ	126	0.123
SVM	3	VGLEKLKMK	35	0.104
SVM	4	LSHQWFQKG	131	0.043

ALLELE: HLA-DR7				
Threshold 1.5 as cutoff score				
Prediction method	Rank	Sequence	Residue No.	Peptide Score
SVM	1	VNVASLITP	95	0.552
SVM	2	FQKGSTIGR	136	0.537
SVM	3	KSLAGVPVA	9	0.503
SVM	4	LITPNPSME	100	0.431

ALLELE: HLA-DR8				
Threshold 1.5 as cutoff score				
Prediction method	Rank	Sequence	Residue No.	Peptide Score
SVM	1	LKSLAGVPV	8	0.659
SVM	2	KGSTIGRVL	138	0.649
SVM	3	LQSGHVTCD	26	0.602
SVM	4	YHLQSGHVT	24	0.362

ALLELE: HLA-DR13				
Threshold 1.5 as cutoff score				
Prediction method	Rank	Sequence	Residue No.	Peptide Score
SVM	1	KPCRIPVRA	79	1.781
SVM	2	SLAGVPVAN	10	1.476
SVM	3	RIPVRAVAH	82	1.066
SVM	4	PPGDNIIYV	120	0.929

ALLELE: HLA-DR15				
Threshold 1.5 as cutoff score				
Prediction method	Rank	Sequence	Residue No.	Peptide Score
SVM	1	SLAGVPVAN	10	1.345
SVM	2	SLITPNPSM	99	1.327
SVM	3	KLKMKGMTY	39	1.125
SVM	4	KMKGMTYTV	41	1.041

ALLELE: HLA-DR51				
Threshold 1.5 as cutoff score				
Prediction method	Rank	Sequence	Residue No.	Peptide Score
**SVM**	1	NVASLITPN	96	0.626
**SVM**	2	ASLITPNPS	98	0.468
**SVM**	3	DQTGILLKS	2	0.421
**SVM**	4	NIEGSKYHL	18	0.360

ALLELE: HLA-DR52				
Threshold 1.5 as cutoff score				
Prediction method	Rank	Sequence	Residue No.	Peptide Score
**SVM**	1	GLEKLKMKG	36	1.172
**SVM**	2	CDVGLEKLK	33	0.945
**SVM**	3	VGLEKLKMK	35	0.812
**SVM**	4	KGSTIGRVL	138	0.775

ALLELE: HLA-DR53				
Threshold 1.5 as cutoff score				
Prediction method	Rank	Sequence	Residue No.	Peptide Score
**SVM**	1	QLPPGDNII	118	1.035
**SVM**	2	LEKLKMKGM	37	0.888
**SVM**	3	IEGSKYHLQ	19	0.866
**SVM**	4	VELQLPPGD	115	0.675

ALLELE: HLA-DQ2				
Threshold 1.5 as cutoff score				
Prediction method	Rank	Sequence	Residue No.	Peptide Score
**SVM**	1	VELQLPPGD	115	0.612
**SVM**	2	LAGVPVANI	11	0.453
**SVM**	3	LQLPPGDNI	117	0.276
**SVM**	4	VGLEKLKMK	35	0.175

ALLELE: HLA-DQ4				
Threshold 1.5 as cutoff score				
Prediction method	Rank	Sequence	Residue No.	Peptide Score
**SVM**	1	PNVNVASLI	93	1.029
**SVM**	2	IYVGELSHQ	126	0.884
**SVM**	3	VNVASLITP	95	0.859
**SVM**	4	RIPVRAVAH	82	0.802

ALLELE: HLA-DQ6				
Threshold 1.5 as cutoff score				
Prediction method	Rank	Sequence	Residue No.	Peptide Score
**SVM**	1	SKPCRIPVR	78	0.936
**SVM**	2	VELQLPPGD	115	0.894
**SVM**	3	EKLKMKGMT	38	0.836
**SVM**	4	EPNVNVASL	92	0.715

ALLELE: HLA-DQ7				
Threshold 1.5 as cutoff score				
Prediction method	Rank	Sequence	Residue No.	Peptide Score
**SVM**	1	PCRIPVRAV	80	1.714
**SVM**	2	GGGFVELQL	111	1.364
**SVM**	3	METTGGGFV	107	1.303
**SVM**	4	PTDSGHDTV	61	1.258

ALLELE: HLA-DQ8				
Threshold 1.5 as cutoff score				
Prediction method	Rank	Sequence	Residue No.	Peptide Score
**SVM**	1	MKGMTYTVC	42	1.368
**SVM**	2	DVGLEKLKM	34	1.209
**SVM**	3	TVVMEVTYT	68	1.014
**SVM**	4	SKPCRIPVR	78	0.900

ALLELE: HLA-DQA1*0501				
Threshold 1.5 as cutoff score				
Prediction method	Rank	Sequence	Residue No.	Peptide Score
**SVM**	1	GFVELQLPP	113	0.344
**SVM**	2	TGILLKSLA	4	0.282
**SVM**	3	YVGELSHQW	127	0.254
**SVM**	4	QWFQKGSTI	134	0.246

ALLELE: HLA-DQB1*02				
Threshold 1.5 as cutoff score				
Prediction method	Rank	Sequence	Residue No.	Peptide Score
**SVM**	1	SLAGVPVAN	10	0.212
**SVM**	2	DVGLEKLKM	34	0.162
**SVM**	3	YVGELSHQW	127	0.138
**SVM**	4	GGFVELQLP	112	0.092

ALLELE: HLA-DQB1*0201				
Threshold 1.5 as cutoff score				
Prediction method	Rank	Sequence	Residue No.	Peptide Score
**SVM**	1	TPNPSMETT	102	0.403
**SVM**	2	TGGGFVELQ	110	0.342
**SVM**	3	TIGRVLEKT	141	0.319
**SVM**	4	TGILLKSLA	4	0.317

ALLELE: HLA-DQB1*03				
Threshold 1.5 as cutoff score				
Prediction method	Rank	Sequence	Residue No.	Peptide Score
**SVM**	1	ELSHQWFQK	130	0.880
**SVM**	2	QLPPGDNII	118	0.798
**SVM**	3	VASLITPNP	97	0.763
**SVM**	4	LPPGDNIIY	119	0.744

ALLELE: HLA-DQB1*0301				
Threshold 1.5 as cutoff score				
Prediction method	Rank	Sequence	Residue No.	Peptide Score
**SVM**	1	GILLKSLAG	5	1.047
**SVM**	2	ASLITPNPS	98	0.987
**SVM**	3	SLAGVPVAN	10	0.963
**SVM**	4	SKFAWKRPP	53	0.851

ALLELE: HLA-DQB1*0302				
Threshold 1.5 as cutoff score				
Prediction method	Rank	Sequence	Residue No.	Peptide Score
**SVM**	1	GGFVELQLP	112	0.448
**SVM**	2	TCDVGLEKL	32	0.301
**SVM**	3	GELSHQWFQ	129	0.231
**SVM**	4	TIGRVLEKT	141	0.203

ALLELE: HLA-DRB1*0101				
Threshold 1.5 as cutoff score				
Prediction method	Rank	Sequence	Residue No.	Peptide Score
**SVM**	1	NVNVASLIT	94	1.683
**SVM**	2	TVVMEVTYT	68	1.101
**SVM**	3	LQSGHVTCD	26	1.019
**SVM**	4	AWKRPPTDS	56	0.891

ALLELE: HLA-DRB4*0101				
Threshold 1.5 as cutoff score				
Prediction method	Rank	Sequence	Residue No.	Peptide Score
**SVM**	1	VELQLPPGD	115	1.155
**SVM**	2	TGSKPCRIP	76	1.077
**SVM**	3	FVELQLPPG	114	0.684
**SVM**	4	VGLEKLKMK	35	0.636

ALLELE: HLA-DRB5*0101				
Threshold 1.5 as cutoff score				
Prediction method	Rank	Sequence	Residue No.	Peptide Score
**SVM**	1	AWKRPPTDS	56	1.317
**SVM**	2	NVASLITPN	96	0.792
**SVM**	3	TYTVCEGSK	46	0.535
**SVM**	4	GFVELQLPP	113	0.513

ALLELE: HLA-DRB1*0301				
Threshold 1.5 as cutoff score				
Prediction method	Rank	Sequence	Residue No.	Peptide Score
**SVM**	1	TGILLKSLA	4	0.974
**SVM**	2	GDNIIYVGE	122	0.649
**SVM**	3	GSTIGRVLE	139	0.642
**SVM**	4	MKGMTYTVC	42	0.618

ALLELE: HLA-DRB1*0401
Threshold 1.5 as cutoff score

Prediction method	Rank	Sequence	Residue No.	Peptide Score
SVM	1	ILLKSLAGV	6	0.861
SVM	2	NPSMETTGG	104	0.766
SVM	3	SKFAWKRPP	53	0.757
SVM	4	FAWKRPPTD	55	0.756

ALLELE: HLA-DRB1*0404				
Threshold 1.5 as cutoff score				
Prediction method	Rank	Sequence	Residue No.	Peptide Score
SVM	1	TVVMEVTYT	68	0.494
SVM	2	VCEGSKFAW	49	0.481
SVM	3	ELQLPPGDN	116	0.465
SVM	4	IYVGELSHQ	126	0.449

ALLELE: HLA-DRB1*0405				
Threshold 1.5 as cutoff score				
Prediction method	Rank	Sequence	Residue No.	Peptide Score
SVM	1	GDQTGILLK	1	0.661
SVM	2	NVASLITPN	96	0.646
SVM	3	NVNVASLIT	94	0.645
SVM	4	ITPNPSMET	101	0.643

ALLELE: HLA-DRB1*0802				
Threshold 1.5 as cutoff score				
Prediction method	Rank	Sequence	Residue No.	Peptide Score
SVM	1	KLKMKGMTY	39	0.970
SVM	2	HLQSGHVTC	25	0.960
SVM	3	KFAWKRPPT	54	0.696
SVM	4	VNVASLITP	95	0.677

ALLELE: HLA-DRB1*0901				
Threshold 1.5 as cutoff score				
Prediction method	Rank	Sequence	Residue No.	Peptide Score
SVM	1	GGGFVELQL	111	1.679
SVM	2	KGSTIGRVL	138	1.668
SVM	3	VELQLPPGD	115	1.666
SVM	4	STIGRVLEK	140	1.658

ALLELE: HLA-DRB1*1101				
Threshold 1.5 as cutoff score				
Prediction method	Rank	Sequence	Residue No.	Peptide Score
SVM	1	GSKFAWKRP	52	1.436
SVM	2	NVASLITPN	96	0.749
SVM	3	VRAVAHGEP	85	0.614
SVM	4	LKSLAGVPV	8	0.570

ALLELE: HLA-DRB1*1302				
Threshold 1.5 as cutoff score				
Prediction method	Rank	Sequence	Residue No.	Peptide Score

**SVM**	1	PVANIEGSK	15	0.779
**SVM**	2	EVTYTGSKP	72	0.619
**SVM**	3	KFAWKRPPT	54	0.573
**SVM**	4	NVNVASLIT	94	0.554

**ALLELE: HLA-DRB1*1501**				
Threshold 1.5 as cutoff score				
Prediction method	Rank	Sequence	Residue No.	Peptide Score
**SVM**	1	VNVASLITP	95	1.232
**SVM**	2	KFAWKRPPT	54	1.165
**SVM**	3	EGSKFAWKR	51	0.873
**SVM**	4	KLKMKGMTY	39	0.731

**ALLELE: I-Ab**				
Threshold 1.5 as cutoff score				
Prediction method	Rank	Sequence	Residue No.	Peptide Score
**SVM**	1	FAWKRPPTD	55	0.756
**SVM**	2	TVVMEVTYT	68	0.742
**SVM**	3	KFAWKRPPT	54	0.674
**SVM**	4	EKLKMKGMT	38	0.650

**ALLELE: I-Ad**				
Threshold 1.5 as cutoff score				
Prediction method	Rank	Sequence	Residue No.	Peptide Score
**SVM**	1	TTGGGFVEL	109	0.608
**SVM**	2	GEPNVNVAS	91	0.464
**SVM**	3	FQKGSTIGR	136	0.462
**SVM**	4	GDNIIYVGE	122	0.450

**ALLELE: I-Ag7**				
Threshold 1.5 as cutoff score				
Prediction method	Rank	Sequence	Residue No.	Peptide Score
**SVM**	1	SKFAWKRPP	53	1.622
**SVM**	2	GVPVANIEG	13	1.602
**SVM**	3	VPVANIEGS	14	1.412
**SVM**	4	EPNVNVASL	92	1.406

**ALLELE: I-As**				
Threshold 1.5 as cutoff score				
Prediction method	Rank	Sequence	Residue No.	Peptide Score
**SVM**	1	GILLKSLAG	5	-0.058
**SVM**	2	TGILLKSLA	4	-0.079
**SVM**	3	MKGMTYTVC	42	-0.105
**SVM**	4	VMEVTYTGS	70	-0.129

**ALLELE: RT1.B**				
Threshold 1.5 as cutoff score				
Prediction method	Rank	Sequence	Residue No.	Peptide Score
**SVM**	1	TTGGGFVEL	109	0.726
**SVM**	2	TGGGFVELQ	110	0.579

**SVM**	3	IEGSKYHLQ	19	0.428
**SVM**	4	QTGILLKSL	3	0.421

>> created at Bioinformatics Centre, Institute of Microbial Technology, Chandigarh

Supplementary Table 8:
BLAST analysis of top ranked epitopes against UniProt-human database

100 80 60 40 20 0
Identity %

Edit and resubmit Order by: Score

Overview

Entry	Protein names	Match hit	Ider
H7C1N4	Protein shisa-7 (Homo sapiens)		4
O00584-2	**Isoform 2 of Ribonuclease T2** (Homo sapiens)		2
A0A087WWI1	Ribonuclease T2 (Homo sapiens)		2
Q8TCU1	Extra-cellular ribonuclease (Homo sapiens)		2

Alignments

	Alignment overview	Info	Sta
Query: B20190506674680338 1A1F0E0DB47453E0216320D01E910V			
H7C1N4_HUMAN - Protein shisa-7 Homo sapiens (Human) - View alignment	E-value: 2e-1<br>Score: 81<br>Ident.: 42.9%		
RNT2_HUMAN - Isoform 2 of Ribonuclease T2 Homo sapiens (Human) - View alignment	E-value: 7.7e-1<br>Score: 75<br>Ident.: 28.8%		
A0A087WWI1_HUMAN - Ribonuclease T2 Homo sapiens (Human) - View alignment	E-value: 2.1e0<br>Score: 75<br>Ident.: 28.8%		
Q8TCU1_HUMAN - Extra-cellular ribonuclease Homo sapiens (Human) - View alignment	E-value: 2.1e0<br>Score: 75<br>Ident.: 28.8%		

Job information

Query sequence[i]

KAWQVHRDW
KTILTLGDY
KTAEHLPKAW
STIGRVLEK
DYGDISLTCR
KTAEHLPKAW
KPCRIPVRAV
ASFTTQSEK
KAWQVHRDW
VTCDVGLEK
YVYDVNKITY
KTILTLGDY
REYCLHAKL
LELGGCVTL
KVEPHTGDY
LPPGDNIIY
ETKKKATGY
KTAEHLPKAW
YVYDVNKITY
IYVGELSHQW
KAWQVHRDWFEDLSL
KAWQVHRDW
PKAWQVHRD
LPKAWQVHR
RDWFEDLSL

```
AWQVHRDWFEDLSLP
AWQVHRDWF
PKAWQVHRD
DWFEDLSLP
WQVHRDWFEDLSLPW
AWQVHRDWF
WFEDLSLPW
FEDLSLPWR
QVHRDWFEDLSLPWR
AWQVHRDWF
VHRDWFEDLSLPWRH
EDLSLPWRH
KPSVDVWLDDIHQEN
WLDDIHQEN
AQTVVLELDKTAEH
ASTVCRRDQSDRGW
DKTAEHLPKAWQVH
DDIHQENPAKTREY
NVNVASLITPNPSM
VGLEKLKMKGMTYT
VACAKFSCETKKKA
SLTCRVTSGVDPAQ
GVPVANIEGSKYHL
FGKGSIVACAKFSC
KRPPTDSGHDTVVM
QLPPGDNIIYVGEL
AANESHSNRKTASF
SNRKTASFTTQSEK
TGSKPCRIPVRAVA
AARCPAMGPATLPE
LDDIHQENPAKTRE
RDQSDRGWGNHCGL
KKKATGYVYDVNKI
WRHEGAQEWNHADR
LTLGDYGDISLTCR
AANESHSNRKTASF
EFGEPHAVKMDIFN
GGGFVELQLPPGDN
KFAWKRPPTDSGHD
TGSKPCRIPVRAVA
SHQWFQKGSTIGRV
EPNVNVASLITPNP
KTREYCLHAKLANS
TDSGHDTVVMEVTY
WFQKGSTIGRVLEK
TVVLELDKTAEHLP
```

Date of job execution	2019-05-06
Job identifier	B201905066746803381A1F0E0DB47453E0216320D01E910V (jobs are stored for 7 days)
Running time	25.1 seconds
Scheduled time	3,603.1 seconds
Program [i]	blastp (blastp BLASTP 2.7.1+)
Database	uniprotkb_human (Protein) generated for BLAST on Mar 31, 2019
Sequences	191,889 sequences consisting of 66,142,545 letters
Matrix [i]	blosum62
Threshold [i]	10
Filtered [i]	false
Gapped [i]	true
Maximum number of hits reported [i]	50

UniProt is an ELIXIR core data resource

Main funding by: National Institutes of Health, EMBL European Molecular Biology Laboratory, State Secretariat for Education, Research and Innovation SERI

Supplementary Table 9:
BLAST analysis of top ranked epitopes against UniProtKB database

100 80 60 40 20 0
Identity %

Edit and resubmit Order by: Score

Overview

Collapse table

Entry	Protein names	Match hit	Ide[illegible]
V9NZA4	**Genome polyprotein** (Alkhumra hemorrhagic fever v...)		3[illegible]
A0A2R2WVL1	Envelope protein (Kyasanur forest disease virus)		4[illegible]
A0A2R2WVX9	Envelope protein (Kyasanur forest disease virus)		4[illegible]
A0A218MY61	Envelope protein (Kyasanur forest disease virus)		4[illegible]
A0A218MY70	Envelope protein (Kyasanur forest disease virus)		4[illegible]
A0A218MY57	Envelope protein (Kyasanur forest disease virus)		4[illegible]
A0A218MY55	Envelope protein (Kyasanur forest disease virus)		4[illegible]
A0A218MY59	Envelope protein (Kyasanur forest disease virus)		4[illegible]
A0A3S5X0A9	Polyprotein (Kyasanur forest disease virus)		4[illegible]
A0A3S5X0L8	Polyprotein (Kyasanur forest disease virus)		4[illegible]
A0A3S5WW74	Polyprotein (Kyasanur forest disease virus)		4[illegible]
A0A3S5WYM5	Polyprotein (Kyasanur forest disease virus)		4[illegible]
A0A3S5X1M2	Polyprotein (Kyasanur forest disease virus)		4[illegible]
A0A3S5WW59	Polyprotein (Kyasanur forest disease virus)		4[illegible]
A0A3S5WYJ8	Polyprotein (Kyasanur forest disease virus)		4[illegible]
A0A3S5WYW2	Polyprotein (Kyasanur forest disease virus)		4[illegible]
A0A3S5X0X7	Polyprotein (Kyasanur forest disease virus)		4[illegible]
A0A3S5X0L3	Polyprotein (Kyasanur forest disease virus)		4[illegible]
A0A1S6VSX4	**Genome polyprotein** (Alkhumra hemorrhagic fever v...)		3[illegible]
D7RF80	**Genome polyprotein** (Kyasanur forest disease virus)		4[illegible]
A0A2R2WVS3	Envelope protein (Kyasanur forest disease virus)		4[illegible]
A0A2R2WVK2	Envelope protein (Kyasanur forest disease virus)		4[illegible]
A0A2R2WVW9	Envelope protein (Kyasanur forest disease virus)		4[illegible]
A0A2R2WVJ9	Envelope protein (Kyasanur forest disease virus)		4[illegible]
A0A2R2WVQ2	Envelope protein (Kyasanur forest disease virus)		4[illegible]
A0A2R2WWK9	Envelope protein (Kyasanur forest disease virus)		4[illegible]
A0A2R2WVK7	Envelope protein (Kyasanur forest disease virus)		4[illegible]
A0A2R2WVJ8	Envelope protein (Kyasanur forest disease virus)		4[illegible]
A0A2R2WVM2	Envelope protein (Kyasanur forest disease virus)		4[illegible]
A0A2R2WW58	Envelope protein (Kyasanur forest disease virus)		4[illegible]
A0A218MY56	Envelope protein (Kyasanur forest disease virus)		4[illegible]
A0A218MY60	Envelope protein (Kyasanur forest disease virus)		4[illegible]
A0A218MY58	Envelope protein (Kyasanur forest disease virus)		4[illegible]
A0A3S5WYV3	Polyprotein (Kyasanur forest disease virus)		4[illegible]
A0A3S5X108	Polyprotein (Kyasanur forest disease virus)		4[illegible]
A0A3S5WYL9	Polyprotein (Kyasanur forest disease virus)		4[illegible]

Entry	Protein names	Match hit	Ide
A0A3S5X2U8	Polyprotein (Kyasanur forest disease virus)		4[illegible]
A0A3S5X0A6	Polyprotein (Kyasanur forest disease virus)		4[illegible]
A0A3S5X113	Polyprotein (Kyasanur forest disease virus)		4[illegible]
A0A3S5WZT8	Polyprotein (Kyasanur forest disease virus)		4[illegible]
A0A3S5WZT3	Polyprotein (Kyasanur forest disease virus)		4[illegible]
A0A3S5WW68	Polyprotein (Kyasanur forest disease virus)		4[illegible]
A0A3S5X0X0	Polyprotein (Kyasanur forest disease virus)		4[illegible]
A0A3S5X0A0	Polyprotein (Kyasanur forest disease virus)		4[illegible]
A0A3S5WZU1	Polyprotein (Kyasanur forest disease virus)		4[illegible]
A0A3S5X1L8	Polyprotein (Kyasanur forest disease virus)		4[illegible]
A0A3S5WYV1	Polyprotein (Kyasanur forest disease virus)		4[illegible]
A0A3S5X2V1	Polyprotein (Kyasanur forest disease virus)		3[illegible]
U5IAE3	**Genome polyprotein** (Alkhumra hemorrhagic fever v...)		4[illegible]
H8Y6K5	**Genome polyprotein** (Alkhumra hemorrhagic fever v...)		4[illegible]

Alignments

	Alignment overview	Info	Sta
Query: B20190506674680338 1A1F0E0DB47453E0216320D01E8BEI			
V9NZA4_ALKV - Genome polyprotein - Alkhumra hemorrh... - View alignment 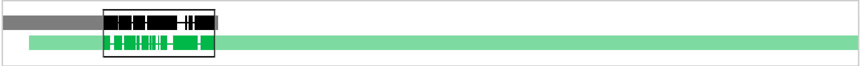	E-value: 1.7e-43<br>Score: 463<br>Ident.: 36.3%		
A0A2R2WVL1_KFDV - Envelope protein - Kyasanur forest ... - View alignment 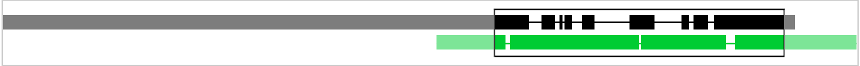	E-value: 1.1e-43<br>Score: 445<br>Ident.: 40.8%		
A0A2R2WVX9_KFDV - Envelope protein - Kyasanur forest ... - View alignment 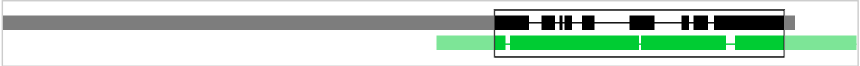	E-value: 1.1e-43<br>Score: 445<br>Ident.: 40.8%		
A0A218MY61_KFDV - Envelope protein - Kyasanur forest ... - View alignment 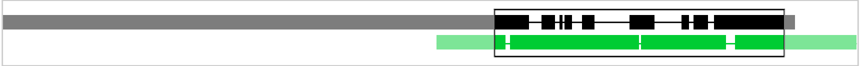	E-value: 1.1e-43<br>Score: 445<br>Ident.: 40.8%		
A0A218MY70_KFDV - Envelope protein - Kyasanur forest ... - View alignment 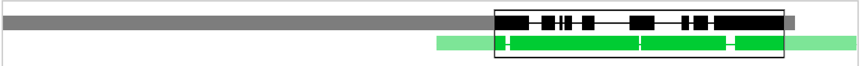	E-value: 1.1e-43<br>Score: 445<br>Ident.: 40.8%		
A0A218MY57_KFDV - Envelope protein - Kyasanur forest ... - View alignment 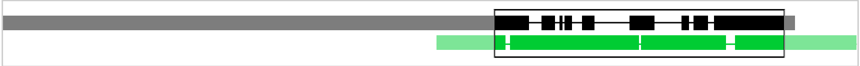	E-value: 1.1e-43<br>Score: 445<br>Ident.: 40.8%		
A0A218MY55_KFDV - Envelope protein - Kyasanur forest ... - View alignment 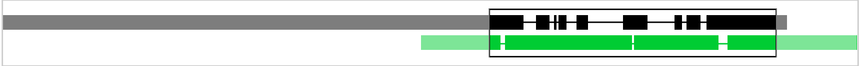	E-value: 1.8e-43<br>Score: 445<br>Ident.: 40.8%		
A0A218MY59_KFDV - Envelope protein - Kyasanur forest ... - View alignment 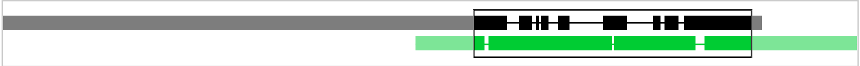	E-value: 2.7e-43<br>Score: 445<br>Ident.: 40.8%		
A0A3S5X0A9_KFDV - Polyprotein - Kyasanur forest ... - View alignment 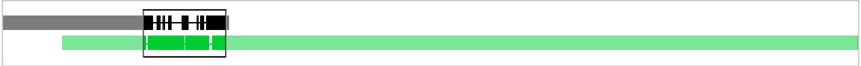	E-value: 2.7e-41<br>Score: 445<br>Ident.: 40.8%		

	Alignment overview	Info	Sta
A0A3S5X0L8_KFDV - Polyprotein - Kyasanur forest ... - View alignment 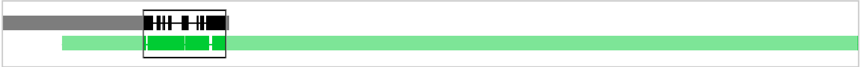	E-value: 2.7e-41<br>Score: 445<br>Ident.: 40.8%		
A0A3S5WW74_KFDV - Polyprotein - Kyasanur forest ... - View alignment 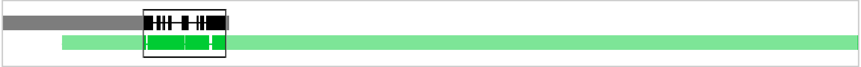	E-value: 2.7e-41<br>Score: 445<br>Ident.: 40.8%		
A0A3S5WYM5_KFDV - Polyprotein - Kyasanur forest ... - View alignment 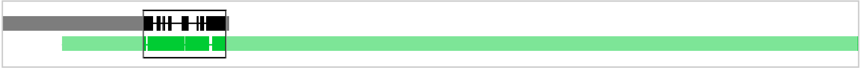	E-value: 2.7e-41<br>Score: 445<br>Ident.: 40.8%		
A0A3S5X1M2_KFDV - Polyprotein - Kyasanur forest ... - View alignment 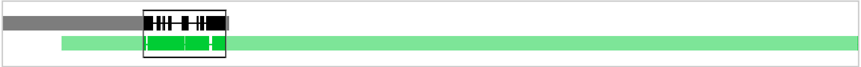	E-value: 2.7e-41<br>Score: 445<br>Ident.: 40.8%		
A0A3S5WW59_KFDV - Polyprotein - Kyasanur forest ... - View alignment 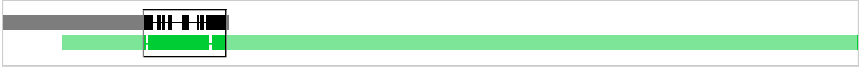	E-value: 2.7e-41<br>Score: 445<br>Ident.: 40.8%		
A0A3S5WYJ8_KFDV - Polyprotein - Kyasanur forest ... - View alignment 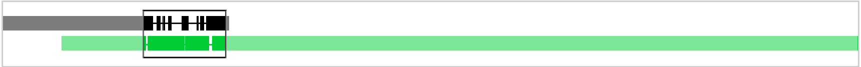	E-value: 2.7e-41<br>Score: 445<br>Ident.: 40.8%		
A0A3S5WYW2_KFDV - Polyprotein - Kyasanur forest ... - View alignment 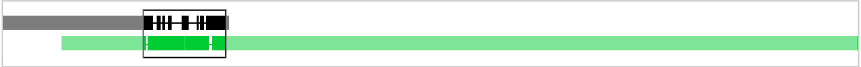	E-value: 2.7e-41<br>Score: 445<br>Ident.: 40.8%		
A0A3S5X0X7_KFDV - Polyprotein - Kyasanur forest ... - View alignment 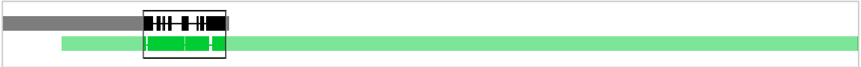	E-value: 2.7e-41<br>Score: 445<br>Ident.: 40.8%		
A0A3S5X0L3_KFDV - Polyprotein - Kyasanur forest ... - View alignment 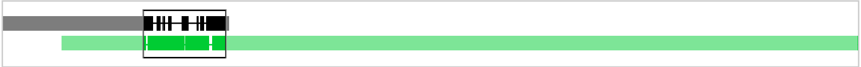	E-value: 2.7e-41<br>Score: 445<br>Ident.: 40.8%		
A0A1S6VSX4_ALKV - Genome polyprotein - Alkhumra hemorrh... - View alignment 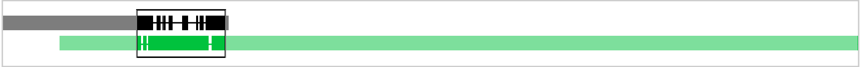	E-value: 6.4e-41<br>Score: 442<br>Ident.: 38.9%		
POLG_KFDV - Genome polyprotein - Kyasanur forest ... - View alignment 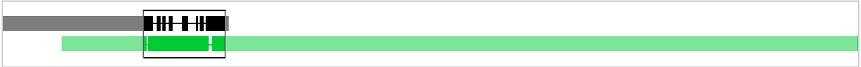	E-value: 1.1e-40<br>Score: 440<br>Ident.: 40.3%		
A0A2R2WVS3_KFDV - Envelope protein - Kyasanur forest ... - View alignment 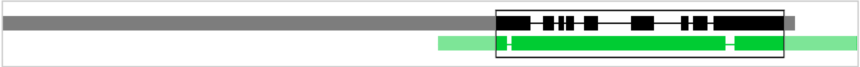	E-value: 9.7e-43<br>Score: 438<br>Ident.: 40.0%		
A0A2R2WVK2_KFDV - Envelope protein - Kyasanur forest ... - View alignment 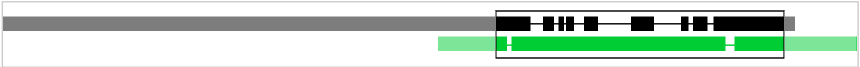	E-value: 9.7e-43<br>Score: 438<br>Ident.: 40.0%		
A0A2R2WVW9_KFDV - Envelope protein - Kyasanur forest ... - View alignment 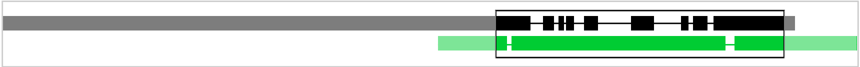	E-value: 9.7e-43<br>Score: 438<br>Ident.: 40.0%		
A0A2R2WVJ9_KFDV - Envelope protein - Kyasanur forest ... - View alignment 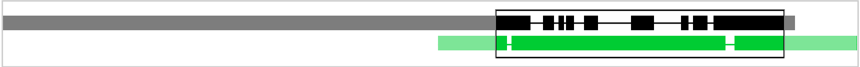	E-value: 9.7e-43<br>Score: 438<br>Ident.: 40.0%		
A0A2R2WVQ2_KFDV - Envelope protein - Kyasanur forest ... - View alignment 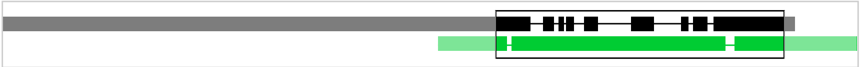	E-value: 9.7e-43<br>Score: 438<br>Ident.: 40.0%		

Job information

Query sequence[i]

KAWQVHRDW
KTILTLGDY
KTAEHLPKAW
STIGRVLEK
DYGDISLTCR
KTAEHLPKAW
KPCRIPVRAV
ASFTTQSEK
KAWQVHRDW
VTCDVGLEK
YVYDVNKITY
KTILTLGDY
REYCLHAKL
LELGGCVTL
KVEPHTGDY
LPPGDNIIY
ETKKKATGY
KTAEHLPKAW
YVYDVNKITY
IYVGELSHQW
KAWQVHRDWFEDLSL
KAWQVHRDW
PKAWQVHRD
LPKAWQVHR
RDWFEDLSL
AWQVHRDWFEDLSLP
AWQVHRDWF
PKAWQVHRD
DWFEDLSLP
WQVHRDWFEDLSLPW
AWQVHRDWF
WFEDLSLPW
FEDLSLPWR
QVHRDWFEDLSLPWR
AWQVHRDWF
VHRDWFEDLSLPWRH
EDLSLPWRH
KPSVDVWLDDIHQEN
WLDDIHQEN
AQTVVLELDKTAEH
ASTVCRRDQSDRGW
DKTAEHLPKAWQVH
DDIHQENPAKTREY
NVNVASLITPNPSM
VGLEKLKMKGMTYT
VACAKFSCETKKKA
SLTCRVTSGVDPAQ
GVPVANIEGSKYHL
FGKGSIVACAKFSC
KRPPTDSGHDTVVM
QLPPGDNIIYVGEL
AANESHSNRKTASF
SNRKTASFTTQSEK
TGSKPCRIPVRAVA
AARCPAMGPATLPE
LDDIHQENPAKTRE
RDQSDRGWGNHCGL
KKKATGYVYDVNKI
WRHEGAQEWNHADR
LTLGDYGDISLTCR
AANESHSNRKTASF
EFGEPHAVKMDIFN
GGGFVELQLPPGDN
KFAWKRPPTDSGHD
TGSKPCRIPVRAVA
SHQWFQKGSTIGRV
EPNVNVASLITPNP
KTREYCLHAKLANS

	TDSGHDTVVMEVTY<br>WFQKGSTIGRVLEK<br>TVVLELDKTAEHLP
Date of job execution	2019-05-06
Job identifier	B20190506674680338 1A1F0E0DB47453E0216320D01E8BEI (jobs are stored for 7 days)
Running time	156.3 seconds
Scheduled time	3,744 seconds
Program[i]	blastp (blastp BLASTP 2.7.1+)
Database	uniprotkb (Protein) generated for BLAST on Mar 29, 2019
Sequences	148,013,429 sequences consisting of 49,654,707,136 letters
Matrix[i]	blosum62
Threshold[i]	10
Filtered[i]	false
Gapped[i]	true
Maximum number of hits reported[i]	50

UniProt is an ELIXIR core data resource

Main funding by: National Institutes of Health; EMBL European Molecular Biology Laboratory; State Secretariat for Education, Research and Innovation SERI
